# Antibiofilm and Anti-Quorum-Sensing Activities of Novel Pyrazole and Pyrazolo[1,5-*a*]pyrimidine Derivatives as Carbonic Anhydrase I and II Inhibitors: Design, Synthesis, Radiosterilization, and Molecular Docking Studies

**DOI:** 10.3390/antibiotics12010128

**Published:** 2023-01-09

**Authors:** Ahmed Ragab, Sawsan A. Fouad, Yousry A. Ammar, Dina S. Aboul-Magd, Moustafa S. Abusaif

**Affiliations:** 1 Department of Chemistry, Faculty of Science (Boys), Al-Azhar University, Nasr City, Cairo 11884, Egypt; 2 Department of Chemistry, Faculty of Science (Girls), Al-Azhar University, Nasr City, Cairo 11754, Egypt; 3 Drug Radiation Research Department, National Center for Radiation Research and Technology (NCRRT), Egyptian Atomic Energy Authority, Egypt

**Keywords:** antibacterial, antibiofilm, anti-QS activity, gamma radiation, *h*CA-I and -II inhibition, molecular docking

## Abstract

Nowadays, searching for new anti-infective agents with diverse mechanisms of action has become necessary. In this study, 16 pyrazole and pyrazolo[1,5-*a*]pyrimidine derivatives were synthesized and assessed for their preliminary antibacterial and antibiofilm activities. All these derivatives were initially screened for their antibacterial activity against six clinically isolated multidrug resistance by agar well-diffusion and broth microdilution methods. The initial screening presented significant antibacterial activity with a bactericidal effect for five compounds, namely **3a**, **5a**, **6**, **9a**, and **10a**, compared with Erythromycin and Amikacin. These five derivatives were further evaluated for their antibiofilm activity against both *S. aureus* and *P. aeruginosa*, which showed strong biofilm-forming activity at their MICs by >60%. The SEM analysis confirmed the biofilm disruption in the presence of these derivatives. Furthermore, anti-QS activity was observed for the five hybrids at their sub-MICs, as indicated by the visible halo zone. In addition, the presence of the most active derivatives reduces the violacein production by CV026, confirming that these compounds yielded anti-QS activity. Furthermore, these compounds showed strong inhibitory action against human carbonic anhydrase (*h*CA-I and *h*CA-II) isoforms with IC_50_ values ranging between 92.34 and 168.84 nM and between 73.2 and 161.22 nM, respectively. Finally, radiosterilization, ADMET, and a docking simulation were performed.

## 1. Introduction

One of the biggest challenges in the healthcare sector is drug resistance in pathogenic bacteria. The efficiency of commonly used antibiotics against pathogenic bacteria has decreased because of the development of multidrug resistance [1]. The antimicrobial resistance (AMR) phenomenon has been called “a slow tsunami” because it can blow away all available antibiotic treatments. The COVID-19 pandemic has significantly contributed to the rise of AMR [2] because of the increasing rate at which antibiotics were prescribed to hospitalized patients, despite only one-third of those cases having a known causative agent [3]. Furthermore, COVID-19 containment campaigns resulted in the overuse of sanitizers and biocides, resulting in cross-resistance and a reduction in sensitivity to different antibiotics [4,5]. The scientific community has been alerted to several cases of secondary infections caused by *Pseudomonas aeruginosa* and *Staphylococcus aureus* bacteria [6,7]. Biofilm formation is considered one of the main mechanisms that are used by bacteria to develop such resistance patterns [8]. It is well known that biofilm formation reduces the sensitivity of bacteria to antibiotics [9,10]. The biofilm formation is controlled mainly by the cell-to-cell communication type, called the quorum-sensing (QS) process. This process occurred through the production, release, and detection of signaling molecules by the bacterial cells termed as autoinducers belonging to the acetyl homoserine lactone (AHL) group [11].

Recently, controlling bacterial infections could be achieved by interfering with the QS mechanisms as an alternative approach by reducing the production of, diffusing, and/or destroying the QS signals or by inhibiting their receptors by mimicking the signal structure [12]. Through the quorum-sensing system, the bacteria can perform several tasks, such as migration to more-favorable environments, biofilm matrix formation, virulence genes’ expression, bioluminescence, and violacein pigment production [13]. Inhibiting bacterial QS systems via finding new ways is a known strategy to develop new anti-infective agents that might produce a response within the host defense system, even in low-density bacteria [14,15,16]. QS has been identified in bacteria such as *Chromobacterium violaceum*, *P. aeruginosa*, *Enterobacter agglomerans*, and *S. aureus* [17,18,19]. Carbonic anhydrases (CAs) are ubiquitous metalloenzymes, present in most living organisms and encoded by eight evolutionary unrelated genes families (α, β, γ, δ, η, θ, ζ, ι CAs) [20,21]. All human CAs are related to the α-family, and till today, 15 isoforms have been detected [22]. They differ in their molecular features, oligomeric arrangement, cellular localization, distribution in the organs and tissues, kinetic properties, and response to the different inhibitors [23]. Carbonic anhydrases are among the newest macromolecules and are also named a metalloenzyme that affects micro-organisms’ growth or makes them vulnerable to host defenses [21,24,25]. CAs have essential physiological roles in various tissues—for instance, pH and CO_2_ homeostasis, respiration, and electrolyte secretion in different tissues and organs and in the biosynthetic reactions (e.g., gluconeogenesis, lipogenesis, and ureagenesis), in addition to their role in the red blood cells. Similar to other cells, metabolism generates CO_2_, which must be eliminated from the body by being transformed into bicarbonate by the CA isoenzyme and then back into CO_2_ for exhalation by the lungs [26,27]. These enzymes catalyze the simple physiologically important reaction of bicarbonate hydration from carbon dioxide to protons: CO_2_ + H_2_O ⇄ HCO_3_^−^ + H^+^ [28]. In addition, they are involved in pH homeostasis in the transport and supply of CO_2_ or HCO_3_^−^, biosynthetic processes, secretion of electrolytes, and photosynthesis, and therefore, they are considered antibacterial drugs, such as sulfanilamide [29]. Furthermore, the two major CA isoenzymes (CA-I and CA-II) are present in high concentrations in the erythrocytes, with CA-II’s having the highest turnover rate of all CAs [30]. The inhibition of CA causes an accumulation of carbonic acid by preventing its breakdown. This results in lowering pH levels (i.e., more acidic). Balancing the equilibrium between CO_2_ and HCO_3_^−^ is essential for microbial metabolism, which is regulated by CA inhibitors [20].

**Figure 1:**
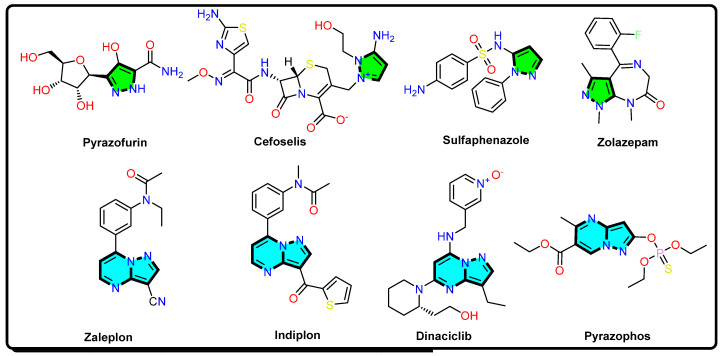
Structures of drugs containing pyrazole and pyrazolo[1,5-*a*]pyrimidine nucleus.

Pyrazole scaffold is an adaptable molecule that has attracted the attention of medicinal scientists thanks to its wide range of various pharmacological properties, such as analgesic [31], anti-inflammatory [32,33], antimicrobial [34,35], antiviral [36,37,38], antidiabetic [39,40], and antitubercular [41,42] activities; carbonic anhydrase inhibitors [22], and α-glucosidase inhibitory activity [43]. Moreover, the pyrazole pharmacophore has been applied predominantly in the pharmaceutical field and found in several FDA-approved pharmaceutical drugs, such as phenylbutazone [44] and rimonabant [45], and in many COX-II inhibitors (Celecoxib and Lonazolac) [46,47] and anticancer drugs (Crizotinib) [48]. Additionally, many pyrazole nuclei have been observed with fungicidal (Bixafen, Sedaxane, Isopyrazam, and Fluxapyroxad) [49] and antibacterial (Sulfaphenazole, Cefoselis, and Pyrazofurin) potencies [50,51] and anesthetic properties (Zolazepam) [52] (Figure 1). Furthermore, pyrazolo[1,5-*a*]pyrimidines are known to be purine analogs with diverse biological applications as antimetabolites in purine biochemical interactions: antischistosomal, antitrypanosomal, sedative [53] COX-1, and COX-2 selective inhibitors [54]; antimalarial and antifungal activities [55]; antimicrobial [56] and KDR kinase inhibitors [57]; HIV reverse transcriptase inhibitors [58]; HCV inhibitors [59]; and acetylcholinesterase and α -amylase inhibitors [60]. Several marketed drugs have pyrazolo[1,5-*a*]pyrimidine nuclei, such as ocinaplon, dinaciclib, anagliptin, pyrazophos, zaleplon, lorediplon, indiplon, and dorsomorphin (Figure 1) [61,62,63].

The sterilization of pharmaceutics is required for them to be safely and effectively used. It is a unique critical procedure. Anonymous “sterilization” is defined as the use of a suitably designed, validated, and controlled process to inactivate or reduce the viable micro-organisms, determined by the sterility assurance level (SAL) < 10^−6^, within the target product [64]. One of the most widely used methods is gamma radiation via using a source of radiation such as cobalt-60 (^60^Co) [65]. The use of gamma radiation has many advantages, such as its easy penetration into the product, resulting in better certainty of sterility, effectiveness independent of temperature and pressure [66,67]. Developing potent therapeutic agents from heterocyclic motifs has been the focus of our research group’s previous and ongoing efforts [68,69,70,71]. Furthermore, this study aims to present novel pyrazole and pyrazolo[1,5-*a*]pyrimidine derivatives that possess different groups suggested to enhance antimicrobial activity. The designed derivatives were evaluated against six bacterial isolates to determine their activity as MDR antibacterial agents. Additionally, the most active derivatives were evaluated for their antibiofilm, anti-infective, and anti-QS activities. Moreover, the mode of action for each of these derivatives was tested on two human carbonic anhydrase CA (I and II) isoforms as a target against different pathological conditions. Finally, the docking simulation, physicochemical properties, medicinal chemistry, and toxicity prediction were performed to calculate the binding energy of complexes and predict ADMET properties.

## 2. Results and Discussion

### 2.1. Chemistry

**Scheme 1:**
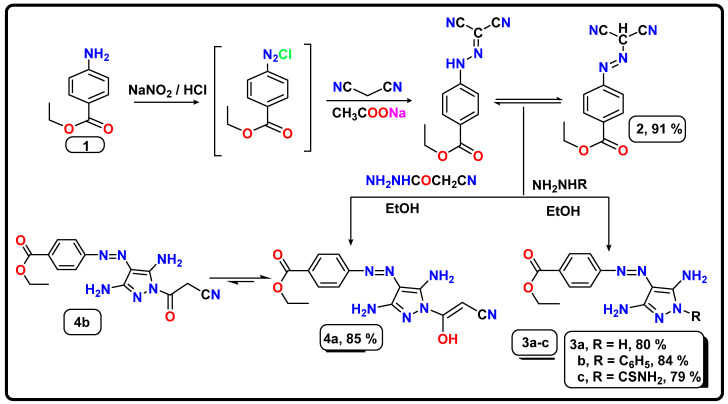
Illustration of the synthesis of 3,5-diamino pyrazole derivatives **3a**–**c** and **4a**,**b**, starting with the ethyl 4-aminobenzoate moiety.

**Scheme 2:**
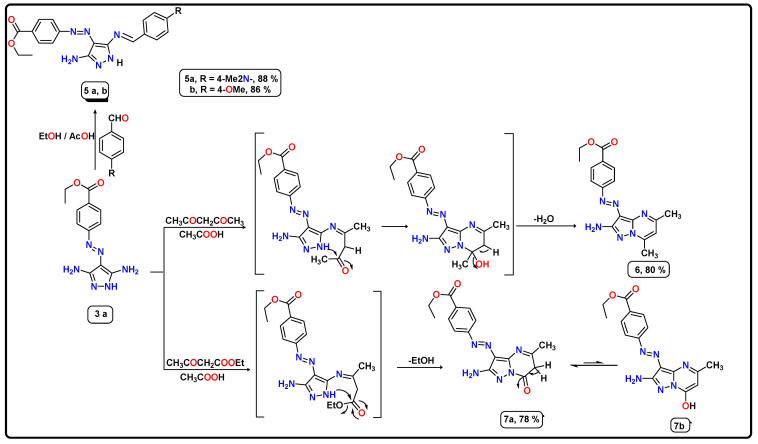
Synthesis of pyrazole Schiff bases **5a**,**b** and suggested pathway to produce 5-methylpyrazolo[1,5-*a*]pyrimidine derivatives **6** and **7**.

**Scheme 3:**
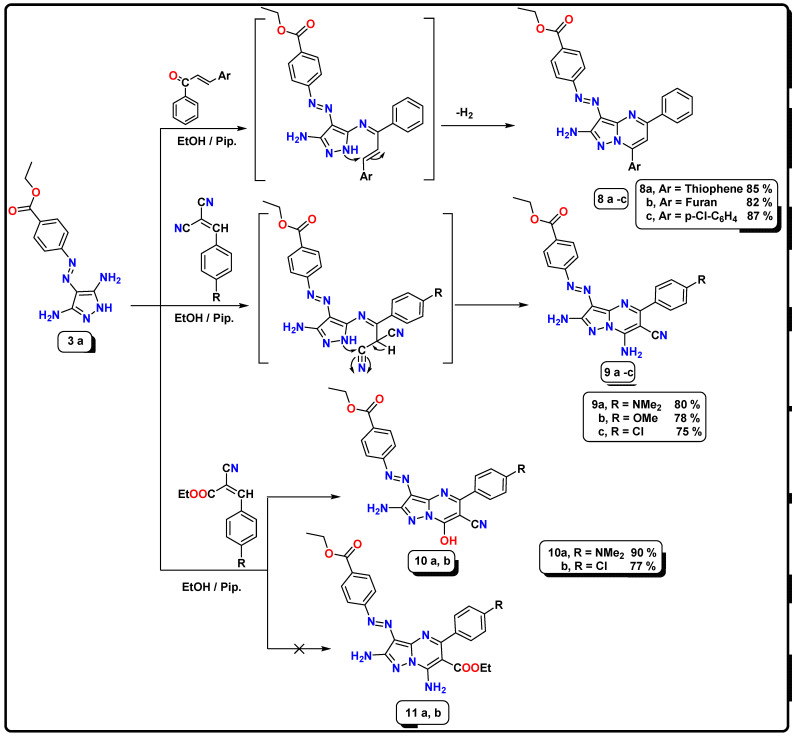
Illustration of the pathway for the reaction of 3,5-diamino pyrazole **3** with some chalcone, arylidenemalonitrile, and ethyl cyanocinnamate derivatives.

The synthetic routes used to prepare the novel azo-pyrazole derivatives **3**–**11** are viewed in Scheme 1, Scheme 2 and Scheme 3. First, diazotization of ethyl 4-aminobenzoate (**1**) proceeded by coupling its diazonium salt with dicyanomthane catalyzed with sodium acetate to afford the corresponding intermediate ethyl 4-((dicyanomethyl)diazenyl)benzoate (**2**). Further, condensation of the intermediate **2** with hydrazide reagents as hydrazine hydrate, phenylhydrazine, and thiosemicarbazide in ethanolic solution for 2–4 h produced the desired 3,5-diamino pyrazole derivatives **3a**–**c**; see Scheme 1.

The designed structures were characterized and identified in accordance with analytical and spectral data. The structure of ethyl 4-((3,5-diamino-1*H*-pyrazol-4-yl)diazenyl)benzoate (**3a**) was used as an illustrative example. Its IR spectra displayed the absence of cyano groups and the presence of strong absorption frequencies at *υ* 3390, 3298, and 3178 cm^−1^, related to two NH_2_ and NH groups, in addition to two bands at *υ* 1716 and 1562 cm^−1^, corresponding to carbonyl ester and azo groups, respectively. The ^1^H NMR spectra revealed two sharp singlet signals attributed to two amino functions at *δ* 5.95 and 6.43 ppm, a singlet signal that was due to the NH group’s being deshielded at *δ* 10.65 ppm. At the same time, the aromatic protons appeared as two doublet signals thanks to four aromatic protons at *δ* 7.61 and 7.92 ppm with the same coupling constant (*J* = 7.6 and 8.0 Hz), in addition to two aliphatic signals related to ethoxy protons, and appeared as triplet and quartet signals at *δ* 1.29 and 4.28 ppm thanks to CH_3_ and OCH_2_, respectively. The ^13^C NMR of compound **3a** confirmed the structure by using signals of aliphatic carbons at *δ* 14.84 and 61.04 ppm for the ethoxy group, and two signals referred to carbonyl ester and C-NH_2_ pyrazole at *δ* 165.94, 157.96 ppm respectively, in addition to the aromatic carbons ranging between *δ* 116.39 and 130.49 ppm.

Furthermore, the cyclization of compound **2** with cyanoacetohydrazide in ethanolic solution under reflux conditions produced the corresponding 3,5-diaminopyrazole derivatives, either **4a** or **4b**, but the spectral data IR, ^1^H NMR, and ^13^C NMR confirmed the formation of compound **4a** (enol form) as the possible structure; see Scheme 1. The IR spectrum showed absorption bands thanks to amino, cyano, and carbonyl groups at *υ* 3468, 3410, 3264, 3129, 2229, and 1712 cm^−1^. In addition, ^1^H NMR spectra showed two exchangeable singlet D_2_O signals at *δ* 6.31 and 8.20 ppm attributed to the NH_2_ and hydroxyl groups; the methine proton appeared at *δ* 7.05 ppm; and the ethoxy group appeared as triplet and quartet signals at *δ* 1.32 and 4.33 ppm, respectively.

As mentioned in Scheme 2, the 3,5-diamino pyrazole derivative **3a** was employed as the starting material for synthesizing bioactive compounds. So the reaction of compound **3a** with various aldehydes, such as 4-(dimethylamino)benzaldehyde and 4-chlorydobenzaldehe, in ethanolic solution that were catalyzed with drops of acetic acid afforded the corresponding Schiff base derivatives **5a**,**b**. The IR spectrum of compound **5a** showed absorption bands at *ν* 3394, 3282, 3203, and 1715 cm^−1^ thanks to the amino, NH, and carbonyl groups, respectively. Moreover, the ^1^H NMR spectrum demonstrated the absence of the signal of one amino group that formed Schiff base and the presence of the other amino group at *δ* 5.95 ppm as an exchangeable D_2_O singlet signal, in addition to three singlet signals at *δ* 3.33, 8.99, and 10.60 ppm that were attributed to *N*, *N*-dimethyl amino, methinic-CH, and NH proton, respectively. On the other hand, the ^1^H NMR spectrum of compound **5b** presented triplet and quartet splitting signals for CH_3_ and OCH_2_ ester at *δ* 1.30 and 4.29 ppm, respectively, and a significant singlet signal for the methoxy group at *δ* 3.81 ppm. Also present were two exchangeable singlet D_2_O signals at *δ* 7.24 and 9.82 ppm thanks to the NH_2_ and NH groups and aromatic protons ranging between *δ* 7.07 and 8.00 ppm for four doublet signals with a coupling constant of *J* = 6.8 Hz. Additionally, the ^13^C NMR spectrum of compound **5b** was confirmed by three singlet signals shielded (upfield) at *δ* 14.64, 55.91, and 61.33 ppm assignable to methyl, methoxy, and OCH_2_ carbons, respectively; signals at *δ* 156.88, 157.94, 162.79, and 165.94 ppm related to the C-NH_2_ carbon, CH=N, C-OCH_3_, and carbonyl groups, respectively; and aromatic carbons between *δ* 114.41 and 132.16 ppm.

Furthermore, the 3,5-diamino pyrazole derivative **3a** was reacted with different dicarbonyl compounds (acetylacetone and ethyl acetoacetate), where the reaction proceeded through the nucleophilic attack of the amino group at C3 of pyrazole derivative **3a** on the keto function of either acetylacetone or ethyl acetoacetate, followed by intermolecular cyclization, followed by water or ethanol elimination, respectively (Scheme 2), to afford the corresponding pyrazolo[1,5-*a*]pyrimidine derivative **6** and **7a**. The ^1^H NMR spectral data of compound **6** showed new singlet signals of the pyrimidine ring that produced at *δ* 1.87, 2.52, and 6.92 ppm thanks to two methyl at pyrimidine nucleus and CH-pyrimidine, respectively, as well as exchangeable triplet, quartet, and D_2_O singlet signals for ethoxy and NH_2_ groups that displayed at *δ* 1.31, 4.30, and 7.30 ppm, respectively. Additionally, four aromatic protons appeared as two doublet signals at *δ* 7.81 and 7.99 ppm with the same coupling constant (*J* = 6.8 Hz). On the other hand, the ^1^H NMR data of compound **7a** revealed three signals shielded at *δ* 1.32, 2.32, and 4.30 ppm related to CH_3_ ester, CH_3_ pyrimidine, and OCH_2_ ester, respectively; broad singlet signals for CH_2_ pyrimidone at *δ* 3.42 ppm; and a singlet signal observed at *δ* 6.74 ppm for the amino group, which was exchangeable with D_2_O, and the aromatic protons as two doublet signals at 7.90 and 8.02 ppm with a coupling constant of *J* = 6.4 and 5.2 Hz. The ^13^C NMR spectral of compound **6** was characterized by the presence of singlet signals related to three methyl groups and OCH_2_ carbons, observed at *δ* 14.64, 17.06, 24.32, and 61.07 ppm, respectively; a singlet signal at 116.19 thanks to CH-pyrimidine carbon; a signal at *δ* 166.12 ppm attributed to carbonyl ester; and signals between *δ* 111.13 and 147.66 ppm that were assignable to aromatic carbons (phenyl and pyrazolopyrimidine).

The heterocyclization of the 3,5-diaminopyrazole derivative **3a** toward an activated double bond (α, β-unsaturated carbonyl system, arylidinemalononitrile, and ethyl cyanocinnamate derivatives) afforded the prospect of producing many derivatives pyrazolo[1,5-*a*]pyrimidines. At first, the interaction of the starting material **3a** with three types of α, β-unsaturated carbonyl compounds such as 1-phenyl-3-(thiophen-2-yl)prop-2-en-1-one, 3-(furan-2-yl)-1-phenylprop-2-en-1-one, and 3-(4-chlorophenyl)-1-phenylprop-2-en-1-one afforded the corresponding products, which were confirmed according to the elemental and spectral data as ethyl 4-((2-amino-5-phenyl-7-(aryl-2-yl)pyrazolo[1,5-*a*]pyrimidin-3-yl)diazenyl)benzoate **8a**–**c** (Scheme 3). The mechanism of this reaction proceeds through the condensation of the amino group at C3 of pyrazole **3a** with the carbonyl group of the α and β-unsaturated carbonyl system, followed by intermolecular cyclization by the nucleophilic attack of the endocyclic NH group on the activated double bond; then it loses a hydrogen molecule through spontaneous oxidation under reaction conditions.

The IR spectra of compound **8a** displayed bands at *ν* 3438, 3266, 1709, 1613, and 1566 cm^−1^ that were assignable to NH_2_, carbonyl ester, C=N, and N=N groups, respectively. Its ^1^H NMR data showed the presence of two singlet signals corresponding to NH_2_ and pyrimidine-CH at *δ* 6.28 and 7.15 ppm, respectively; signals shielded at *δ* 1.32 and 4.31 ppm were related to ethyl protons of ester and aromatic protons ranging between *δ* 6.21 and 8.19 ppm. Moreover, to confirm the structure of compound **9a**, its IR spectrum revealed bands at *ν* 3396, 3304, 2211, 1714, and 1565 cm^−1^ assigned to NH_2_, CN, C=O, C=N, and N=N groups, respectively. Additionally, the ^1^H NMR data showed a new singlet signal at *δ* 3.07 ppm related to the dimethyl amino protons, two D_2_O exchangeable singlet signals at *δ* 6.37 and 6.84 ppm assigned to two amino groups, and eight aromatic protons ranging between 7.73 and 8.02 ppm. Moreover, the ^13^C NMR revealed four aliphatic signals at *δ* 14.76, 14.76, 14.76, and 61.14 ppm owing to ethoxy and dimethyl amino carbons. In addition, signals at *δ* 154.82, 156.98, 159.25, and 166.34 were attributed to two C-NH_2,_ C=N, and C=O carbons, and the signals of aromatic carbons ranged from *δ* 111.96 to 133.94 ppm.

Finally, in the reaction of the 3,5-diaminopyrazole **3a** with ethyl cyanocinnamate derivatives in ethanolic solution catalyzed by piperidine and two different products can be obtained as7-hydroxy-pyrazolo[1,5-*a*]pyrimidine derivatives **10a**,**b** or 7-amino-pyrazolo[1,5-*a*]pyrimidine derivatives **11a**,**b**, but the analytic and spectral data confirmed that the formation of 7-hydroxy-pyrazolo[1,5-*a*]pyrimidine derivatives is a sole product (Scheme 3). The IR spectral of compound **10a** presented characteristic bands at *ν* 3394, 3300, 3208, and 2218 cm^−1^ related to the hydroxy, amino, and cyano groups. Additionally, the ^1^H NMR showed three singlet signals at *δ* 3.01, 6.92, and 10.27 ppm assigned to NMe_2_, NH_2_, and OH protons, respectively; showed two signals of ethyl protons of ester as a triplet and a quartet at *δ* 1,31 and 4.30 ppm, respectively; and showed aromatic protons. The ^13^C NMR of compound **10a** revealed signals at *δ* 44.09, 116.26, 155.16, 160.05, and 166.10 ppm, corresponding to NMe_2_, CN, C-NMe_2_, C-OH, and C=O carbons, respectively; it revealed signals at *δ* 14.76 and 60.93 ppm thanks to the ethoxy group; and signals for aromatic carbons ranged between 117.41 and 135.77 ppm.

### 2.2. Biological Activity

#### 2.2.1. Screening of Synthesized Compounds for Antibacterial Activity

The preliminary screening for the now 16 derivatives was performed against Gram-positive and Gram-negative bacteria that were clinically isolated from different clinical samples. Erythromycin (E) and Amikacin (AMK) antibiotics were used in the assay as the positive controls. The average zone inhibition (AZOI) was recorded in mm (Table 1). According to the obtained findings, the most significant antibacterial activities were observed with five compounds, namely 3a, 5a, 6, 9a, and 10a, with AZOIs ranging from 28 mm to 32 mm against the bacterial isolates tested. The remaining compounds showed moderate antibacterial activity. The standard antibiotics showed resistant activity, with AZOIs ranging from 10.5 mm to 12 mm for Erythromycin and from 11 mm to 14 mm for Amikacin. These results indicate that most of the newly designed compounds appear to be effective at combating infectious bacteria.

#### 2.2.2. MIC and MBC Determination

The active pyrazole and pyrazolo[1,5-*a*]pyrimidine derivatives **3**–**10** with AZOIs > 10 mm were assayed in vitro to determine their minimum inhibitory concentrations (MICs) and minimum bactericidal concentrations (MBCs) against the tested pathogens, by using the broth microdilution method (Table 2). The MIC assay confirmed the preliminary antibacterial screening with the five agents classified as two pyrazoles **3a** and **5a** and three pyrazolo[1,5-*a*]pyrimidine derivatives **6**, **9a** and **10a**, which have shown excellent antibacterial activities. These derivatives exhibited MIC values ranging from 0.125 to 0.50 µg/mL and MBC values between 0.062 and 0.25 µg/mL against Gram-positive isolates and displayed MIC values between 0.062 and 0.50 µg/mL and MBC values from 0.062–0.50 µg/mL against Gram-negative isolates.

It was noticed that 3,5-pyrazole compound **3a** exhibited the highest MIC values and was effective more than positive controls against Gram-positive strains (MIC = 0.125 µg/mL) and Gram-negative bacteria (MIC ranging between 0.062 and 0.25 µg/mL) among the other derivatives, as well as the standard antibiotics, over all the tested isolates. Moreover, the three pyrazolo[1,5-*a*]pyrimidine derivatives **6**, **9a**, and **10a** that also recorded potent antibacterial activity against the different isolates tested with MICs range between 0.187 and 0.50 µg/mL and MBC values between 0.094 and 0.25 µg/mL. In contrast, pyrazole–Schiff base **5a** has MICs between 0.25 and 0.50 µg/mL and MBCs values between 0.125 and 0.25 µg/mL. The remaining pyrazole derivatives exhibited moderate potency compared with the reference antibiotics against the pathogenic isolates. The antibiotics used displayed resistant MICs values between 8 µg/mL and 64 µg/mL and between 32 µg/mL and 128 µg/mL with Erythromycin and Amikacin, respectively.

In terms of the structure–activity relationship (SAR), introducing any group at N1 of 3,5-diamino-1*H*-pyrazole derivative **3a** decreased the activity in general. As represented in Table 2, replacing the hydrogen at N1 with a phenyl ring to afford 3,5-diamino-1-phenyl-1*H*-pyrazole derivative **3b**, where the activity decreased overall, isolated bacteria, and that may have been related to the increase in the hydrophobic characters. Moreover, introducing hydrophilic groups as the thioamide group (S=C-NH_2_) or the cyanoacetamide moiety (O=C-CH_2_CN) at the same position N1 of pyrazole as derivatives **3c** and **4** suppressed the bacterial growth but still less so than pyrazole derivative **3a**, indicating that the presence of NH at pyrazole is beneficial to antibacterial potency. Moreover, modifying the pyrazole moiety by forming a new azomethane group (C=N) containing the hydrophobic aryl moiety exhibited good antibacterial activity compared with Erythromycin and Amikacin. It is interesting to observe that dimethyl amine as compound **5a** displayed more-effective antibacterial activity than the methoxy group as compound **5b** at the para position from two to four folds against all tested isolates. This good potency may be attributed to the electron-donating electrons (OME and N(Me)_2_) pushing the electron toward the pyrazole nucleus. The 5-amino-3-((4-(dimethylamino)benzylidene)amino)-1*H*-pyrazole derivative **5a** demonstrated the lowest MIC value of 0.25 µg/mL against *S. aureus* and *E. coli* and showed MIC values of 0.50 µg/mL against other isolates with 32–256 folds higher than positive controls.

As expected, the pyrazolo[1,5-*a*]pyrimidine derivatives showed a favorable improvement against the bacterial isolates (Table 2). Compounds **6** and **7** had the same pharmacophore core, except for the replacement of methyl group at C7 of pyrazolo[1,5-*a*]pyrimidine derivative with a hydroxyl group. Generally, this change in structure by the more electron-donating group (OH) decreases the activity by nearly 5–6 times against all isolates. The 5,7-dimethyl-pyrazolo[1,5-*a*]pyrimidine derivative **6** showed a good antibacterial spectrum (MICs = 0.187–0.50 µg/mL) compared with 5-methyl-7-hydroxy-pyrazolo[1,5-*a*]pyrimidine derivative **7**, which displayed inhibitory activity (MICs = 1–2 µg/mL). Moreover, the 5,7-dimethyl-pyrazolo[1,5-*a*]pyrimidine derivative **6** was more effective against *S. aureus*, *E. faecalis*, and *P. aeruginosa* (MIC = 0.187–0.375 µg/mL).

Different segments were further explored for their effect on antibacterial activity. The 5,7-diaryl pyrazolo[1,5-*a*]pyrimidine derivatives **8a**–**c** exhibited good performance against tested isolates, indicating that the presence of a heterocyclic scaffold such as furane or thiophene had higher inhibitory activity than the aromatic aryl group as 4-chlorophenyl, and the activity of the tested derivatives decreased in the following order: **8a** (thiophene) > **8b** (furane) > **8c** (4-chlorophenyl). These derivatives demonstrated inhibitory potencies toward tested isolates at low concentrations (MICs = 2–8 µg/mL) against Gram-positive and Gram-negative. In addition, replacing the aryl group at position C7 at pyrazolopyrimidine with amino or hydroxyl groups caused enhanced antibacterial activity and hydroxy more active than their respective analog NH_2_.

On the other hand, by examining data obtained for these two series **9a**–**c** and **10a**,**b**, it was found that the presence of dimethylamine is the most effective fragment and suppresses bacterial growth with MIC values between 0.125 and 4.0 µg/mL for **9a**–**c** and MICs = 0.125–2.0 µg/mL for **10a**,**b**. Moreover, the 2,7-diamino pyrazolo[1,5-*a*]pyrimidine derivative **9a** showed good inhibitory potencies against *E. faecalis*, *S. aureus*, and *S. pyogenes* where MICs = 0.25–0.50 µg/mL and MBCs = 0.125–0.25 µg/mL compared with Erythromycin (MICs = 8.0–64.0 µg/mL and MBCs = 16.0–128.0 µg/mL). Notably, among the series of **9a**–**c**, the activity increased with the aryl group in the following order: 4-N(Me)_2_ > 4-OMe > 4-Cl; that could be related to the nature of both diamine and methoxy that had the hydrophilic group rather than the chloro atom, which possesses a hydrophobic nature. Moreover, compounds **9a** and **10a** that had the dimethyl amine derivative at the aryl group at C5 of pyrazolopyrimidine were found to be most potent against *E. coli*, where the MIC = 0.125 µg/mL and MBC = 0.0625 µg/mL, compared with Amikacin, where the MIC = 32.0 µg/mL and MBC = 128.0 µg/mL. Regarding the MBC determination, our designed pyrazole and pyrazolo[1,5-*a*]pyrimidine derivatives **3a**–**11** revealed prominent MBC/MIC that were ≤4, indicating their bactericidal activity as described previously.

#### 2.2.3. Antibiofilm and Anti-Infective Activities of Pyrazole and Pyrazolo[1,5-*a*]pyrimidine Derivatives

##### Crystal Violet Antibiofilm Assay

**Figure 2:**
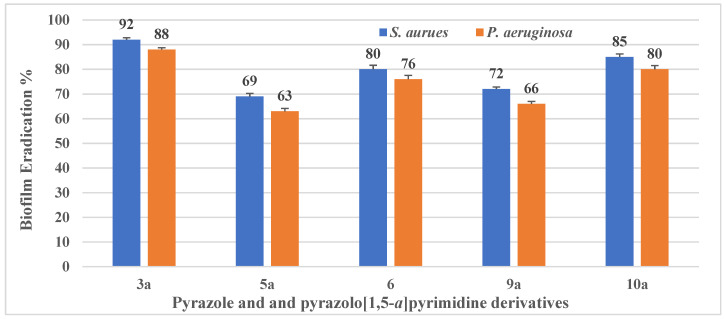
Antibiofilm activity of pyrazoles **3a** and **5a** and pyrazolo[1,5-*a*]pyrimidine derivatives **6**, **9a**, and **10a** at their MICs against *S. aureus* and *P. aeruginosa* bacterial isolates. The biofilm was quantified using the crystal violet staining assay at OD_570_.

The ability of the bacterial isolates to form biofilm was quantitively determined. Both *S. aureus* and *P. aeruginosa* showed OD_570_ ≥ 1. Thus, they are considered as strong biofilm-forming bacterial isolates. The potential of the most active pyrazole and pyrazolo[1,5-*a*]pyrimidine derivatives, namely, **3a**, **5a**, **6**, **9a**, and **10a**, to eradicate the biofilm formation by both bacterial isolates was examined. The bacteria tested were allowed to culture in the presence of each compound, and the biofilm formation was screened using the 96-well crystal violet biofilm assay. Following 24 h incubation, the most pronounced antibiofilm activity was recorded for compounds 3,5-diaminopyrazole **3a** and 7-hydroxy-pyrazolo[1,5-*a*]pyrimidine derivatives **10a**. The two compounds significantly inhibited the biofilm formation of *S. aureus* by ≥85% (*p* < 0.01) and of *P. aeruginosa* by > 80% (*p* < 0.01). Moreover, 5,7-dimethyl pyrazolo[1,5-*a*]pyrimidine derivative **6** was able to reduce the *S. aureus*’s biofilm formation by 80 ± 0.63% (*p* < 0.01) and by 76 ± 0.52% (*p* < 0.01) in the case of *P. aeruginosa*. The ability of both compounds **9a** and **5a** to inhibit the formation of *S. aureus* and *P. aeruginosa* biofilm was (72 ± 0.81% and 66 ± 0.9%, *p* < 0.01) and (69 ± 0.24% and 63 ± 0.52%, *p* < 0.01), respectively (Figure 2).

Finally, the most active derivatives, namely **3a**, **5a**, **6**, **9a**, and **10a**, showed the ability to diminish biofilm formation. Therefore, we suggested that these derivatives have anti-infective and antibiofilm properties after we attenuated virulence factors of *S. aureus* and *P. aeruginosa* biofilm formation.

##### Determination of the Antibiofilm Activity of Pyrazole and Pyrazolo[1,5-*a*]pyrimidine Derivatives via SEM Analysis

To further characterize the effect of the most active derivatives, namely **3a**, **5a**, **6**, **9a**, and **10a**, on the *S. aureus* and *P. aeruginosa* biofilms, *S. aureus* was supplemented with each derivative at their MICs levels, and the biofilm was allowed to form on the surface of glass slides on a multiwell plate. The morphology of the compound-treated and untreated *S. aureus* and *P. aeruginosa* biofilm architecture formed on the glass slides after the 24 h incubation was visualized using SEM.

**Figure 3:**
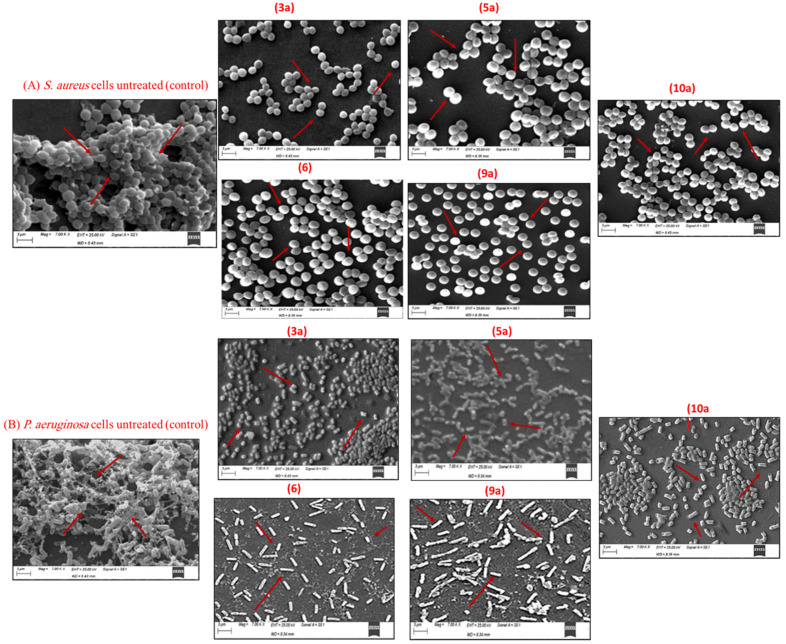
Microscopic visualization of antibiofilm activity of pyrazole compounds. (**A**) Scanning electron micrographs of *S. aureus* cells untreated (control) and treated with 1XMIC of each pyrazole derivative at 7000× magnification. (**B**) Scanning electron micrographs of *P. aeruginosa* cells untreated (control) and treated with 1XMIC of each pyrazole derivative at 7000× magnification.

As shown in Figure 3, the SEM analysis under 7000× magnification revealed a thick, dense, and fully formed biofilm consisting of multilayered, untreated *S. aureus* and *P. aeruginosa* bacterial cells. On the other hand, upon exposure to the different pyrazole compounds, the biofilm production was disrupted, and a marginal biofilm mass was detected on the slides. Furthermore, the bacteria appeared as monolayer-dispersed cells scattered on the surface. These SEM images support the data obtained from the CV biofilm assay.

##### Quorum-Sensing Inhibition Bioassay

**Figure 4:**
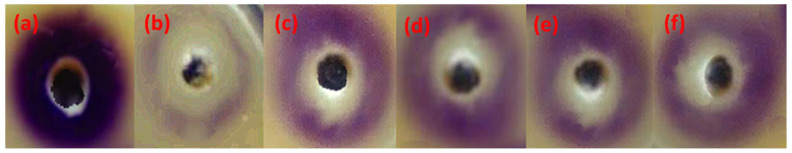
QS-inhibition assay on Soft Top agar of *C. violaceum* (CV026) biosensor strain of the most active derivatives: (**a**) CV026 pigment with DMSO served as negative control with no anti-QS activity; (**b**) anti-QS effect of 3,5-diaminopyrazole derivative **3a** at 0.0625 µg/mL; (**c**) anti-QS effect of pyrazole derivative **5a** at 0.187 µg/mL; (**d**) anti-QS effect of compound **6** at 0.125 µg/mL; (**e**) anti-QS of compound **9a** at 0.187 µg/mL; and (**f**) anti-QS effect of compound **10a** at 0.0625 µg/mL.

Quorum sensing (QS), a regulator for the virulence gene expression machinery in a wide variety of pathogens, has attracted attention as a novel antivirulence target [19]. The quorum-quenching activity against a biofilm-forming *S. aureus* and *P. aeruginosa* was evaluated previously using reporter bacterial strains (e.g., *E. coli* pSB1075, *E. coli* pSB401, and *C. violaceum* (CV026)). In the present study, the ability of the most active pyrazoles and pyrazolo[1,5-*a*]pyrimidines to inhibit QS were thoroughly investigated against the biofilm-forming *S. aureus* and *P. aeruginosa* using the CV026 biosensor reporter strain as an indicator strain. This anti-QS inhibitory activity against CV026 was observed with the five most active pyrazole and pyrazolo[1,5-*a*]pyrimidine derivatives hybrids, namely **3a**, **5a**, **6**, **9a**, and **10a**, at 0.0625, 0.187, 0.125, 0.187, and 0.0625 µg/mL, respectively, with the formation of a visible halo zone (growth without pigmentation), as shown in Figure 4. The anti-QS activity was also found to reduce at lower concentrations of the derivatives.

**Figure 5:**
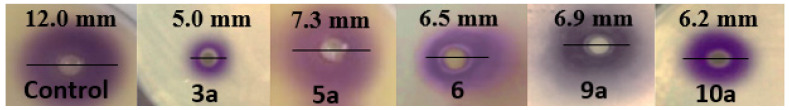
Anti-QS activity via signal inhibition in *S. aureus*. Control well, QS signals produced exogenously and diffused through *S. aureus*; these molecules were traced with purple pigmentation, produced by CV026 in Soft Top agar. In the presence of the sub-MICs of the compounds **3a**, **5a**, **6**, **9a**, and **10a**, bacterial culture (10^6^ CFU/mL) was used, and the QS signal diffusion zone was decreased compared with the control well (mean ± SD, *p* < 0.05).

**Figure 6:**
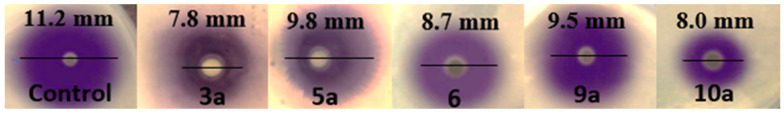
Anti-QS activity via signal inhibition in *P. aeruginosa*. Control well, QS signals produced exogenously and diffused through *P. aeruginosa*; these molecules were traced with purple pigmentation, produced by CV026 in Soft Top agar. In the presence of the sub-MICs of the compounds **3a**, **5a**, **6**, **9a**, and **10a**, bacterial culture (10^6^ CFU/mL) was used, and the QS signal diffusion zone was decreased compared with the control well (mean ± SD, *p* < 0.05).

After evaluating QSI’s ability in pyrazoles **3a** and **5a** and pyrazolo[1,5-*a*]pyrimidine derivatives **6**, **9a**, and **10a** in the biosensor strain, the activity was studied on *S. aureus* and *P. aeruginosa*. The results indicated that both the *S. aureus* and *P. aeruginosa* populations produced and diffused QS signal molecules during growth. In addition, they indicated that CV026 could produce the violacein pigments in response to those exogenously signal molecules upon their culture next to each other. In the presence of the sub-MICs of pyrazole derivatives, the signal diffusion was decreased, and this was demonstrated by a decrease in the violacein pigment production in CV026. These findings confirm the QS inhibitory activity of the most active derivatives tested (Figure 5 and Figure 6).

#### 2.2.4. CA-I and CA-II Isoenzymes Purification and Inhibition

**Figure 7:**
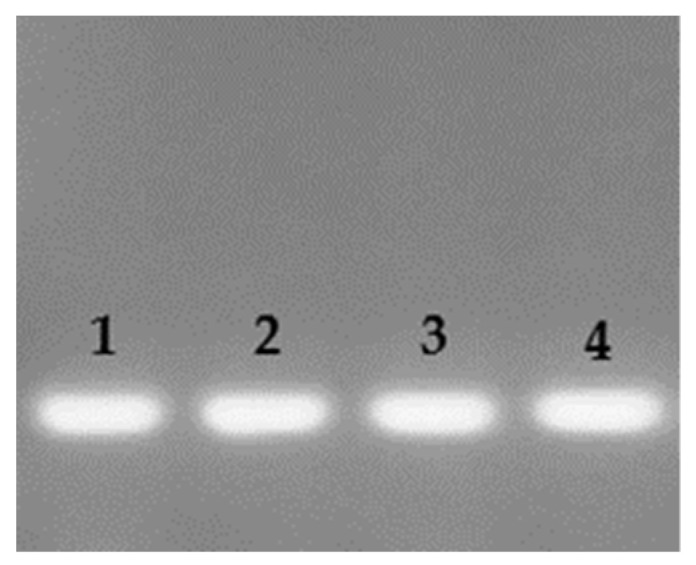
SDS-polyacrylamide gel electrophoresis of *h*CA-I and *h*CA-II purified by Se-pharose-4B-L-tyrosine-sulfanilamide affinity gel chromatography (Lanes 1 and 2 are for *h*CA-I; and Lanes 3 and 4 are for *h*CA-II).

Human erythrocyte carbonic anhydrase I and II were purified from hemolysate using affinity chromatography on Sepharose-4B-L-tyrosine-sulfanilamide. SDS-PAGE gels revealed that both *h*CA-I and -II isoenzymes migrated as a single band (Figure 7). The overall purification yield of *h*CA-I and -II was assumed as 55% and 62%, the specific activity was 950 and 7500 EU/mg protein, and these enzymes were purified 100 and 900 times, respectively, as shown in Table 3.

Moreover, the researchers usually used the IC_50_ value to describe the inhibitory effects. However, another, sufficient way has been used, which is the *K_i_* constant. The *K_i_* values were also calculated using the Lineweaver–Burk graphs. In this study, both IC_50_ and *K_i_* values parameters of the most active derivatives, namely **3a**, **5a**, **6**, **9a** and **10a**, were determined; these values are given in Table 4.

As shown in Table 4, the corresponding IC_50_ and *K_i_* values were calculated for both *h*CA isoenzymes (*h*CA-I and *h*CA-II) under in vitro conditions. It is obvious that all the active compounds inhibited both human isoforms CA-I and -II used in this study with a varying range of inhibition constants. In the case of *h*CA-I, the most significant activity was observed with compound **3a**, with an IC_50_ = 92.34 nM and *K_i_* = 88.78 nM, followed by compounds **9a** and **5a**, showing IC_50_ of 123.41 nM and 137.99 nM, alongside *K_i_* of 101.55 nM and 176.21 nM, respectively. Moreover, compound **10a** exhibited an inhibition activity against the *h*CA-I isoform, indicated by IC_50_ = 153.45 nM and *K_i_* of 143.59 nM. Further, compound **5** displayed inhibition activity with IC_50_ and *K_i_* of 168.84 nM and 153.46 nM.

On the other hand, the highest activity with *h*CA-II isoform was displayed by compound **3a** with IC_50_ = 73.2 nM and *K_i_* = 91.85 nM, followed by compounds **10a** and **6** that showed inhibition activity against the isoform with IC_50_ of 121.98 nM and 130.29 nM and with *K_i_* of 139.37 and 145.58 nM, respectively. The lowest activity shown among the five active compounds was recorded for compounds **5a** and **9a** against *h*CA-II with IC_50_ = 161.22 nM and *K_i_* = 144.27 nM and IC_50_ = 142.28 nM and *K_i_* = 142.48 nM and 135.78 nM, respectively. It should be mentioned that the five compounds exhibited more potent activity than the reference drug acetazolamide (IC_50_ = 994.78 nM and *K_i_* = 1052.22 nM) against the *h*CA-I isoform and with IC_50_ of 900.33 nM and *K_i_* of 981.64 nM against the *h*CA-II isoform. These findings indicate that *h*CA-II has a higher affinity for inhibition than the *h*CA-I. This is in accordance with previous studies [72,73], which stated that these two isozymes exhibit different affinities for the inhibitors, and finally, it can be concluded that *h*CA-II has a higher affinity for the inhibitor than *h*CA-I does.

The difference in inhibition between these two isoenzymes (*h*CA-I and -II) may be attributed to their different active site architectures—hence the presence of more histidine residues in the *h*CA-I isoform [73,74]. Additionally, in *h*CA-I, the residue His_64_ plays an important role in catalysis, in addition to the zinc metal in ligand that bonded to His119, His96, and His94. In addition, *h*CA-II contains a histidine cluster consisting of His64, His4, His3, His10, His15, and His17, which is absent in *h*CA-I [72,73].

#### 2.2.5. Effect of Gamma Sterilization on the Antimicrobial Activity of the Most Active Compounds

The sterilization of pharmaceutical agents becomes necessary to ensure these products’ safety and effectiveness. Gamma radiation is a standard method for sterilizing different pharmaceutical agents in raw or dry forms [75]. The reference absorbed dose for terminal sterilization is 25.0 kGy, according to the IAEA, to produce a Sterility assurance level (SAL) of <10^−6^ [76]. It has been stated in previous studies [77] that gamma radiation doses up to 20.0 kGy would be sufficient to produce sterility in the antimicrobial agents. The active derivatives were exposed to a wide range of gamma radiation doses (from 1.0 kGy to 20.0 kGy). It has been observed that no change occurred in either the color, odor, or solubility of the active hybrids upon radiation exposure. However, at 20.0 kGy, a charring odor was detected, accompanied by a change in color. Moreover, the chemical structures of the irradiated compounds were determined by using a UV-VIS spectrophotometer, revealing the stability of the hybrids after radiation. As shown in Table 5, the findings of this study revealed that a gamma radiation dose of 5.0 kGy was the minimum sufficient dose to sterilize the active compounds (i.e., yield a SAL of <10^−6^).

### 2.3. Molecular Docking Studies

To rationalize the promising biological evaluation and to understand the inhibition mechanism of the most active derivatives, namely **3a**, **5a**, **6**, **9a**, and **10a**, a molecular docking simulation was performed inside the active site of human carbonic anhydrase isoenzymes (*h*CA-I and -II). The docking simulation was performed inside the active site of (PDB: 2NN7 and 1H9N) for *h*CA-I and -II, separately. Moreover, our docking simulation extended to evaluate the binding energy inside the active site inside of alpha-carbonic anhydrase and beta carbonic anhydrase as a bacterial target (PDB: 5HPJ and 1I6P), separately. The docking study was performed using Molecular Operating Environmental (MOE) 2014.0901, Chemical Computing Group Inc, Montreal, QC, Canada.

#### 2.3.1. Docking Simulation inside the Active Site of hCA-I (PDB: 2NN7)

The most active pyrazole and pyrazolo[1,5-*a*]pyrimidine derivatives, namely **3a**, **5a**, **6**, **9a**, and **10a**, showed good binding energy, ranging from −5.21 to −5.77 Kcal/mol, compared with acetazolamide S = −5.31 Kcal/mol. First, the acetazolamide interacted with the active site of carbonic anhydrase I (*h*CA-I) with two hydrogen bonds, one arene–hydrogen interaction, and zinc metal ion. The oxygen of sulfone formed a hydrogen bond acceptor with backbone Thr199 with a bond length of 1.99 Å and interacted with side chain His67 with a hydrogen bond acceptor with the oxygen of the carbonyl group with a distance of 2.18 Å, while the Zn^+2^ ion coordinated with the oxygen of sulfonamide (SO_2_NH_2_), and the residue Leu198 interacted with thiadiazol ring through an arene–hydrogen interaction.

**Figure 8:**
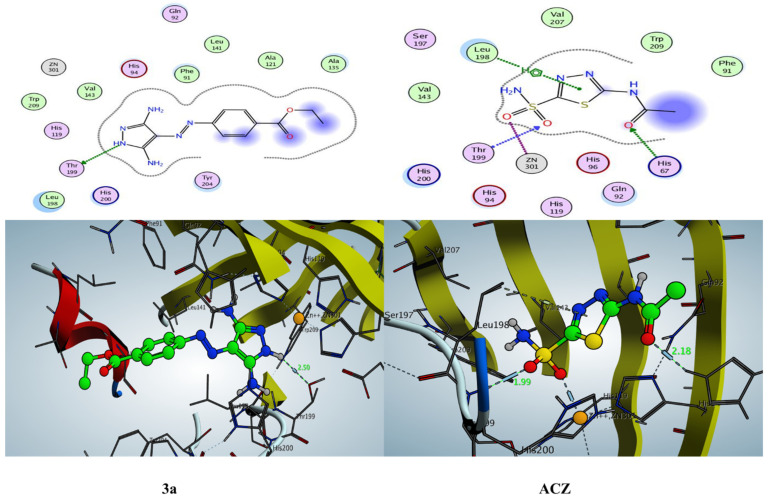
2D and 3D enzyme–ligand interaction of compounds **3a** and **ACZ** inside the active site of *h*CA-I.

Furthermore, the most active pyrazole derivative **3a** that exhibited IC_50_ = 92.34 nM and *K_i_* = 88.78 nM revealed the lowest binding energy S = −5.77 Kcal/mol and bonded to the active site with side chain Thr199 with a hydrogen bond acceptor with NH of pyrazole with a bond length of 2.50 Å; in addition, the ethyl diazophenyl benzoate interacted with the active site and formed a hydrophobic interaction with the residues Ala121, Ala135, and Tyr204 (Figure 8).

**Figure 9:**
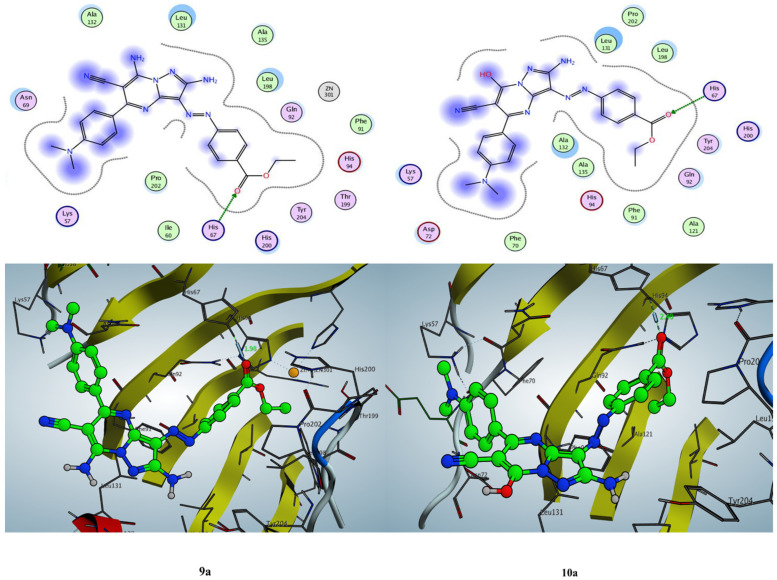
2D and 3D enzyme–ligand interaction of compounds **9a** and **10a** inside the active site of *h*CA-I.

Moreover, the docking pose of pyrazolo[1,5-*a*]pyrimidine derivatives **9a** and **10a** exhibited binding energy S = −5.51 and −5.33 Kcal/mol through only one hydrogen bond side chain acceptor between the residue His67 and the oxygen of carbonyl of ethyl ester with bond lengths of 1.98 and 2.40 Å, respectively. Both pyrazolo[1,5-*a*]pyrimidine derivatives **9a** and **10a** showed hydrophobic interaction inside the active site with whole pyrazolo[1,5-*a*]pyrimidine pharmacophore (Figure 9). Additionally, the 5-amino-3-((4-(dimethylamino)benzylidene)amino)-1*H*-pyrazole derivative **5a** demonstrated binding energy S = −5.46 Kcal/mol with a hydrogen bond acceptor between the oxygen of carboxylate with side chain residue His67 with a bond length of 2.02 Å. In addition, the pyrazole moiety and *N*,*N*-dimethyldiazophenyl formed a hydrophobic interaction with Leu131, Asp72, Phe91, Lys57, Gln92, Pro202, His94, Leu198, and His200. However, pyrazolo[1,5-*a*]pyrimidine derivative **6**, which demonstrated the higher binding energy through the most active derivatives S = −5.21 Kcal/mol, could form an arene–hydrogen interaction between the residue 198 and pyrazole nucleus of pyrazolo[1,5-*a*]pyrimidine and exhibited that there is not a hydrogen bond interaction with the residues of *h*CA-II, but a hydrophobic interaction was observed between ethyl diazophenyl benzoate with the residues Asn69, Phe70, His200, Gln92, Ala121, Leu131, and Pro202.

#### 2.3.2. Docking Simulation inside the Active Site of hCA-II (PDB: 1H9N)

**Figure 10:**
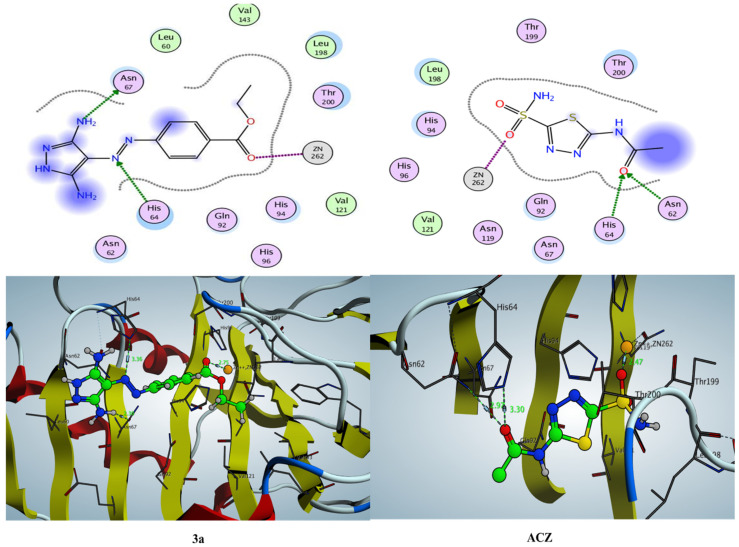
2D and 3D enzyme–ligand interaction of compounds **3a** and **ACZ** inside the active site of *h*CA-II.

The docking binding energy of the most active derivatives falls in the range from –5.31 to 4.62 Kcal/mol compared with acetazolamide (ACZ) S = −4.48 Kcal/mol; in addition, the lowest and best docking score was assigned to pyrazole derivative **3a** (S = −5.31 Kcal/mol) and the highest binding energy given to Schiff base pyrazole derivative **5a** (S = −4.62 Kcal/mol). Pyrazole derivative **3a**, which showed the lowest IC_50_ = 73.2 nM and *K_i_* = 91.85 nM, could form a good complex inside the active site of *h*CA-II with binding energy S = −5.31 Kcal/mol through two hydrogen bonds and a metallic contact bond. The first hydrogen bond formed between the amino of pyrazole at C3 and side chain residue Asn67 (H-bond donor), while the second one was between the residue His64 and the nitrogen of the diazo group (N=N) (H-bond acceptor) with bond lengths of 2.30 and 3.36 Å, respectively. Moreover, the pyrazole moiety formed a hydrophobic interaction inside the active site, and the zinc ion bonded to the carbonyl of the ester group. Additionally, the acetazolamide that was used as a positive control and that demonstrated a IC_50_ of 900.33 nM and a *Ki* of 981.64 nM revealed the binding energy S = −4.48 Kcal/mol with two hydrogen bonds between the residues His64 and Asn62 with the oxygen of carbonyl of acetamide with bond lengths of 3.30 and 2.97 Å, respectively. In addition, the zinc ion contacts one oxygen of sulfonamide derivative, as shown in Figure 10.

**Figure 11:**
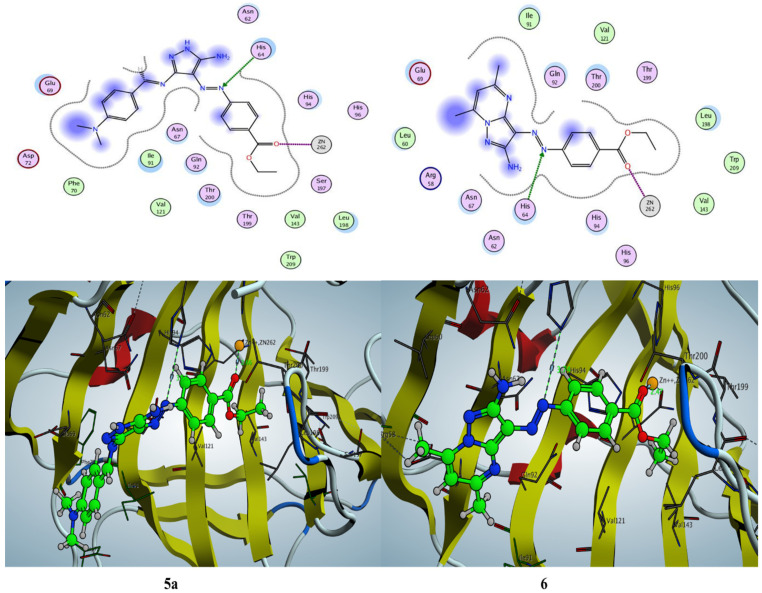
2D and 3D enzyme–ligand interaction of compounds **5a** and **6** inside the active site of *h*CA-II.

**Figure 12:**
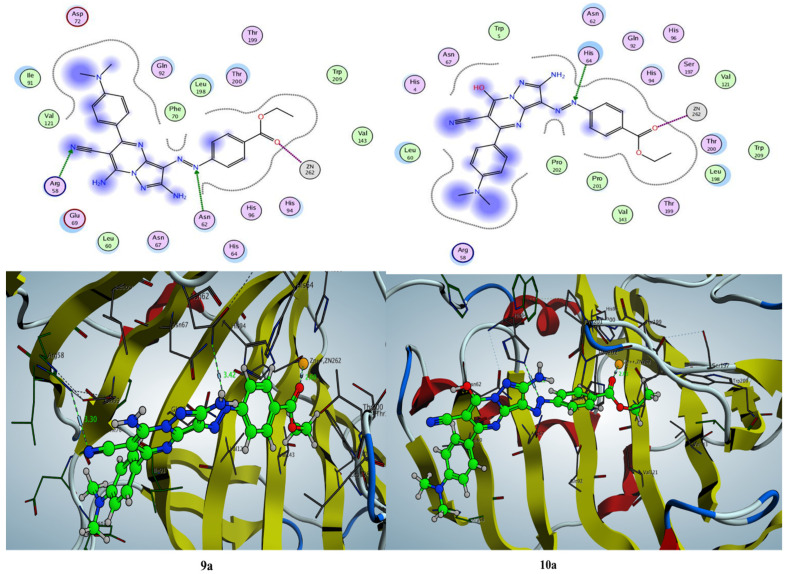
2D and 3D enzyme–ligand interaction of compounds **9a** and **10a** inside the active site of *h*CA-II.

Furthermore, the 5-aminopyrazole derivative **5a** and pyrazolo[1,5-*a*]pyrimidine derivative **6** and **10a** interacted with the active site of *h*CA-II with one hydrogen bond side chain acceptor between the residues His64 with the nitrogen of the diazoaryl group (N=N) with bond lengths of 3.66, 3.74, and 3.12 Å, respectively, and metal complex between the Zn^+2^ ion and the carbonyl group of ester, as shown in Figure 11 and Figure 12. The 2,7-diaminopyrazolo[1,5-*a*]pyrimidine derivative **9a** revealed two hydrogen bons side chain acceptors between the resides Arg58 and Asn62 with the nitrogen of the cyano group (CN) and the nitrogen of the diazoaryl group (N=N-Ar) with bond lengths of 3.30 and 3.42 Å, respectively. Further, the Zn^+2^ ion forming a metal complex with carbonyl of the ethyl ester group is shown in Figure 12.

Finally, according to the docking simulation results, it was found that the most active pyrazole derivatives **3a** and **5a** and pyrazolo[1,5-*a*]pyrimidine derivatives **6**, **9a**, and **10a** docked to the active sites of *h*CA-I and *h*CA-II with good energy scores (S), which supports their experimental activity. Additionally, the most active derivatives interacted with some amino acid residue as cocrystallized ligand and positive controls as Thr199 and/or His67 for *h*CA-I, as well as His64 and/or Asn62 for *h*CA-II. Furthermore, most of these derivatives could form a metal complex with the Zn^+2^ ion.

#### 2.3.3. Docking Simulation inside the Active Site of Alpha CA (PDB: 5HPJ)

**Figure 13:**
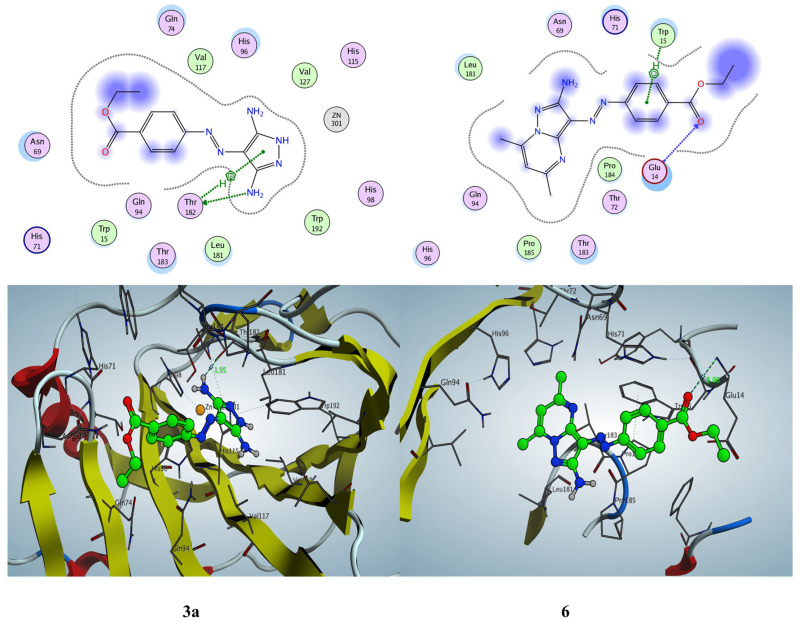
2D and 3D enzyme–ligand interaction of compounds **3a** and **6** inside the active site of alpha CA (PDB: 5HPJ).

Our work was extended to study the docking simulation of the most active derivatives, namely **3a**, **5a**, **6**, **9a**, and **10a**, inside the active site of Photobacterium profundum alpha-carbonic anhydrase (PDB: 5HPJ). The 3,5-diaminopyrazole derivative **3a** exhibited binding energy S = −5.09 Kcal/mol through the hydrogen bond side chain donor between Thr182 and the amino group of pyrazole core with a distance of 1.95 Å, as well as an arene–H interaction observed between the residue Thr182 and pyrazole nucleus. Similarly, pyrazolo[1,5-*a*]pyrimidine derivative **6** showed the lowest binding energy S = −5.66 Kcal/mol through one hydrogen bind backbone acceptor between the residue Glu14 and the oxygen of carbonyl of ethyl benzoate with a bond length of 3.85 Å. The phenyl group of ethyl benzoate exhibited an arene–H interaction with the amino acid Trp15; see Figure 13.

**Figure 14:**
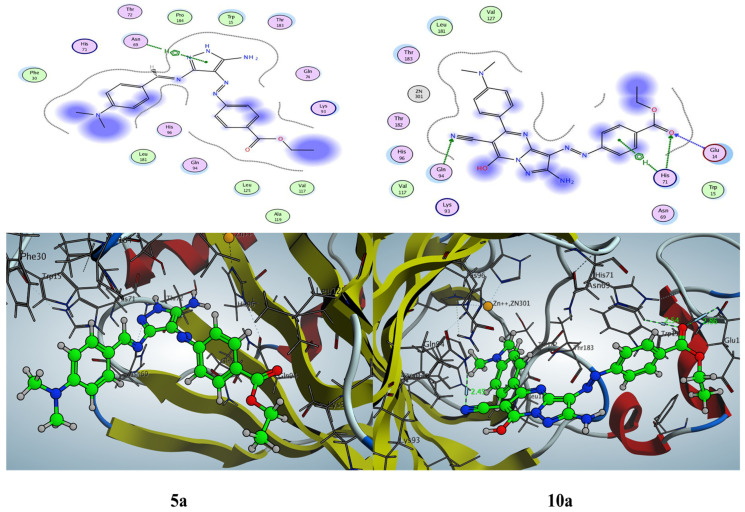
2D and 3D enzyme–ligand interaction of compounds **5a** and **10a** inside the active site of alpha CA (PDB: 5HPJ).

Furthermore, 7-hydroxy-pyrazolo[1,5-*a*]pyrimidine derivatives **10a** revealed binding energy S = −5.23 Kcal/mol with one hydrogen bond backbone acceptor between Glu19 and the oxygen of carbonyl of the benzoate group (2.86 Å) and two hydrogen bond side chain acceptors between His71 with the oxygen of the carbonyl group (2.54 Å) and between the residue Gln94 and the nitrogen of the cyano group (2.45 Å), as well as one arene–H interaction between His71 and the phenyl group of ethyl benzoate. Moreover, Schiff base pyrazole derivative **5a** demonstrated binding energy S = −5.28 Kcal/mol through one arene–H interaction between the Thr183 and pyrazole nucleus with a hydrophobic interaction appearing on the 4-ethoxyphenyl and *N*,*N*-dimethyl phenylamine group. Additionally, compound **9a** showed binding energy S = −5.31 Kcal/mol through one hydrogen bond side chain acceptor between Gln94 and the nitrogen of the cyano group with a bond length of 2.49 Å. The most active derivatives showed a hydrophobic interaction mainly on the ethyl benzoate group, pyrazolo[1,5-*a*]pyrimidine, the *N*,*N*-dimethylaminobenzylidene group, and some other groups, such as the methyl, cyano, hydroxy, and amino groups; see Figure 14. (All docking figures are in the Supplementary Materials file).

#### 2.3.4. Docking Simulation inside the Active Site of Beta CA (PDB: 1I6P)

**Figure 15:**
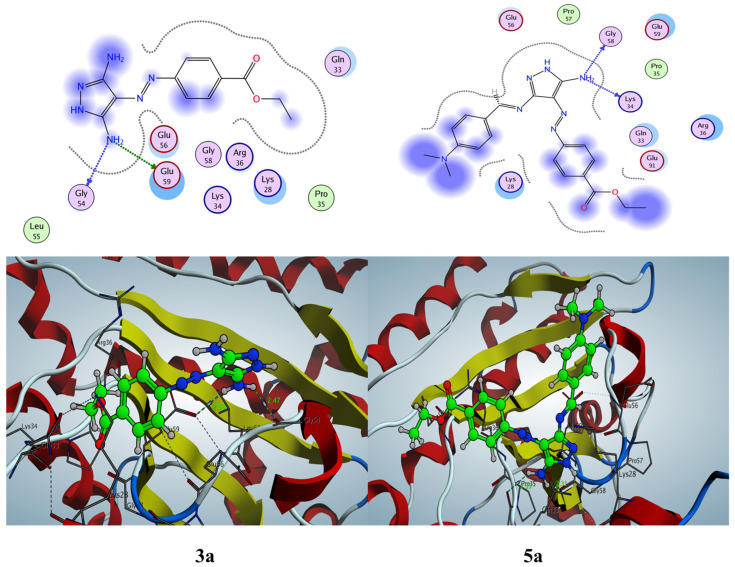
2D and 3D enzyme–ligand interaction of compounds **3a** and **5a** inside the active site of beta CA (PDB: 1I6P).

The interaction between the most active derivatives and the beta carbonic anhydrase enzyme was determined by using the docking simulation in the active site of the crystal structure of *E. coli* beta carbonic anhydrase (ECCA) (PDB: 1I6P) that was obtained from the protein data bank. The docking study illustrated the binding energy, interacting group, and bond length between the ligand and the protein. The 3,5-diaminopyrazole derivative **3a** showed binding energy S = −4.69 Kcal/mol through one hydrogen bond backbone donor and one hydrogen bond side chain donor between Gly54 and Gly59 with the amino group in the pyrazole moiety with bond lengths of 2.47 and 2.58 Å, respectively. Moreover, the Schiff base pyrazole derivative **5a** revealed binding energy S = −4.53 Kcal/mol with two hydrogen bond backbone donors between the amino group of pyrazole and the residues Gly58 and Lys34 with bond lengths of 2.31 and 2.35 Å, respectively (Figure 15).

Furthermore, 2,7-diaminopyrazolo[1,5-*a*]pyrimidine derivative **9a** exhibited the lowest binding energy S = −5.03 Kcal/mol through one hydrogen bond side chain acceptor Lys28 and the nitrogen of the cyano group with a bond length of 3.37 Å. Additionally, the blue regions in the 2D structure that localized over the two amino groups, the phenyl and ethyl of the ethyl benzoate moiety and pyrazolopyrimidine, represented a hydrophobic interaction. Similarly, the 7-hydroxy-pyrazolo[1,5-*a*]pyrimidine derivatives **10a** displayed the second lowest binding energy S = −4.99 Kcal/mol through two hydrogen bonds between Lys34 and Arg36 with the hydroxy group and the nitrogen of the cyano group at C6 of the pyrazolo[1,5-*a*]pyrimidine derivative with a bond length of 2.08 and 3.77 Å, respectively.

Finally, compound **6** demonstrated binding energy S = −4.71 Kcal/mol through one hydrogen bond side chain acceptor between the residue Lys28 and the nitrogen of the diazoaryl group (-N=N-), as well an arene–H interaction between Lys28 and the phenyl of the ethyl benzoate moiety. (All docking figures are in the Supplementary Materials file).

### 2.4. In Silico ADMET Prediction

Computer-based applications were used to evaluate the physicochemical properties, drug-likeness prediction, and medicinal chemistry of the most active molecules. Our study identified a drug-like molecule according to its molecular structure, physicochemical properties, water solubility, and lipophilicity. First, the SwissADME web tool (http://swissadme.ch/index.php) (accessed on 5 November 2022)was used for this purpose, as described previously [78]. The most active derivatives obeyed Lipinski’s rule of five with no violation, while three compounds **3a**, **5a**, and **6** were considered for the Veber rule. Additionally, the other compounds, **9a**, **10a**, and acetazolamide, did not follow the Veber rule, because the TPSA > 140. A drug molecule must be soluble to be bioavailable. The tested derivatives showed Log S (calculated with the ESOL model) from −5.27 to −2.58 compared with acetazolamide Log S = −1.14 with soluble to moderately soluble properties (Table 6).

Furthermore, the most potent derivatives showed one PAIN alarm thanks to the diazo group (N=N), easy synthetic accessibility (268–3.91), and a bioavailability score of 0.55, except for compound **9a** (0.56). The 3,5-diaminopyrazole **3a** and pyrazolo[1,5-*a*]pyrimidine derivative **6** revealed lead-likeness with complete agreement with all criteria (250 ≤ M.Wt ≤ 350, Xlogp ≤ 3.5, and NRB ≤ 7), while compounds **9a** and **10a** observed non-lead-likeness with two violations (MW > 350, XLOGP3 > 3.5), and compound **5a** exhibited three violations (MW > 350, NRB > 7, XLOGP3 > 3.5); see Table 6.

During the drug development process, it is essential to study the toxicity of candidate molecules in order to evaluate their detrimental effects on human health and the environment [79,80]. The most active derivatives demonstrated inactive probability to hepatotoxic, immunotoxin, cytotoxic, and phosphoprotein p53. However, most of the tested compounds and acetazolamide exhibited possibility carcinogenic activity, except for pyrazolo[1,5-*a*]pyrimidine derivative **10a**. The active derivatives belong to different toxicity classes, from III to V, with LD_50_ = 500–5000 mg/kg compared with acetazolamide (LD_50_ = 4300 mg/kg and class V). Moreover, the aryl hydrocarbon receptor (AhR) is a receptor that responds to environmental chemicals, such as homeostasis, detoxification, or immune responses [81]. Most of the tested derivatives and acetazolamide showed inactivity properties to AhR, except for pyrazolo[1,5-*a*]pyrimidine derivative **6**, which displayed activity properties with a possibility of 0.64 (Table 7).

Finally, it can be concluded that the most active pyrazole and pyrazolo[1,5-*a*]pyrimidine derivatives, namely **3a**, **5a**, **6**, **9a**, and **10a**, showed satisfactory ADMET profiles, which opened the door for the development of innovative anti-MDR bacterial therapeutics.

## 3. Materials and Methods

### 3.1. Chemistry Materials and Equipment

All chemical and solvent were obtained from Sigma-Aldrich (St. Louis, MO, USA) and were used without further purification. Melting points (MPs) of all the newly designed compounds were recorded on a digital Gallen Kamp MFB-595 instrument using open capillaries. IR spectra were determined using the KBr disk technique on a Shimadzu 440 spectrophotometer within the range of 400–4000 cm^−1^. Additionally, ^1^H/^13^C in the NMR spectra were obtained on a JOEL spectrometer 400/101 MHz; DMSO-*d*_6_ were used as solvents; and chemical shifts were measured in *δ* ppm, relative to TMS as an internal standard (=0.0 ppm). The data were presented as follows: chemical shift, multiplicity (s = singlet, d = doublet, t = triplet, q = quartet, m = multiplet, br = broad, and app = apparent), coupling constant (*J*) in Hertz (Hz), and integration. Elemental analyses were performed at the Micro Analytical Unit, Cairo University, Cairo, Egypt. The mass spectra were performed at the Regional Center for Mycology and Biotechnology, Al-Azhar University, and recorded on Thermo Scientific ISQLT mass spectrometer.

- **Synthesis of ethyl 4-((dicyanomethyl)diazenyl)benzoate (2)**

As mentioned in the literature method with some modification [82,83], using an ice-cooled solution of ethyl *p*-amino benzoate **1** (0.01 mol) in hydrochloric acid (2.5 mL) and distilled water (5 mL), a solution of sodium nitrite (0.013 mol) in distilled water (5 mL) was added. The cold diazotized solution was then added portion-wise to a well-stirred cold solution of malononitrile (0.01 mol) in 50% aqueous ethanol (10 mL) containing sodium acetate (0.01 mol). The reaction mixture was kept on ice for 2 h and then filtered. The precipitate was dried and crystallized from ethanol to obtain a yellow powder and M.p. = 178–180 °C.

- **Synthesis of ethyl 4-((3,5-diamino-1*H*-pyrazol-4-yl)diazenyl)benzoates (3a–c) and (4a,b)**

To a solution of compound **2** (0.01 mol) and hydrazine hydrate, phenylhydrazine, thiosemicarbazide, and cyanoacetohydrazide (0.01 mol) in ethanol (30 mL) were heated under reflux for 2–4 h. The solvent was removed under reduced pressure by a rotary evaporator. The resulting precipitate was filtered, dried, and washed with methanol to afford the products or recrystallized from the same solvent. Spectroscopic data of **3a**–**d** are mentioned below.

**Ethyl 4-((3,5-diamino-1*H*-pyrazol-4-yl)diazenyl)benzoate (3a)**

Yield 80%, M.p.= 242–244 °C, as deep yellow crystals; IR (KBr, cm^−1^) *ν* 3390, 3298, 3178 (2NH_2_, NH), 2978, 2939 (CH. aliph.), 1716 (C=O), 1612 (C=N), 1562 (N=N); ^1^H NMR (400 MHz, DMSO-*d*_6_) *δ* 1.29 (t, *J* = 5.6 Hz, 3H, CH_3_), 4.28 (q, *J* = 6.0 Hz, 2H, OCH_2_), 5.95 (s, 2H, NH_2_; exchangeable by D_2_O), 6.43 (s, 2H, NH_2_; exchangeable by D_2_O), 7.61 (d, *J* = 7.6 Hz, 2H), 7.92 (d, *J* = 8.0 Hz, 2H), 10.65 (s, 1H, NH; exchangeable by D_2_O); ^13^C NMR (101 MHz, DMSO-*d*_6_) *δ* 14.84 (CH_3_), 61.04 (OCH_2_), 116.39, 120.24, 121.82, 127.44, 130.30, 130.49 (Ar-Cs), 157.96 (C-NH_2_), 165.94 (C=O); MS (EI, 70 eV): *m*/*z* (%) = 274.96 (M^+^) (14.76%), 64.86 (100%); Anal. Calcd. for C_12_H_14_N_6_O_2_ (274.28): Calcd.: % C, 52.55; % H, 5.15; % N, 30.64. Found: C, 52.25; H, 5.30; % N, 30.79 %.

**Ethyl 4-((3,5-diamino-1-phenyl-1*H*-pyrazol-4-yl)diazenyl)benzoate (3b)**

Yield 84%, M.p.= 144–146 °C, as light yellow crystals; IR (KBr, cm^−1^) *ν* 3418, 3355, 3281, 3198 (2NH_2_), 2980, 2934 (CH. aliph.), 1698 (C=O), 1618 (C=N), 1560 (N=N); ^1^H NMR (400 MHz, DMSO-*d*_6_) *δ* 1.31 (t, *J* = 5.6 Hz, 3H, CH_3_), 4.30 (q, *J* = 5.7 Hz, 2H, OCH_2_), 6.32 (s, 2H, NH_2_; exchangeable by D_2_O), 6.96 (s, 2H, NH_2_; exchangeable by D_2_O), 7.30 (t, *J* = 5.8 Hz, 1H), 7.47 (t, *J* = 6.4 Hz, 2H), 7.54 (d, *J* = 6.8 Hz, 2H), 7.84 (d, *J* = 7.2 Hz, 2H), 7.97(d, *J* = 6.4 Hz, 2H); ^13^C NMR (101 MHz, DMSO-*d*_6_) *δ* 14.36 (CH_3_), 60.75 (OCH_2_), 120.82, 120.95, 122.75, 122.96, 126.89, 127.79, 129.59, 129.84, 130.49 (Ar-Cs), 157.69 (2C-NH_2_), 166.11 (C=O); MS (EI, 70 eV): *m*/*z* (%) = 350.34 (M^+^) (22.66%), 152.15 (100%); Anal. Calcd. for C_18_H_18_N_6_O_2_ (350.38): Calcd.: % C, 61.70; % H, 5.18; % N, 23.99. Found: C, 61.55; % H, 5.48; N, 24.06 %.

**Ethyl 4-((3,5-diamino-1-carbamothioyl-1*H*-pyrazol-4-yl)diazenyl)benzoate (3c)**

Yield 79%, M.p.= 280–282 °C, as light orange crystals; IR (KBr, cm^−1^) *ν* 3463, 3265, 3132 (3NH_2_), 2979, 2934 (CH-aliph.), 1710 (C=O), 1601 (C=N), 1542 (N=N), 857 (C=S); ^1^H NMR (400 MHz, DMSO-*d*_6_) *δ* 1.30 (t, *J* = 6.8 Hz, 3H, CH_3_), 4.29 (q, *J* = 7.0 Hz, 2H, OCH_2_), 5.74 (s, 2H, NH_2_; exchangeable by D_2_O), 6.32 (s, 2H, NH_2_; exchangeable by D_2_O), 6.95 (s, 2H, NH_2_; exchangeable by D_2_O), 7.54 (d, *J* = 7.4 Hz, 2H), 7.97 (d, *J* = 6.8 Hz, 2H); ^13^C NMR (101 MHz, DMSO-*d*_6_) *δ* 14.36 (CH_3_), 60.75 (OCH_2_), 117.25, 121.20, 122.57, 127.96, 129.73, 130.07, 130.81 (Ar-Cs), 157.69 (C-NH_2_), 166.11 (2C=O), 173.20 (C=S); Anal. Calcd. for C_13_H_15_N_7_O_2_S (333.37): Found: C, 46.84; % H, 4.54; % N, 29.41. Found: C, 46.95; % H, 4.49; N, 29.35 %.

**Ethyl 4-((3,5-diamino-1-(2-cyano-1-hydroxyvinyl)-1*H*-pyrazol-4-yl)diazenyl)benzoate (4a)**

Yield 85%, M.p.= 290–292 °C, as orange crystals; IR (KBr, cm^−1^) *ν* 3468, 3410, 3264, 3129 (2NH_2_, OH), 2980, 2934 (CH, aliph.), 2229 (CN), 1712 (C=O), 1628 (C=N), 1599 (N=N); ^1^H NMR (400 MHz, DMSO-*d*_6_) *δ* 1.32 (t, *J* = 6.4 Hz, 3H, CH_3_), 4.33 (q, *J* = 6.8 Hz, 2H, OCH_2_), 6.31 (s, 4H, 2NH_2_; exchangeable by D_2_O), 7.05 (s, 1H, methine-H), 7.75 (d, *J* = 6.0 Hz, 1H), 8.00 (d, *J* = 8.2 Hz, 1H), 8.03 (d, *J* = 6.0 Hz, 1H), 8.11 (d, *J* = 6.2 Hz, 1H), 8.20 (s, 1H, br-OH; exchangeable by D_2_O); ^13^C NMR (101 MHz, DMSO-*d*_6_) *δ* 14.22 (CH_3_), 50.25 (=CHCN), 60.76 (OCH_2_), 109.12, 116.79 (CN), 120.64, 121.80, 129.04, 130.08, 130.30, 140.13 (Ar-Cs), 150.42 (2C-NH_2_), 156.43 (C-OH), 165.52 (C=O); MS (EI, 70 eV): *m*/*z* (%) = 341.18 (M^+^) (12.11%), 249.79 (100%); Anal. Calcd. for C_15_H_15_N_7_O_3_ (341.33): Calcd.: C, 52.78; H, 4.43; N, 28.73. Found: C, 52.33; H, 4.62; N, 28.99 %.

- **Synthesis of Schiff bases derivatives (5a,b)**

To a mixture of 3,5-diaminopyrazole derivative **3a** (0.01 mol) and the requisite aldehydes, 4-(dimethylamino)benzaldehyde and 4-methoxybenzaldehyde (0.01 mol) in ethanol (20 mL) was catalyzed with glacial acetic acid (2 mL). The reaction mixture was heated under reflux for 4–6 h. The reaction mixture was cooled, and the solid product was collected and recrystallized from AcOH. Spectroscopic data of **5a**,**b** are mentioned below.

**Ethyl 4-((5-amino-3-((4-(dimethylamino)benzylidene)amino)-1*H*-pyrazol-4-yl)diazenyl)benzoate** **(5a)**

Yield 88%, M.p.= 250–252 °C, as red crystals; IR (KBr, cm^−1^) *ν* 3394, 3282, 3203 (NH_2_, NH), 2980, 2923 (CH. aliph.), 1715 (C=O), 1614 (C=N), 1562 (N=N); ^1^H NMR (400 MHz, DMSO-*d*_6_) *δ* 1.29 (3H, t, *J* = 5.6 Hz, CH_3_), 3.33 (6H, s, -N(CH_3_)_2_), 4.28 (2H, q, *J* = 5.6 Hz, OCH_2_), 5.95 (s, 2H, NH_2_; exchangeable with D_2_O), 7.41 (2H, d, *J* = 8.4 Hz, Ar-H), 7.61 (2H, d, *J* = 6.8 Hz, Ar-H), 7.75 (2H, d, *J* = 5.2 Hz, Ar-H), 7.92 (2H, d, *J* = 6.8 Hz, Ar-H), 8.99 (1H, s, methylinic-CH), 10.61 (1H, s, NH; exchangeable by D_2_O); ^13^C NMR (101 MHz, DMSO-*d*_6_) *δ* 14.50 (CH_3_), 45.28 (N(CH_3_)_2_), 61.04 (OCH_2_), 114.91, 118.47, 120.54, 121.33, 129.31, 129.60, 130.88, 131.37, 132.66 (Ar-Cs), 153.96 (C-N(Me)_2_), 157.18 (C-NH_2_), 162.60 (CH=N), 165.95 (C=O); MS (EI, 70 eV): *m*/*z* (%) = 405.69 (M^+^) (41.85%), 183.91 (100%); Anal. Calcd for C_21_H_23_N_7_O_2_ (405.46): Calcd.: C, 62.21; H, 5.72; N, 24.18. Found: C, 62.45; H, 5.58; N, 24.28 %.

**Ethyl-4-((5-amino-3-((4-methoxybenzylidene)amino)-1*H*-pyrazol-4-yl)diazenyl)benzoate (5b)**

Yield 86%; as pale red crystals; M.p.= 210–212 °C; IR (KBr, cm^−1^): 3395, 3263, 3176 (NH_2_, NH), 2978, 2935 (CH-aliph.), 1707 (C=O), 1623 (C=N), 1595 (N=N); ^1^H NMR (400 MHz, DMSO-*d*_6_) *δ* 1.30 (3H, t, *J* = 5.6 Hz, CH_3_), 3.81 (3H, s, OCH_3_), 4.29 (2H, q, *J* = 5.2 Hz, OCH_2_), 7.07 (2H, d, *J* = 6.8 Hz, Ar-H), 7.24 (br-s, 2H, NH_2_; exchangeable with D_2_O), 7.75 (2H, d, *J* = 6.8 Hz, Ar-H), 7.93 (2H, d, *J* = 6.8 Hz, Ar-H), 8.00 (2H, d, *J* = 6.8 Hz, Ar-H), 9.09 (1H, s, methylinic-CH), 9.82 (1H, s, NH; exchangeable by D_2_O); ^13^C NMR (101 MHz, DMSO-*d*_6_) *δ* 14.64 (CH_3_), 55.91 (OCH_3_), 61.33 (OCH_2_), 114.41, 116.39, 118.76, 127.04, 128.31, 129.30, 130.57, 131.17, 132.16 (Ar-Cs), 156.88 (C-NH_2_), 157.94 (CH=N), 162.79 (C-OCH_3_), 165.94 (C=O); Anal. Calcd. for C_20_H_20_N_6_O_3_ (392.41): Calcd.: % C, 61.22; % H, 5.14; % N, 21.42. Found: % C, 61.45; % H, 5.02; % N, 21.32.

- **Synthesis of ethyl-4-((2-amino-5,7-dimethylpyrazolo[1,5-*a*]pyrimidin-3-yl)diazenyl)benzoate (6)**

A solution of 3,5-diaminopyrazole derivative **3a** (0.01 mol) in acetic acid (15 mL) was treated with acetylacetone (0.01 mol). The reaction mixture was heated under reflux for 4 h and was cooled, and the solvent was removed under reduced pressure by a rotary evaporator. The solid product was collected and recrystallized from EtOH/AcOH. Spectroscopic data of pyrazolo[1,5-*a*]pyrimidine derivative **6** are mentioned below.

Pale yellow powder; yield 80%; M.p.=200–202 °C; IR (KBr, cm^−1^): 3402, 3285 (NH_2_), 2978, 2928, 2903 (CH-aliph.), 1713 (C=O), 1626 (C=N), 1566 (N=N); ^1^H NMR (400 MHz, DMSO-*d*_6_) *δ* 1.31 (3H, t, *J* = 5.8 Hz, CH_3_), 1.87 (3H, s, CH_3_), 2.52 (3H, s, CH_3_), 4.30 (2H, q, *J* = 5.8 Hz, OCH_2_), 6.92 (1H, s, 4H-pyrimidine), 7.30 (s, 2H, NH_2_; exchangeable with D_2_O), 7.81 (2H, d, *J* = 6.8 Hz, Ar-H), 7.99 (2H, d, *J* = 6.8 Hz, Ar-H); ^13^C NMR (101 MHz, DMSO-*d*_6_) *δ* 14.64 (CH_3_), 17.06 (CH_3_), 24.32 (CH_3_), 61.07 (OCH_2_), 111.13, 116.19 (CH-pyrimidine), 121.39, 128.86, 129.08, 130.73, 146.39, 147.66 (Ar-Cs), 156.63 (C-NH_2_), 160.91(CH_3_-C=N), 166.12 (C=O); MS (EI, 70 eV): *m*/*z* (%) = 338.95 (M^+^) (39.72%), 328.60 (100%); Anal. Calcd. for C_17_H_18_N_6_O_2_ (338.37): Calcd.: % C, 60.34; % H, 5.36; % N, 24.84. Found: % C, 59.85; % H, 5.70; % N, 24.99.

- **Synthesis of ethyl 4-((2-amino-5-methyl-7-oxo-6,7-dihydropyrazolo[1,5-*a*]pyrimidin-3-yl)diazenyl)benzoate (7a)**

A solution of 3,5-diaminopyrazole derivative **3a** (0.01 mol) in acetic acid (15 mL) was treated with ethyl acetoacetate (0.01 mol). The reaction mixture was heated under reflux for 4 h and was cooled, and the solvent was removed under reduced pressure by a rotary evaporator. The solid product was collected and recrystallized from EtOH/AcOH. Spectroscopic data of pyrazolopyrimidine derivatives **7a** are mentioned below.

White powder; yield 78%; M.p.= 296–298 °C; IR (KBr, cm^−1^): 3415, 3248, 3178 (OH, NH_2_), 2978, 2935 (CH-aliph.), 1687 (C=O), 1633 (C=N), 1582 (N=N); ^1^H NMR (400 MHz, DMSO-*d*_6_) *δ* 1.32 (3H, t, *J* = 6.6 Hz, CH_3_), 2.32 (3H, s, CH_3_), 3.42 (2H, s, CH_2_), 4.30 (2H, q, OCH_2_), 6.74 (s, 2H, NH_2_; exchangeable with D_2_O), 7.90 (2H, d, *J* = 6.4 Hz, Ar-H), 8.02 (2H, d, *J* = 5.2 Hz, Ar-H); ^13^C NMR (101 MHz, DMSO-*d*_6_) *δ* 14.16 (CH_3_), 25.78 (CH_3_), 31.40 (CH_2_-pyrimidine), 60.55 (OCH_2_), 111.77, 116.40, 121.80, 129.10, 131.17, 145.85, 146.84, 152.25 (Ar-Cs), 156.69 (C-NH_2_), 160.73 (CH_3_-C=N), 165.65, 167.43 (2C=O); MS (EI, 70 eV): *m*/*z* (%) = 340.95 (M^+^) (24.33%), 232.87 (100%); Anal. Calcd. for C_16_H_16_N_6_O_3_ (340.34): Calcd.: % C, 56.47; % H, 4.74; % N, 24.69. Found: % C, 56.72; % H, 4.45; % N, 24.43.

- **Synthesis of ethyl 4-((2-amino-7-aryl-5-phenylpyrazolo[1,5-*a*]pyrimidin-3-yl)diazenyl)benzoate (8a–c)**

To a suspension solution of 3,5-diaminopyrazole derivative **3a** (0.01 mol) in absolute ethanol (20 mL) and α, β-unsaturated ketones, namely (1-phenyl-3-(thiophen-2-yl)prop-2-en-1-one, 3-(furan-2-yl)-1-phenylprop-2-en-1-one, and 3-(4-chlorophenyl)-1-phenylprop-2-en-1-one) (0.01 mol), three drops of piperidine were added. The resulting mixture was heated under reflux for 4 h and was cooled, and the solvent was removed under reduced pressure by a rotary evaporator. The solid product was collected and recrystallized from EtOH to yield the desired pure product. Spectroscopic data of pyrazolo[1,5-*a*]pyrimidine derivatives **8a**–**c** are mentioned below.

**Ethyl-4-((2-amino-5-phenyl-7-(thiophen-2-yl)pyrazolo[1,5-*a*]pyrimidin-3-yl)diazenyl)benzoate (8a)**

Brown powder; yield 85%; M.p.=200–202 °C; IR (KBr, cm^−1^): 3438, 3266 (NH_2_), 2981 (CH-aliph.), 1709 (C=O), 1613 (C=N), 1566 (N=N); ^1^H NMR (400 MHz, DMSO-*d*_6_) *δ* 1.32 (3H, t, *J* = 4.8 Hz, CH_3_), 4.31 (2H, q, *J* = 4.9 Hz, OCH_2_), 6.21 (1H, t, *J* = 6.2 Hz, Ar-H), 6.28 (2H, br-s, NH_2_; exchangeable with D_2_O), 6.99 (1H, d, *J* = 6.6 Hz, Ar-H), 7.15 (1H, s, CH-pyrimidine), 7.45–7.61 (5H, m, Ar-H), 7.91 (2H, d, *J* = 4.0 Hz, Ar-H), 7.96 (2H, d, *J* = 3.2 Hz, Ar-H), 8.19 (1H, d, *J* = 8.0 Hz, Ar-H); ^13^C NMR (101 MHz, DMSO-*d*_6_) *δ* 14.64 (CH_3_), 61.01 (OCH_2_), 98.97, 102.57, 121.48, 126.68, 127.05, 127.80, 128.32, 129.41, 129.73, 130.58, 131.01, 134.73, 135.84, 136.92, 137.24, 139.43, 146.94 (Ar-Cs), 153.17 (C-NH_2_), 156.77 (C=N), 166.08 (C=O); MS (EI, 70 eV): *m*/*z* (%) = 468.28 (M^+^) (18.25%), 118.12 (100%); Anal. Calcd. for C_25_H_20_N_6_O_2_S (468.14): Calcd.: % C, 64.09; % H, 4.30; % N, 17.94. Found: % C, 64.30; % H, 4.21; % N, 17.82.

**Ethyl 4-((2-amino-7-(furan-2-yl)-5-phenylpyrazolo[1,5-*a*]pyrimidin-3-yl)diazenyl)benzoate (8b)**

Deep red powder; yield 82%; M.p.= 212–214 °C; IR (KBr, cm^−1^): 3427, 3272 (NH_2_), 2923 (CH-aliph.), 1709 (C=O), 1595 (C=N), 1568 (N=N); ^1^H NMR (400 MHz, DMSO-*d*_6_) *δ* 1.32 (3H, t, *J* = 5.2 Hz, CH_3_), 4.31 (2H, q, *J* = 5.8 Hz, OCH_2_), 6.27 (2H, br-s, NH_2_; exchangeable with D_2_O), 6.45 (1H, t, *J* = 5.8 Hz, Ar-H), 7.47 (2H, t, *J* = 5.4 Hz, Ar-H), 7.61 (2H, d, *J* = 5.4 Hz, Ar-H), 7.74 (1H, t, *J* = 6.4 Hz, Ar-H), 7.87 (1H, s, CH-pyrimidine), 7.92–7.98 (4H, m, Ar-H), 8.06 (1H, d, *J* = 5.6 Hz, Ar-H), 8.22 (1H, d, *J* = 5.6 Hz, Ar-H); ^13^C NMR (101 MHz, DMSO-*d*_6_) *δ* 14.64 (CH_3_), 61.07 (OCH_2_), 108.51, 111.12, 113.94, 115.38, 120.61, 121.26, 121.49, 126.70, 127.70, 128.50, 129.15, 130.52, 130.74, 131.32, 134.81, 137.20, 143.42, 148.13, 153.78 (Ar-Cs), 157.62 (C-NH_2_), 160.21 (C=N), 166.24 (C=O); Anal. Calcd. for C_25_H_20_N_6_O_3_ (452.47): Calcd.: % C, 66.36; % H, 4.46; % N, 18.57. Found: % C, 66.57; % H, 4.36; % N, 18.46.

**Ethyl-4-((2-amino-7-(4-chlorophenyl)-5-phenylpyrazolo[1,5-*a*]pyrimidin-3-yl)diazenyl)benzoate (8c)**

Red brown powder; yield 87%; M.p.= 208–2010 °C; IR (KBr, cm^−1^): 3417, 3270 (NH_2_), 2982, 2925 (CH-aliph.), 1704 (C=O), 1618 (C=N), 1567 (N=N); ^1^H NMR (400 MHz, DMSO-*d*_6_) *δ* 1.34 (3H, t, *J* = 5.6 Hz, CH_3_), 4.33 (2H, q, *J* = 4.5 Hz, OCH_2_), 6.28 (2H, br.s, NH_2_; exchangeable with D_2_O), 7.36 (2H, d, *J* = 6.4 Hz, Ar-H), 7.44–7.51 (5H, m, Ar-H), 7.63 (2H, d, *J* = 5.2 Hz, Ar-H), 7.72 (1H, s, CH-pyrimidine), 7.91 (2H, d, *J* = 6.0 Hz, Ar-H), 8.01 (2H, d, *J* = 6.8 Hz, Ar-H); ^13^C NMR (101 MHz, DMSO-*d*_6_) *δ* 14.56 (CH_3_), 60.58 (OCH_2_), 108.33, 109.30, 111.45, 115.14, 115.91, 120.00, 125.23, 129.45, 129.88, 130.43, 132.17, 132.79 134.88, 136.40, 137.24, 140.51, 142.46, 146.91, 149.20 (Ar-Cs), 155.80 (C-NH_2_), 158.72 (C=N), 167.06 (C=O); Anal. Calcd. for C_27_H_21_ClN_6_O_2_ (496.96): Calcd.: % C, 65.26; % H, 4.26; % N, 16.91. Found: % C, 65.01; % H, 4.40; % N, 17.01.

- **Synthesis of ethyl-4-((2,7-diamino-6-cyano-5-(*p*-substituted)pyrazolo[1,5-*a*]pyrimidin-3-yl)diazenyl)benzoate (9a–c)**

To a suspension solution of 3,5-diaminopyrazole derivative **3a** (0.01 mol) in absolute ethanol (20 mL) and arylidene malononitrile derivatives (0.01 mol), five drops of piperidine were added. The resulting mixture was heated under reflux for 6 h and was cooled, and the solvent was removed under reduced pressure by rotary evaporator. The solid product was collected and recrystallized from EtOH to yield the desired pure product. Spectroscopic data of pyrazolopyrimidine derivatives **9a**–**c** are mentioned below.

**Ethyl-4-((2,7-diamino-6-cyano-5-(4-(dimethylamino)phenyl)pyrazolo[1,5-*a*]pyrimidin-3-yl)diazenyl)benzoate (9a)**

Brown powder; yield 80%; M.p.= 180–182 °C; IR (KBr, cm^−1^): 3396, 3304 (2NH_2_), 2926 (CH-aliph.), 2211 (CN), 1714 (C=O), 1615 (C=N), 1565 (N=N); ^1^H NMR (400 MHz, DMSO-*d*_6_) *δ* 1.31 (3H, t, *J* = 5.6 Hz, CH_3_), 3.07 (6H, s, N(CH_3_)_2_, 4.28 (2H, q, *J* = 5.4 Hz, OCH_2_), 6.37 (2H, br-s, NH_2_; exchangeable with D_2_O), 6.84 (2H, s, NH_2_; exchangeable with D_2_O), 7.73 (2H, d, *J* = 6.4 Hz, Ar-H), 7.82 (2H, d, *J* = 6.4 Hz, Ar-H), 7.93 (2H, d, *J* = 6.4 Hz, Ar-H), 8.02 (2H, d, *J* = 6.4 Hz, Ar-H); ^13^C NMR (101 MHz, DMSO-*d*_6_) *δ* 14.76 (CH_3_), 42.13 (Nme_2_), 61.14 (OCH_2_), 111.96, 112.56, 115.32 (CN), 116.11, 116.60, 117.98, 119.16, 120.77, 127.34, 130.09, 130.39, 133.94 (Ar-Cs), 154.82 (C-NH_2_), 156.98 (N-C-NH_2_), 159.25 (C=N), 166.34 (C=O); MS (EI, 70 eV): *m*/*z* (%) = 469.19 (M^+^) (36.86%), 420.10 (100%); Anal. Calcd. for C_24_H_23_N_9_O_2_ (469.51): Calcd.: % C, 61.40; % H, 4.94; % N, 26.85. Found: % C, 61.05; % H, 5.10; % N, 26.99.

**Ethyl-4-((2,7-diamino-6-cyano-5-(4-methoxyphenyl)pyrazolo[1,5-*a*]pyrimidin-3-yl)diazenyl)benzoate** **(9b)**

Red powder; yield 75%; M.p.= 210–212 °C; IR (KBr, cm^−1^): 3434, 3303, 3232, 3171 (2NH_2_), 2979, 2930 (CH-aliph.), 2215 (CN), 1705 (C=O), 1616 (C=N), 1593 (N=N); ^1^H NMR (400 MHz, DMSO-*d*_6_) *δ*/ppm 1.29 (3H, t, *J* = 5.2 Hz, CH_3_), 3.83 (3H, s, OCH_3_), 4.27 (2H, q, OCH_2_), 6.04 (2H, s, NH_2_; exchangeable with D_2_O), 6.52 (2H, s, NH_2_; exchangeable with D_2_O), 7.13 (2H, d, *J* = 8.0 Hz, Ar-H), 7.73 (2H, d, *J* = 8.0 Hz, Ar-H), 7.93 (2H, d, *J* = 7.6 Hz, Ar-H), 8.07 (2H, d, *J* = 7.6 Hz, Ar-H); ^13^C NMR (101 MHz, DMSO-*d*_6_) *δ* 14.45 (CH_3_), 56.80 (-Ome), 60.65 (OCH_2_), 114.43, 115.32 (CN), 116.60, 120.76, 121.03, 123.79, 127.34, 128.42, 130.39, 132.06, 133.64 (Ar-Cs), 151.57 (C-NH_2_), 155.10 (N-C-NH_2_), 157.87 (C=N), 162.80 (C-Ome) 166.05 (C=O); Anal. Calcd. for C_23_H_20_N_8_O_3_ (456.47): Calcd.: % C, 60.52; % H, 4.42; % N, 24.55. Found: % C, 60.32; % H, 4.53; % N, 24.54.

**Ethyl-4-((2,7-diamino-5-(4-chlorophenyl)-6-cyanopyrazolo[1,5-*a*]pyrimidin-3-yl)diazenyl)benzoate** **(9c)**

Gray powder; yield 75%; M.p.= 288–290 °C; IR (KBr, cm^−1^): 3418, 3330 (2NH_2_), 2927 (CH-aliph.), 2219 (CN), 1711 (C=O), 1617 (C=N), 1589 (N=N); ^1^H NMR (400 MHz, DMSO-*d*_6_) *δ* 1.32 (3H, t, *J* = 5.2 Hz, CH_3_), 4.32 (2H, q, *J* = 5.2 Hz, OCH_2_), 6.09 (2H, br-s, NH_2_, exchangeable with D_2_O), 6.47 (2H, s, NH_2_, exchangeable with D_2_O), 7.21 (2H, d, *J* = 6.6 Hz, Ar-H), 7.63 (2H, d, *J* = 5.6 Hz, Ar-H), 7.87 (2H, d, *J* = 8.0 Hz, Ar-H), 8.03 (2H, d, *J* = 8.0 Hz, Ar-H); ^13^C NMR (101 MHz, DMSO-*d*_6_) *δ* 14.45 (CH_3_), 60.94 (OCH_2_), 108.70, 112.31, 116.05, 116.64, 118.05, 119.15, 120.77, 123.28, 124.89, 127.22, 130.44, 132.83, 134.06 (Ar-Cs), 155.11 (C-NH_2_), 157.57 (N-C-NH_2_), 158.95 (C=N), 165.56 (C=O); Anal. Calcd. for C_22_H_17_ClN_8_O_2_ (460.88): Calcd.: % C, 57.33; % H, 3.72; % N, 24.31. Found: % C, 57.55; % H, 3.60; % N, 24.23.

- **Synthesis of ethyl-4-((2-amino-6-cyano-7-hydroxy-5-(*p*-substituted)pyrazolo[1,5-*a*]pyrimidin-3-yl)diazenyl)benzoate (10a,b)**

To a mixture of 3,5-diaminopyrazole derivative **3a** (0.01 mol) in absolute ethanol (20 mL) and ethyl cyanocinnamate derivatives (0.01 mol), five drops of piperidine were added. The resulting mixture was heated under reflux for 6 h and was cooled, and the solvent was removed under reduced pressure by rotary evaporator. The solid product was collected and recrystallized from EtOH to yield the desired pure product. Spectroscopic data of pyrazolopyrimidine derivatives **10a**,**b** are mentioned below.

**Ethyl-4-((2-amino-6-cyano-5-(4-(dimethylamino)phenyl)-7-hydroxypyrazolo[1,5-*a*]pyrimidin-3-yl)diazenyl)benzoate (10a)**

Brown powder; yield 90%; M.p.= 258–260 °C; IR (KBr, cm^−1^): 3394, 3300, 3208 (OH, NH_2_), 2980 (CH-aliph.), 2218 (CN), 1716 (C=O), 1615 (C=N), 1563 (N=N); ^1^H NMR (400 MHz, DMSO-*d*_6_) *δ*/ppm 1.31 (3H, t, *J* = 5.2 Hz, CH_3_), 3.01 (3H, s, Nme_2_), 4.30 (2H, q, *J* = 5.4 Hz, OCH_2_), 6.92 (2H, s, NH_2_; exchangeable with D_2_O), 7.62 (2H, d, *J* = 8.0 Hz, Ar-H), 7.67–7.70 (4H, m, Ar-H), 7.96 (2H, d, *J* = 8.0 Hz, Ar-H), 10.27 (1H, s, OH; exchangeable with D_2_O); ^13^C NMR (101 MHz, DMSO-*d*_6_) *δ* 14.76 (CH_3_), 44.09 (Nme_2_), 60.93 (OCH_2_), 113.19, 116.26 (CN), 117.41, 117.74, 121.01, 123.26, 127.46, 128.56, 130.12, 130.68, 131.96, 135.77 (Ar-Cs), 150.94 (N-C-NH_2_), 155.16 (C-Nme_2_), 157.81 (C=N), 160.05 (C-OH) 166.10 (C=O); MS (EI, 70 eV): *m*/*z* (%) = 470.47 (M^+^) (19.54%), 240.76 (100%); Anal. Calcd. for C_24_H_22_N_8_O_3_ (470.49): Calcd.: % C, 61.27; % H, 4.71; % N, 23.82. Found: % C, 61.65; % H, 4.49; % N, 23.62.

**Ethyl 4-((2-amino-5-(4-chlorophenyl)-6-cyano-7-hydroxypyrazolo[1,5-*a*]pyrimidin-3-yl)diazenyl)benzoate (10b)**

Red brown powder; yield 85%; M.p.= 270–272 °C; IR (KBr, cm^−1^): 3398, 3285, 3108 (OH, NH_2_), 2956, 2857 (CH-aliph.), 2213 (CN), 1702 (C=O), 1625 (C=N), 1595 (N=N); ^1^H NMR (400 MHz, DMSO-*d*_6_) *δ* 1.29 (3H, t, J = 5.6 Hz, CH_3_), 4.25 (2H, q, *J* = 5.8 Hz, OCH_2_), 6.29 (2H, s, NH_2_; exchangeable with D_2_O), 7.60 (2H, d, J = 6.8 Hz, Ar-H), 7.72 (2H, d, *J* = 6.8 Hz, Ar-H), 7.91 (2H, d, *J* = 6.8 Hz, Ar-H), 7.99 (2H, d, *J* = 6.8 Hz, Ar-H), 10.84 (1H, s, OH; exchangeable with D_2_O); ^13^C NMR (101 MHz, DMSO-*d*_6_) *δ* 14.77 (CH_3_), 61.01 (OCH_2_), 113.53, 113.73, 115.52 (CN), 116.23, 117.64, 120.73, 127.35, 127.68, 130.57, 131.46, 132.56, 135.78, 138.67, 155.84 (Ar-Cs), 157.12 (N-C-NH_2_), 159.77 (C-Nme_2_), 160.67 (C=N), 166.25 (C=O); Anal. Calcd. for C_22_H_16_ClN_7_O_3_ (461.87): Calcd.: % C, 57.21; % H, 3.44; % N, 21.23. Found: % C, 57.53; % H, 3.22; % N, 21.13.

### 3.2. Biological Activity

#### 3.2.1. Bacterial Strains, Microbiological Media, and Chemicals

Pyrazoles’ antibacterial activity was evaluated by using a panel of clinically isolated Gram-positive and Gram-negative bacteria (*Staphylococcus aureus* DH-432, *Streptococcus pyogenes* DH-3467, *Enterococcus faecalis* DH-5478, *Pseudomonus aeruginosa* DH-5698, *Acinetobacter baumannii* DH-243, and *Escherichia coli* DH-5987). The bacterial isolates were obtained from different clinical samples collected by the Central Laboratories, EL-Demerdash Hospital, Ain-Shams University, Cairo, Egypt. The CV026, a *Chromobacterium violaceum* mutant deficient in production of Nhexanoyl-L-homoserine lactone (C6HSL), which can produce purple pigmentation on an external application of C6HSL, was used as a reporter organism to test for QSI. Microbiological media, tryptic soy (TS) broth or agar, and sabouraud agar were obtained from Oxoid (Hampshire, UK). Dimethyl sulfoxide (DMSO) was used as a solvent for the synthesized derivatives, and the DMSO and other chemicals were of analytical grade.

#### 3.2.2. Screening of Synthesized Compounds for Antibacterial Activity

The synthesized compounds were initially screened for their in vitro antibacterial activity against the different Gram-positive and Gram-negative bacteria, tested using the agar well-diffusion assay [84]. The turbidity of the bacterial suspensions was established to the 0.5 McFarland turbidity standard. The activity was performed on TS agar plates. The bacteria were spread on the surface of the agar plates, and 10 mm wells were screwed into the agar plates. The assay was performed under aseptic conditions. An aliquot of 50 µL of each tested compound (10 mM) was added to the wells, and the plates were incubated overnight at 37 °C. Both Erythromycin and Amikacin antibiotic disks were used as positive control, while DMSO solvent was used as a negative control in this study. The diameters of inhibition zones were measured and recorded in mm. A comparative study of the synthesized compounds was determined. The experiment was performed in triplicates.

#### 3.2.3. Detection of Minimum Inhibitory Concentrations (MICs) and Minimum Bactericidal Concentrations (MBCs) of Pyrazoles

The broth microdilution method was taken as a basis to detect the MICs of pyrazole compounds against tested clinical isolates, according to the Clinical Laboratory and Standards Institute guidelines [85,86]. The broth cultures were used to prepare the bacterial inoculums, and the suspensions were adjusted to the standard 0.5 McFarland turbidity. Stock solutions of the synthesized pyrazoles were prepared in pure DMSO and further serially diluted with TS broth in the concentration range from 128 µg/mL to 0.0625 µg/mL. A well containing noninoculated TS broth, free of any antimicrobial agents, was also included to ensure the medium sterility, while Erythromycin and Amikacin served as positive controls. Bacterial growth was determined by adding 20 µL of 0.5% TTC (2,3,5-triphenyltetrazolium chloride) aqueous solution. After incubation of the plates for 24 h at 37 °C, the MIC was determined as the lowest concentration detected of the compounds that inhibited visible growth, indicated by the presence of red pellet in the bottom of the wells after the addition of TTC. All experiments were repeated three times. The bactericidal effect from each concentration of the pyrazole and pyrazolo[1,5-*a*]pyrimidine derivatives showing growth inhibition was further studied. For this study, a loopful of 5 µL was taken from each no-visible growth tube and streaked onto plates of TS agar. The plates were then incubated at 37 °C for 24 h, and the MBC was determined as the lowest concentration with no observed bacterial growth. The MBC/MIC ratio was recorded as if the ratio were ≤4, in which case the agent would be considered as bactericidal; otherwise the agent would be considered as bacteriostatic [87].

#### 3.2.4. Evaluation of Antibiofilm Activities of Pyrazole and Pyrazolo[1,5-*a*]pyrimidine Derivatives

- **Quantitative analysis of biofilm formation**

The biofilm-forming ability of the different clinical isolates was measured using the 96-well flat-bottomed microtiter polystyrene plates, as previously described [88]. Briefly, single colonies from the overnight bacterial growth were inoculated into 5 mL of sterile TSB, followed by incubation at 37 °C for 18 h, without shaking. The bacterial growth was adjusted to the 0.5 McFarland standard and then diluted by a 10-fold dilution method in TSB supplemented with 10% glucose. An aliquot of 200 µL of the diluted cultures was dispensed in three wells of the microtiter plate for each isolate and then incubated overnight at 37 °C. Brothy medium (TSB with 10% glucose) was used as negative control. After incubation, the planktonic cells were removed by washing the wells with PBS 3–5 times. Ethanol (95%) was used for the fixation of the adherent cells for 5 min, followed by discarding the ethanol; the wells were left to dry and for subsequent staining with 100 µL of 1% crystal violet. The wells were washed with sterile distilled water to rinse off the excess stain, and the plates were left to air dry. The optical density was measured at 570 nm. The biofilm formation of each isolate was classified as strong biofilm (OD_570_ ≥ 1), moderate biofilm (0.1 ≤ OD_570_ < 1), and nonbiofilm (OD_570_ < 0.1) producers.

- **Biofilm inhibition assay**

The microtiter plate method was used to evaluate the active pyrazoles’ inhibitory activities on the strong biofilm-forming bacterial strains. This method was carried out as described [89,90], with some modifications. The antibiofilm effects of tested pyrazoles are presented as a percentage change from untreated bacterial controls. Overnight cultures of micro-organisms in the TSB medium with 10% (wt/vol) glucose were prepared as 10^8^ CFU/mL, and then 100 µL was dispensed into each test well. Then, 100 µL of different concentrations of compounds, at their MICs, were dispensed into each well. The negative control contained only TSB, whereas the positive control contained cell cultures without compound addition. The supernatant was decanted and each well gently rinsed three times with 300 µL of sterile distilled water and discarded after incubation at 37 °C for 48 h. The plates were dried with air for 40 min. They were stained with 0.1% (wt/vol) crystal violet at room temperature for 30 min and washed three times with sterile distilled water. Afterward, the crystal violet was solubilized in 95% ethanol, and the absorbance was read in a microplate reader (MicroplatePhotometer and Multiskan FC) at 570 nm. This test was performed in triplicate, and the mean was taken as an average of three readings [89]. The percentage of biofilm inhibition was calculated according to Equation (1) [91]:

(1) Biofilm eradication %=OD positivecontrol−OD sample OD positive control×100

- **In situ visualization of biofilm formation inhibition**

The changes in the biofilm architecture of the strong biofilm-forming bacterial isolates upon exposure to the most active derivatives were examined through ascanning electron microscope (SEM) analysis. SEM was performed to visualize the biofilm in situ using glass cover slips [92,93]. Briefly, biofilms were grown for 24 h on glass cover slips placed on a 12-well plate with the pyrazoles treated and untreated cultures of the strong biofilm-forming bacterial isolates. Following the incubation of the plates overnight at 37 °C, the coverslips were washed with sterile distilled water three times and stained with crystal violet (1%) for 20 min. The formed biofilms on the coverslips were fixed by glutaraldehyde (2.5%) for 2 h. After washing with sterile distilled water, the coverslips were dehydrated by increasing ethanol concentrations (50%, 60%, 70%, 80%, 90%, and 100%) for 30 s. The samples were gold coated after critical point drying and examined under a scanning electron microscope (ZEISS EVO LS 15, UK) at a magnification of 7000× and a voltage of 25 kv.

- **Bioassay for quorum-sensing inhibition against biofilm-forming bacteria using CV026 as an indicator strain**

The anti-QS potential of the tested compounds was explored as described [94,95]. Briefly, an overnight bacterial suspension of the indicator organism *C. violaceum* CV026 was prepared in a concentration of 10^6^ CFU/mL, and an aliquot of 200 µL was seeded in 5 mm of Soft Top agar (1.3 g agar, 2.0 g tryptone, 1.0 g NaCl, and 200 mL deionized water), supplemented with 20 µL of 0.25 µg/mL C6-HSL (N-hexanoyl-L-homoserine lactone), added as an exogenous AHL source. Wells were cut in the center of the plate after incubation for 30 min and then filled with 50 µL of sub-MICs of each most-active pyrazole hybrid. DMSO was used as a negative control. The plates were incubated at 28 °C for 48 h. Inhibition of violacein production by the indicator strain around the well, indicated by the presence of a white circle surrounded by a purple halo, was considered as positive for QS interference. For further investigation to determine the anti-QS activity for the pyrazole against the biofilm-forming bacterial isolates, an in vitro screening was performed. This was carried out by inoculation of 200 µL from an O/N CV026 culture onto plates of Soft Top agar. Wells of 8 mm diameter were separately made and filled with 90 µL of each biofilm-forming bacterial isolate (10^6^ CFU/mL), plus 10 µL of each active pyrazole derivative (sub-MICs). Wells containing the bacterial isolates without treatment were used as controls. The signal diffusion zone diameter produced during the bacterial growth was measured in the presence of pyrazole and pyrazolo[1,5-*a*]pyrimidine derivatives and compared with the zone diameter of the violacein production by CV026.

(a) **Carbonic anhydrase I and II isoenzymes purification and inhibition assay**

The purification of both human cytosolic CA isoforms was carried out as previously described [96], using a simple one-step procedure of Sepharose-4B-L-tyrosine-sulfanilamide affinity chromatography. Sodium dodecyl sulfate-polyacrylamide gel electrophoresis (SDS-PAGE) was performed to verify the purity of the enzymes, and a single band was observed for each CA isoenzyme. The carbonic anhydrase isoenzymes’ activities were determined in accordance with the previous study [97]. According to the Bradford method, the protein quantity produced during the enzyme purification was spectrophotometrically determined at a wavelength of 595 nm [98]. Bovine serum albumin was used as the standard protein [99,100], and acetazolamide (AZA) was also used as a standard inhibitor for both CA-I and -II isoenzymes (*h*CA-I and -II). For the designation of the inhibition efficacy of each novel pyrazole and for the pyrazolo[1,5-*a*]pyrimidine derivatives on both *h*CA isoenzymes, an activity (%) graph was drawn. The IC_50_ values were obtained from activity (%) versus compounds plots. The *K_i_* values were calculated using three concentrations for each derivative.

(b) **Sterilization of the active pyrazolo-derivatives using gamma radiation**

The most active compounds were subjected to gamma radiation doses of 1.0, 3.0, 5.0, 7.0, 10.0, 15.0, and 20.0 kGy) using the ^60^C0-radioactive source located in the National Center of Research and Radiation Technology (NCRRT), Egyptian Atomic Energy Authority (EAEA), Egypt. The radiation dose was 1.0 kGy/h at the experiment time. To detect any physicochemical changes, the irradiated compounds were observed for any changes in the organoleptic properties (e.g., color, odor, and solubility). In addition, they were analyzed using the UV/VIS spectrophotometer at 570 nm. Furthermore, a microbiological sterility assay was carried out for the active compounds. Briefly, 0.05 g of each compound (irradiated and nonirradiated) was aseptically added to test tubes containing 4.5 mL sterile saline solution. The tubes were well mixed, and then aliquots of 100 µL were added to the surface of tryptic soya agar (for bacteria) and sabouraud agar (for fungi) plates. The TSA plates were incubated overnight at 35°C, while the SA plates were incubated for 48 h. The microbial colonies were counted and recorded as CFU/g. The nonirradiated samples were used as controls throughout the experiment [101,102].

### 3.3. Molecular Docking Simulations

The molecular docking simulation of the most active derivatives, namely 3a, 5a, 6, 9a, and 10a, was performed inside the active site of human carbonic anhydrase isoenzymes (*h*CA-I and -II) using Molecular Operating Environmental (MOE) 2014.0901, Chemical computing Group Inc., Montreal, Quebec, Canada. First, the structure of the most active derivatives was bulited in chembiodraw 2014 and exported to MOE, where the hydrogen atoms were added and minized using gradient 0.0001 RMS Kcal/Mol/Å, as described previously [103]. The two proteins were obtained from protein data bank (https://www.rcsb.org/ accessed on 15 November 2022), and the active sites of (PDB: 2NN7 and 1H9N) were separately generated (https://www.rcsb.org/structure/2NN7 accessed on 15 November 2022) (https://www.rcsb.org/structure/1H9N accessed on 15 November 2022). For *h*CA-I (PDB: 2NN7), the active site was generated using only one chain (chain A) using trigonal matcher replacement and London dG as the scoring function. The validation process was performed and the cocrystallized ligand ethyl 3-[4-(aminosulfonyl)phenyl] propanoate (M29) exhibited binding energy S = −4.65 Kcal/mol with RMSD = 1.477 Å through one hydrogen bond side chain donor between the residue Thr199 and the amino group of sulfonamide with a bond length of 2.10 Å, and the zinc metal ion bonded to one oxygen of sulfonamide. Additionally, for *h*CA-II (PDB: 1H9N), the active site was generated using a dummy atom under default protocol, with trigonal matcher replacement and London dG as the scoring function. Moreover, in the docking simulation for alpha and beta carbonic anhydrase (PDB: 5HPJ and 1I6P), respectively, the active site of these two proteins were downloaded from the protein data bank, and only one chain was selected and deleted. All the chai of waters for docking simulation and the active site was generated using a dummy atom standard protocol, then the selected pocket for each protein was separately protonated, and the minimized energy was performed with the trigonal matcher replacement and London dG as the scoring function.

## 4. Conclusions

A series of pyrazole and pyrazolo[1,5-*a*]pyrimidine derivatives were synthesized, and their antibacterial activity was determined by using the agar well-diffusion and broth microdilution (MICs and MBCs) methods. In the initial screening, all these derivatives used in the present study demonstrated effective inhibition profiles against the tested six multidrug-resistant Gram-positive and Gram-negative bacteria. Five compounds, 3a, 5a, 6, 9a, and 10a, exhibited the most significant antibacterial activity compared with the standard antibiotics, namely Erythromycin and Amikacin. These five compounds were further evaluated for their antibiofilm activity against *S. aureus* and *P. aeruginosa*, which showed strong biofilm-forming capability. They presented significant antibiofilm eradication at >60%, which was further confirmed microscopically by using an SEM analysis. Furthermore, the anti-QS activity against *S. aureus* and *P. aeruginosa* for the active pyrazole 3a and 5a and pyrazolo[1,5-*a*]pyrimidine derivatives 6, 9a, and 10a were screened using *Chromobacterium violaceum* 026 (CV026) as a biosensor strain. Interference with violacein (purple pigment) production in CV026 was used as an indication of the anti-QS activity; therefore, these five compounds exhibited promising anti-QS properties at the sub-MICs levels. Moreover, our study was extended to study the mode of action for the most active derivatives and identified both of the potent human CA isoenzymes (*h*CA-I and *h*CA-II) as inhibitors. In this study, a low nanomolar level of the IC_50_ and *Ki* values was observed for each pyrazole derivative. Our data indicated that the novel pyrazole and pyrazolo[1,5-*a*]pyrimidine derivatives could serve as potential candidates for use as anti-infective agents. Moreover, it has been found that gamma radiation could sterilize the active derivatives using doses of up to 20.0 kGy without causing any degradation in the physicochemical properties. According to docking studies, compounds 3a, 5a, 6, 9a, and 10a have the potential to become lead molecules in drug discovery, supporting their promising *h*CA-I and *h*CA-II results. Finally, the most active derivatives displayed satisfactory ADMET performances, which may allow them to be developed as anti-MDR bacterial therapeutic agents.

## Figures and Tables

**Table 1:** Average zones of inhibition (AZOIs) of novel pyrazole, pyrazolo[1,5-*a*]pyrimidine derivatives, and control antibiotics against clinical pathogenic bacteria recorded in mm.

Compound No.	Diameter of Inhibition Zones in mm					
	Gram-Positive Bacteria			Gram-Negative Bacteria		
	*E. faecalis* DH-5478	*S. aureus* DH-432	*S. pyogenes* DH-3467	*A. baumannii* DH-243	*E. coli* DH-5987	*P. aeruginosa* DH-5698
**3a**	**30.6**	**31.1**	**31.5**	**29.3**	**32**	**30.8**
**3b**	24.4	25.2	24.7	23.1	26.9	25.1
**3c**	21.3	23.8	22.6	22.5	24.7	23.2
**4**	22.1	24.7	23.4	21.3	22.4	20.5
**5a**	**28.2**	**28.6**	**28.9**	**28.0**	**29.9**	**28.3**
**5b**	25.4	26.2	24.1	23.4	25.7	24.7
**6**	**29.5**	**29.5**	**30.2**	**28.8**	**31.0**	**28.8**
**7**	23.0	25.3	22.8	24.5	25.8	24.0
**8a**	20.6	22.0	21.4	19.7	23.4	21.6
**8b**	17.3	19.5	18.7	16.5	20.1	18.4
**8c**	15.1	16.1	15.4	15.2	17.8	16.3
**9a**	**28.7**	**29.1**	**29.3**	**28.4**	**30.4**	**28.5**
**9b**	24.3	25.0	25.7	23.0	26.5	25.4
**9c**	23.1	24.2	23.5	23.9	25.2	24.1
**10a**	**29.8**	**30.6**	**30.7**	**29.1**	**31.5**	**30.1**
**10b**	25.1	26.8	26.3	24.2	27.1	26.3
**E**	11	12	10.5	NA	NA	NA
**AK**	NA	NA	NA	13	11	14
**DMSO**	-ve	-ve	-ve	-ve	-ve	-ve

**E**: Erythromycin (S ≥ 23 mm, I = 14–22 mm, R ≤ 13, for *S. aureus* and *E. faecalis*) and (S ≥ 21 mm, I = 16–20, R ≤ 15, for *S. pyogenes*); **AK**: Amikacin (S ≥ 17 mm, I = 15–16 mm, R ≤ 14 mm); **DMSO**: dimethyl sulfoxide; **NA**: not applicable; **-ve**: no zones of inhibition.

**Table 2:** In vitro antibacterial activity for the most active pyrazole and pyrazolo[1,5-*a*]pyrimidine derivatives **3**–**10** represented as MIC in µg/mL and (MBC/MIC).

Compound No.	MIC (Mean ± SEM) in µg/mL (MBC/MIC)					
	Gram-Positive Bacteria			Gram-Negative Bacteria		
	*E. faecalis* DH-5478	*S. aureus* DH-432	*S. pyogenes* DH-3467	*A. baumannii* DH-243	*E. coli* DH-5987	*P. aeruginosa* DH-5698
**3a**	**0.125 ± 0.1(0.5)**	**0.125 ± 0.3(0.5)**	**0.125 ± 0.3(0.5)**	**0.25 ± 0.2(0.5)**	**0.0625 ± 0.4(0.5)**	**0.125 ± 0.2(0.5)**
**3b**	0.75 ± 0.2(0.5)	0.5 ± 0.1(1)	0.75 ± 0.4(1)	1 ± 0.3(0.5)	0.5 ± 0.2(1)	1 ± 0.2(0.5)
**3c**	1 ± 0.3 (1)	0.75 ± 0.2 (1)	0.75 ± 0.2(0.5)	2 ± 0.2(1)	0.75 ± 0.5(0.5)	1 ± 0.3(1)
**4**	2 ± 0.3(0.5)	1 ± 0.4(0.5)	1.5 ± 0.3(0.5)	2 ± 0.4(0.5)	0.75 ± 0.3(1)	1.5 ± 0.2(1)
**5a**	**0.5 ± 0.2(0.5)**	**0.25 ± 0.2(0.5)**	**0.5 ± 0.1(0.5)**	**0.5 ± 0.3(0.5)**	**0.25 ± 0.2(0.5)**	**0.5 ± 0.1(0.5)**
**5b**	1.5 ± 0.3(1)	1 ± 0.3(1)	1 ± 0.2(1)	2 ± 0.1(0.5)	1 ± 0.2(0.5)	2 ± 0.3(1)
**6**	**0.375 ± 0.2(0.5)**	**0.187 ± 0.1(0.5)**	**0.25 ± 0.3(0.5)**	**0.5 ± 0.1(0.5)**	**0.25 ± 0.1(0.5)**	**0.375 ± 0.4(0.5)**
**7**	2 ± 0.4(1)	1 ± 0.3(1)	1.5 ± 0.2(1)	2 ± 0.3(1)	1.5 ± 0.2(0.5)	2 ± 0.3(0.5)
**8a**	4 ± 0.3(1)	2 ± 0.2(1)	4 ± 0.4(1)	4 ± 0.2(2)	2 ± 0.2(1)	2 ± 0.3(1)
**8b**	4 ± 0.1(2)	4 ± 0.3(2)	8 ± 0.2(1)	8 ± 0.2(2)	2 ± 0.3(1)	4 ± 0.1(2)
**8c**	8 ± 0.2(2)	4 ± 0.1(1)	4 ± 0.2(2)	8 ± 0.4(2)	4 ± 0.1(2)	8 ± 0.3(2)
**9a**	**0.5 ± 0.1(0.5)**	**0.25 ± 0.2(0.5)**	**0.25 ± 0.2(0.5)**	**0.375 ± 0.2(0.5)**	**0.125 ± 0.1(0.5)**	**0.375 ± 0.3(0.5)**
**9b**	2 ± 0.2(1)	1.5 ± 0.2(1)	2 ± 0.3(0.5)	2 ± 0.1(1)	1 ± 0.3(0.5)	1.5 ± 0.2(0.5)
**9c**	4 ± 0.2(1)	2 ± 0.3(1)	2 ± 0.1(1)	4 ± 0.4(1)	1 ± 0.3(1)	2 ± 0.2(1)
**10a**	**0.187 ± 0.1(0.5)**	**0.125 ± 0.2(0.5)**	**0.187 ± 0.2(0.5)**	**0.375 ± 0.4(0.5)**	**0.125 ± 0.2(.5)**	**0.187 ± 0.1(0.5)**
**10b**	2 ± 0.3(1)	1.5 ± 0.3(0.5)	1.5 ± 0.2(0.5)	2 ± 0.2(1)	0.75 ± 0.1(0.5)	1.5 ± 0.1(0.5)
**E**	32 ± 0.1(4)	8 ± 0.2(2)	64 ± 0.3(2)	NA	NA	NA
**AK**	NA	NA	NA	128 ± 0.2(4)	32 ± 0.2(4)	128 ± 0.1(4)
**DMSO**	-ve	-ve	-ve	-ve	-ve	-ve

**E**: Erythromycin (S ≤ 0.5, I = 1–4, R ≥ 8); **AK**: Amikacin (S ≤ 16, I = 32, R ≥ 64); **DMSO**: dimethyl sulfoxide; **NA**: not applicable; **-ve**: no zones of inhibition.

**Table 3:** Affinity chromatography purification scheme for the human erythrocyte carbonic anhy-drase-I and -II isoenzymes.

Purification Step	Activity (EU/mL)	Total Volume (mL)	Protein (mg/mL)	Total Protein (mg)	Total Activity (EU)	Specific Activity (EU/mg)	Yield (%)	Purification Factor
**Hemolysate**	140	90	15.9	1370	12,122	9.23	100	1
***h*** **CA-I**	372	15	0.5	4.5	3691	950	55	100
***h*** **CA-II**	800	10	0.2	0.85	4857	8300	62	900

**Table 4:** The enzyme inhibition data of human carbonic anhydrase isoenzymes (hCA-I and -II) with the most active pyrazole, pyrazolo[1,5-*a*]pyrimidine derivatives, and Acetazolamide.

Compound No.	*h*CA-I		*h*CA-II	
	IC_50_ (nM)	*K_i_* (nM)	IC_50_ (nM)	*K_i_* (nM)
**3a**	92.34	88.78	73.2	91.85
**5a**	137.99	176.21	161.22	144.27
**6**	168.84	153.46	130.29	145.58
**9a**	123.41	101.55	142.28	135.78
**10a**	153.45	143.59	121.98	139.37
**ACZ ^a^**	994.78	1052.22	900.33	981.64

^a^: Acetazolamide was used as a standard inhibitor for both CA-I and CA-II isoenzymes (*h*CA-I and -II).

**Table 5:** The microbial count (CFU/g) of the nonirradiated and irradiated pyrazole and pyrazolo[1,5-*a*]pyrimidine derivatives.

Dose (kGy)	Total Count CFU/g									
	3a		5a		6		9a		10a	
	Fungi	Bacteria	Fungi	Bacteria	Fungi	Bacteria	Fungi	Bacteria	Fungi	Bacteria
**Control**	1.5 × 10^1^	2 × 10^1^	2 × 10^2^	0	2 × 10^1^	3 × 10^1^	1.5 × 10^2^	0	1 × 10^2^	1 × 10^1^
**1**	1.5 × 10^1^	0	1 × 10^2^	0	1.5 × 10^1^	0	1 × 10^2^	0	1 × 10^1^	1 × 10^1^
**3**	1 × 10^1^	0	1 × 10^1^	0	0	0	0	0	1 × 10^1^	0
**5**	0	0	0	0	0	0	0	0	0	0
**7**	0	0	0	0	0	0	0	0	0	0
**10**	0	0	0	0	0	0	0	0	0	0
**15**	0	0	0	0	0	0	0	0	0	0
**20**	0	0	0	0	0	0	0	0	0	0

**Table 6:** Physicochemical properties, drug-likeness prediction, and medicinal chemistry of the most active pyrazole and pyrazolo[1,5-*a*]pyrimidine derivatives compared with acetazolamide as positive control.

Test Items		Most-Active Pyrazole and Pyrazolo[1,5-*a*]pyrimidine Derivatives Compared with Acetazolamide					
		3a	5a	6	9a	10a	ACZ
		Molecular Properties					
**SwissADME**	**Xlog P3**	1.52	3.62	2.77	3.68	4.01	-0.26
	**TPSA (** **°A^2^)**	131.74	121.32	107.23	160.28	154.49	151.66
	**M. Wt.**	274.28	405.45	338.36	469.50	470.48	222.25
	**nHBA (NO)**	5	6	6	7	8	6
	**nHBD (OHNH)**	3	2	1	2	2	2
	**NRB**	5	8	5	7	7	3
	**Log S (ESOL)**	−2.58	−4.53	−3.80	−5.05	−5.27	−1.14
	**Solubility Class**	Soluble	M Soluble	Soluble	M Soluble	M Soluble	Soluble
	**Drug-Likeness and Medicinal Chemistry Prediction**						
	**PAINS**	1 (N=N)	1 (N=N)	1 (N=N)	1 (N=N)	1 (N=N)	0
	**Lead-Likeness**	Yes	No (2)	Yes	No (2)	No (2)	No (1)
	**Synthetic Accessibility**	2.68	2.89	3.22	3.84	3.91	3.00
	**Bioavailability Score**	0.55	0.55	0.55	0.56	0.55	0.55
	**Lipinski Rule (Violation)**	Yes (0)	Yes (0)	Yes (0)	Yes (0)	Yes (0)	Yes (0)
	**Veber Rule (Violation)**	Yes (0)	Yes (0)	Yes (0)	No (1)	No (1)	No (1)

**Table 7:** In silico toxicity prediction of the most active pyrazole and pyrazolo[1,5-*a*]pyrimidine derivatives compared with acetazolamide as positive control.

Oral Toxicity Prediction		Most-Active Pyrazole and Pyrazolo[1,5-*a*]pyrimidine Derivatives and Acetazolamide as Positive Control					
		3a	5a	6	9a	10a	ACZ
	Oral Toxicity Prediction						
**ProTox-II prediction**	**LD_50_ mg/kg**	5000	500	928	300	300	4300
	**Toxicity Class**	V	IV	IV	III	III	V
	**Hepatotoxicity**	Inactive 0.50	Inactive 0.59	Inactive 0.62	Inactive 0.62	Inactive 0.60	Inactive 0.56
	**Carcinogenicity**	Active 0.60	Active 0.54	Active 0.65	Active 0.55	Inactive 0.56	Active 0.51
	**Immunotoxicity**	Inactive 0.99	Inactive 0.97	Inactive 0.99	Inactive 0.99	Inactive 0.99	Inactive 0.99
	**Cytotoxicity**	Inactive 0.74	Inactive 0.60	Inactive 0.61	Inactive 0.53	Inactive 0.70	Inactive 0.54
	**Aryl Hydrocarbon Receptor (AhR)**	Inactive 0.50	Inactive 0.54	Active 0.64	Inactive 0.94	Inactive 0.62	Inactive 0.99
	**Phosphoprotein** **(Tumor Suppressor) p53**	Inactive 0.84	Inactive 0.85	Inactive 0.80	Inactive 0.77	Inactive 0.86	Inactive 0.99

## Data Availability

Data are available as Supplementary Materials.

## Supplementary Materials

The following supporting information can be downloaded at https://www.mdpi.com/article/10.3390/antibiotics12010128/s1, all charts of ^1^H NMR and ^13^C NMR are represented in SI file.
