# Supplementary material for: Antibiofilm and Anti-Quorum-Sensing Activities of Novel Pyrazole and Pyrazolo[1,5-a]pyrimidine Derivatives as Carbonic Anhydrase I and II Inhibitors: Design, Synthesis, Radiosterilization, and Molecular Docking Studies
Source: Antibiotics (Basel). 2023 Jan 9;12(1):128. doi: 10.3390/antibiotics12010128 (PMC9854762; doi:10.3390/antibiotics12010128)
Supplement: Supplementary file 1 [file antibiotics-12-00128-s001.zip › antibiotics-2104420-supplementary.pdf]

Antibiofilm and anti-quorum sensing activities of novel pyrazole and pyrazolo[1,5-*a*]pyrimidine derivatives as carbonic anhydrase I and II inhibitors: Design, synthesis, radio-sterilization, and molecular docking studies

Ahmed Ragab[1,*], Sawsan A. Fouad[2], Yousry A. Ammar[1], Dina S. Aboul-Magd[3], and Moustafa S. Abusaif[1,*]

[1] Department of Chemistry, Faculty of Science (Boys), Al-Azhar University, Nasr City, 11884, Cairo, Egypt
[2] Department of Chemistry, Faculty of Science (Girls), Al-Azhar University, Nasr City, Cairo, Egypt
[3] Drug Radiation Research Department, National Center for Radiation Research and Technology (NCRRT), Egyptian Atomic Energy Authority, Egypt

***Correspondence:** Ahmed Ragab E-mail: ahmed_ragab7@ymail.com; ahmed_ragab@azhar.edu.eg Tel.: (0201009341359) and

Yousry A. Ammar (yossry@yahoo.com)

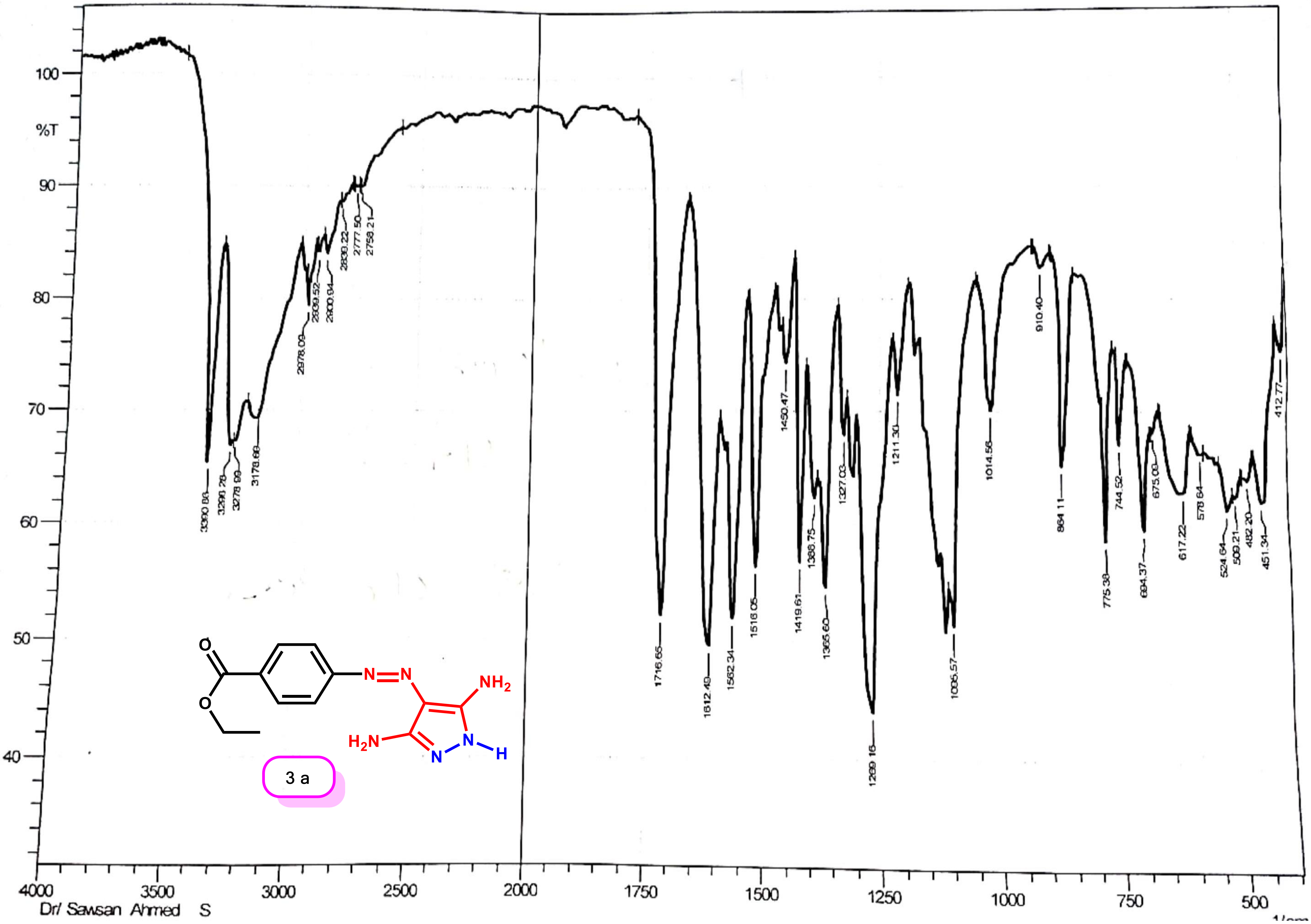
100
%T
90
80
70
60
50
40
2777.50
2758.21
1716.65
1612.49
1562.34
1518.05
1450.47
1419.61
1388.75
1365.60
1327.03
1269.16
1211.30
1014.56
910.40
864.11
775.38
744.52
694.37
617.22
578.64
524.64
509.21
482.20
451.34
412.77
N=N
NH2
H2N
N
N
H
O
O
4000
3500
3000
2500
2000
1750
1500
1250
1000
750
500
Dr/ Sawsan Ahmed S

3 a

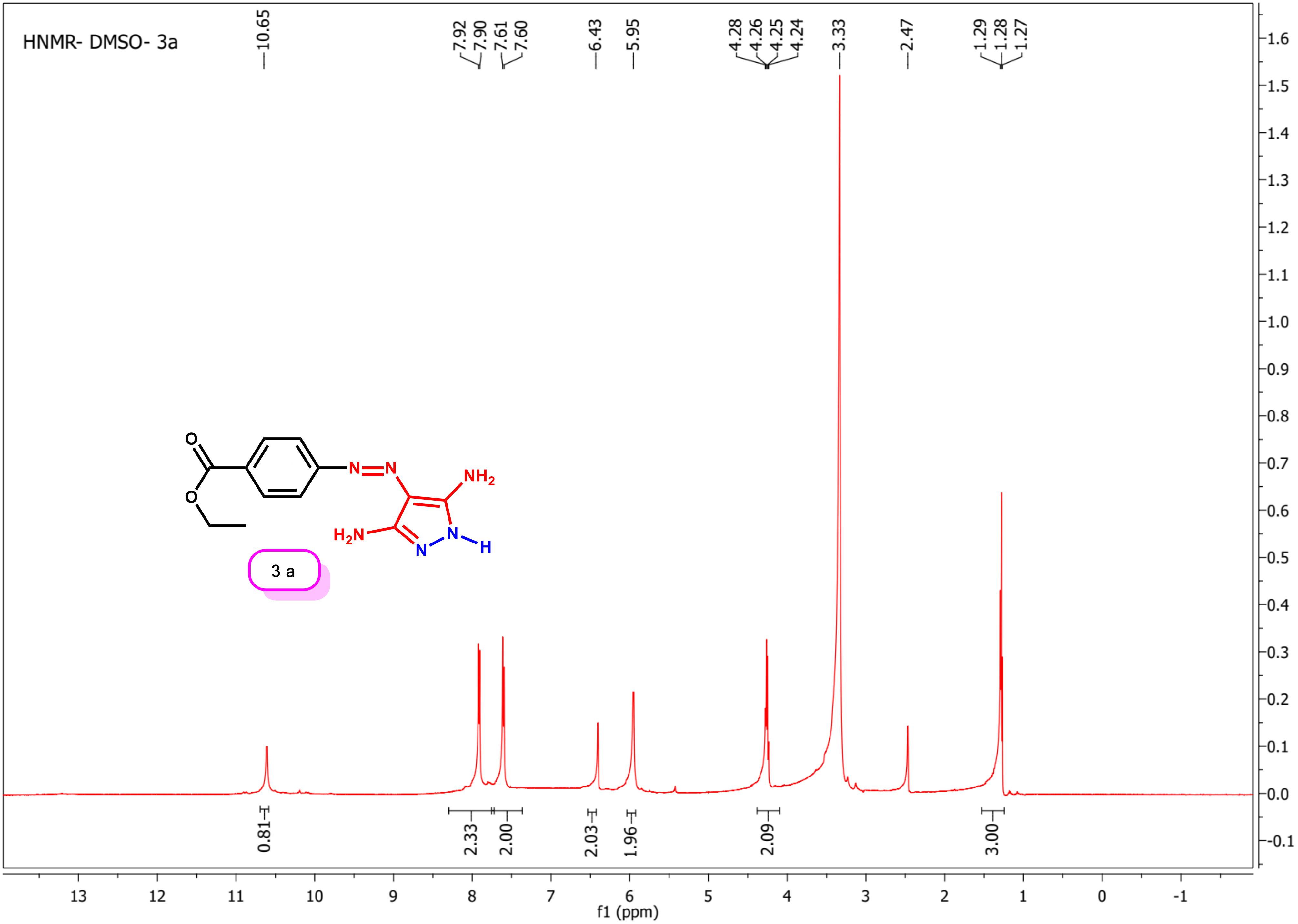
HNMR- DMSO- 3a
10.65
7.92
7.90
7.61
7.60
6.43
5.95
4.28
4.26
4.25
4.24
3.33
2.47
1.29
1.28
1.27
0.81
2.33
2.00
2.03
1.96
2.09
3.00
3 a
f1 (ppm)

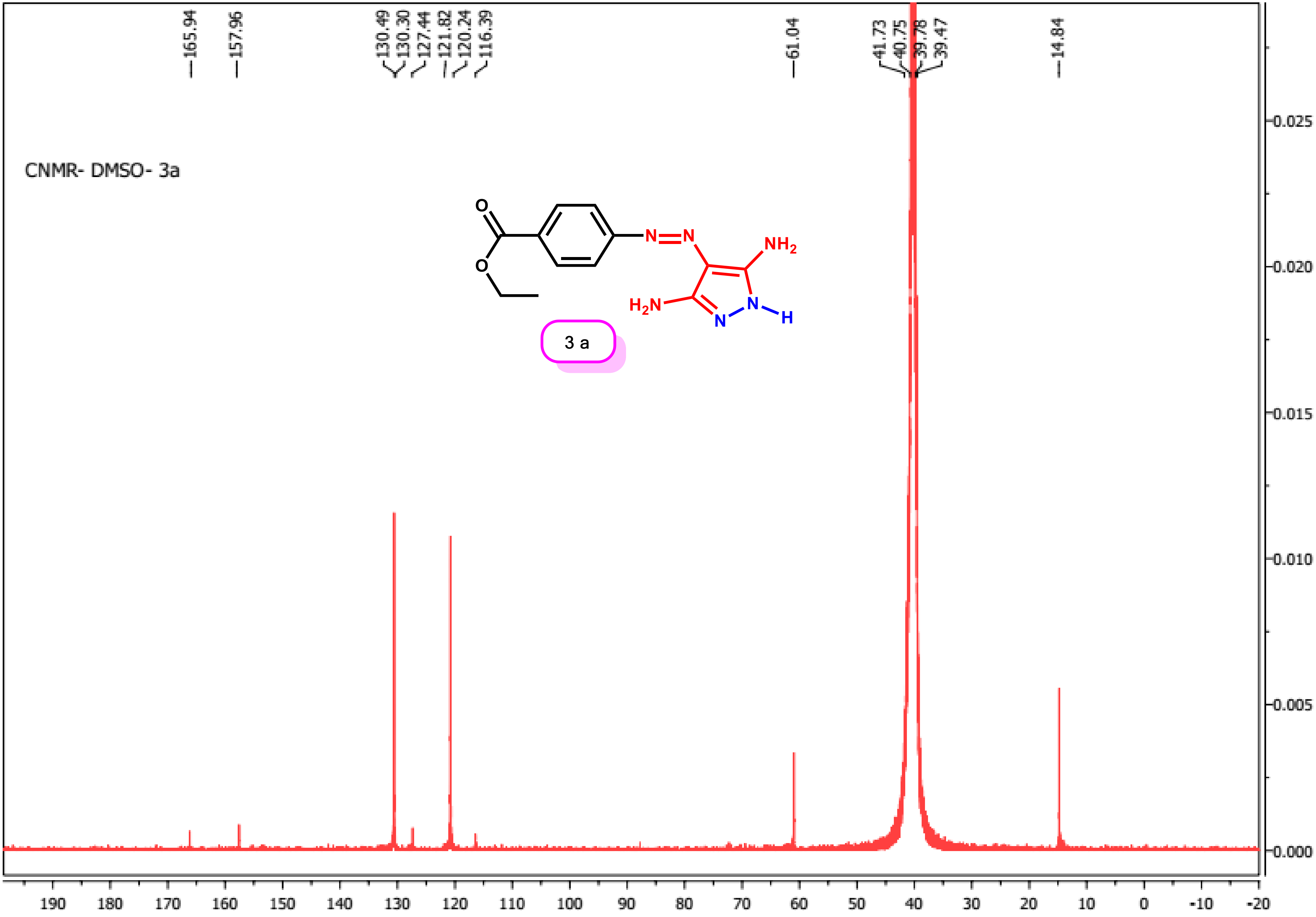

CNMR- DMSO- 3a
165.94
157.96
130.49
130.30
127.44
121.82
120.24
116.39
61.04
41.73
40.75
39.78
39.47
14.84
O
O
N
N
NH2
H2N
N
N
H
3 a
0.025
0.020
0.015
0.010
0.005
0.000
190
180
170
160
150
140
130
120
110
100
90
80
70
60
50
40
30
20
10
0
-10
-20

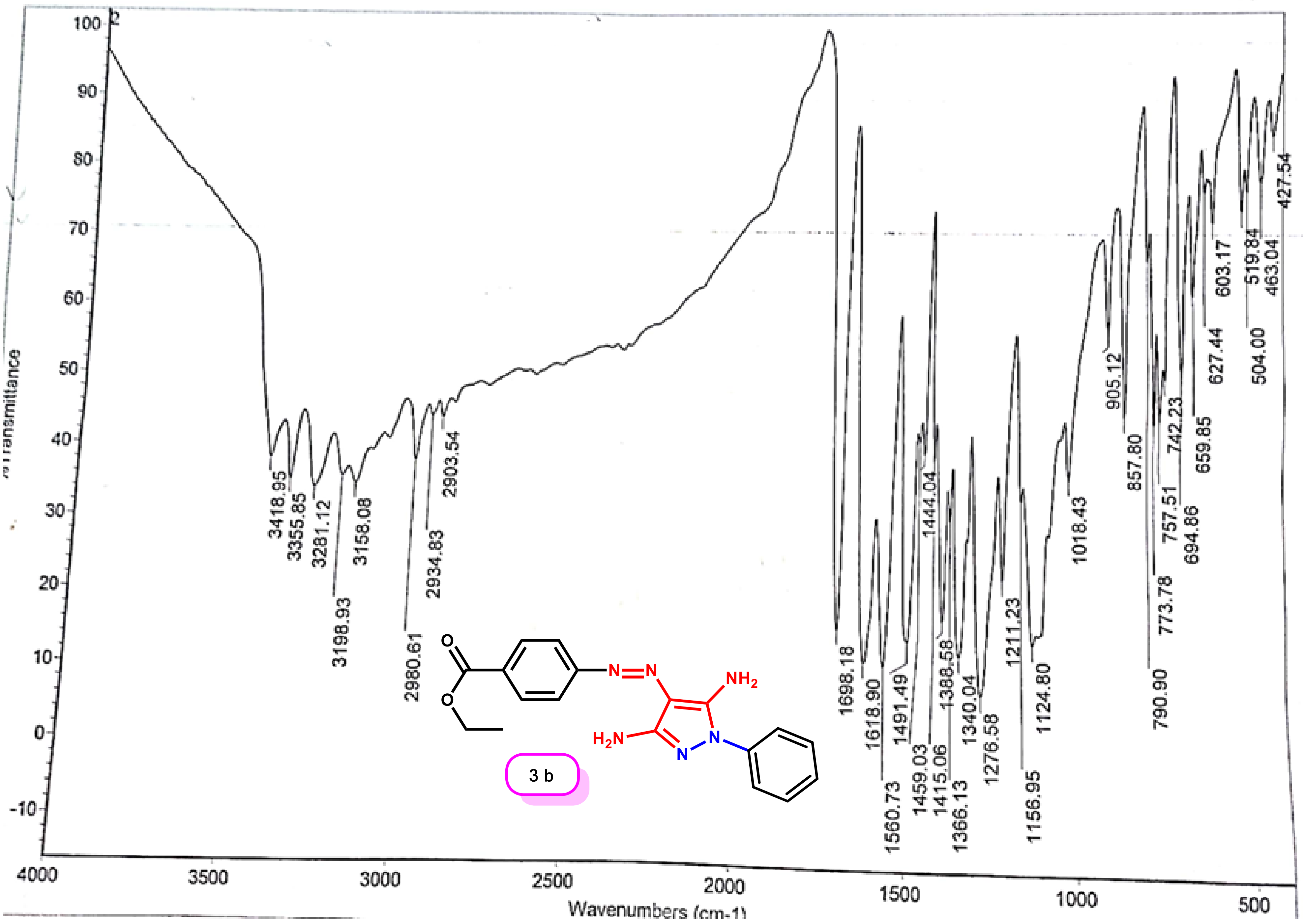

%Transmittance
Wavenumbers (cm-1)
4000
3500
3000
2500
2000
1500
1000
500
100
90
80
70
60
50
40
30
20
10
0
-10
3418.95
3355.85
3281.12
3198.93
3158.08
2980.61
2934.83
2903.54
1698.18
1618.90
1560.73
1491.49
1459.03
1444.04
1415.06
1388.58
1366.13
1340.04
1276.58
1211.23
1156.95
1124.80
1018.43
905.12
857.80
790.90
773.78
757.51
742.23
694.86
659.85
627.44
603.17
519.84
504.00
463.04
427.54
3 b

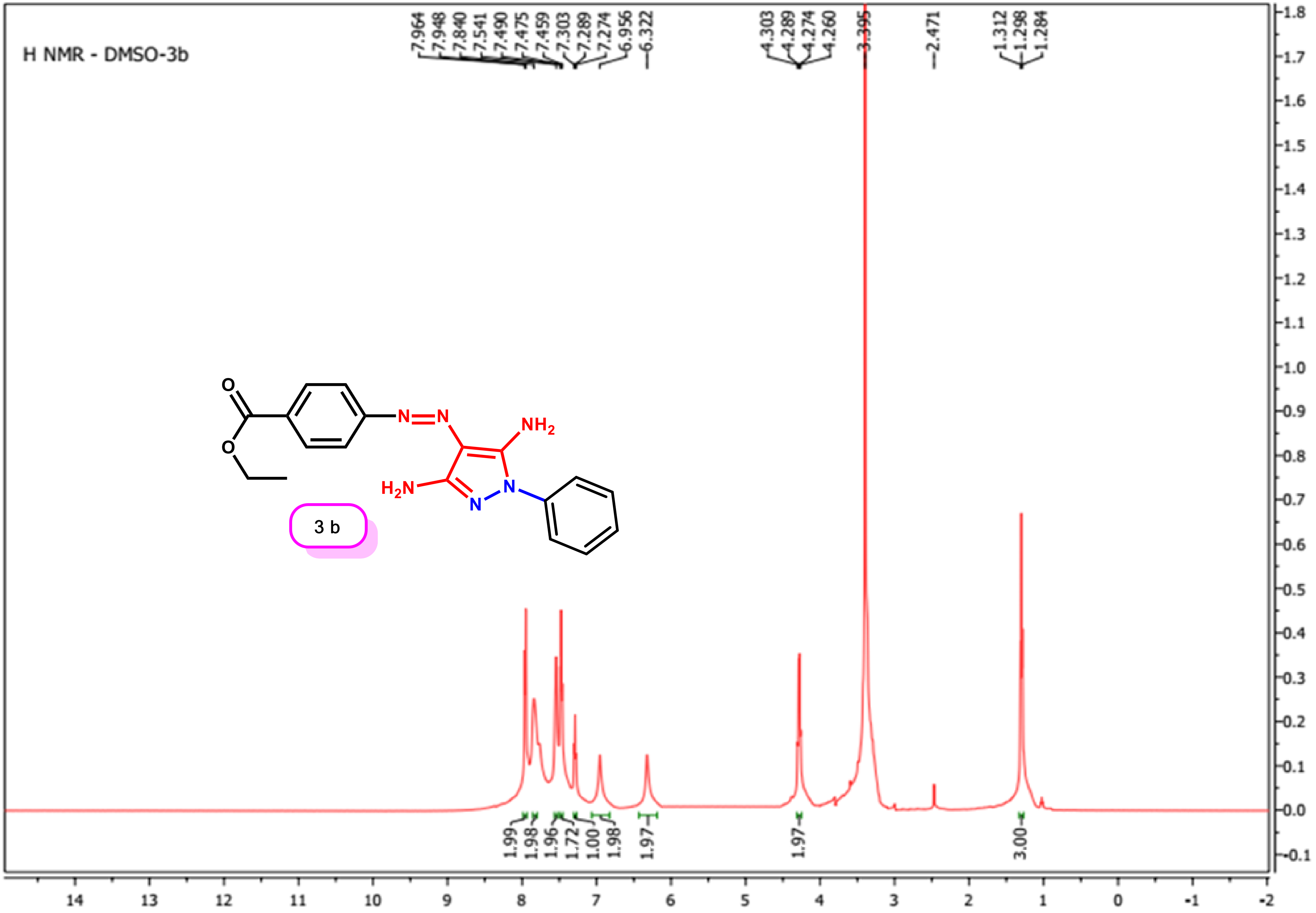
H NMR - DMSO-3b
7.964
7.948
7.840
7.541
7.490
7.475
7.459
7.303
7.289
7.274
6.956
6.322
4.303
4.289
4.274
4.260
3.395
2.471
1.312
1.298
1.284
O
O
N=N
NH2
H2N
N
N
3 b
1.99
1.98
1.96
1.72
1.00
1.98
1.97
1.97
3.00
14
13
12
11
10
9
8
7
6
5
4
3
2
1
0
-1
-2
1.8
1.7
1.6
1.5
1.4
1.3
1.2
1.1
1.0
0.9
0.8
0.7
0.6
0.5
0.4
0.3
0.2
0.1
0.0
-0.1

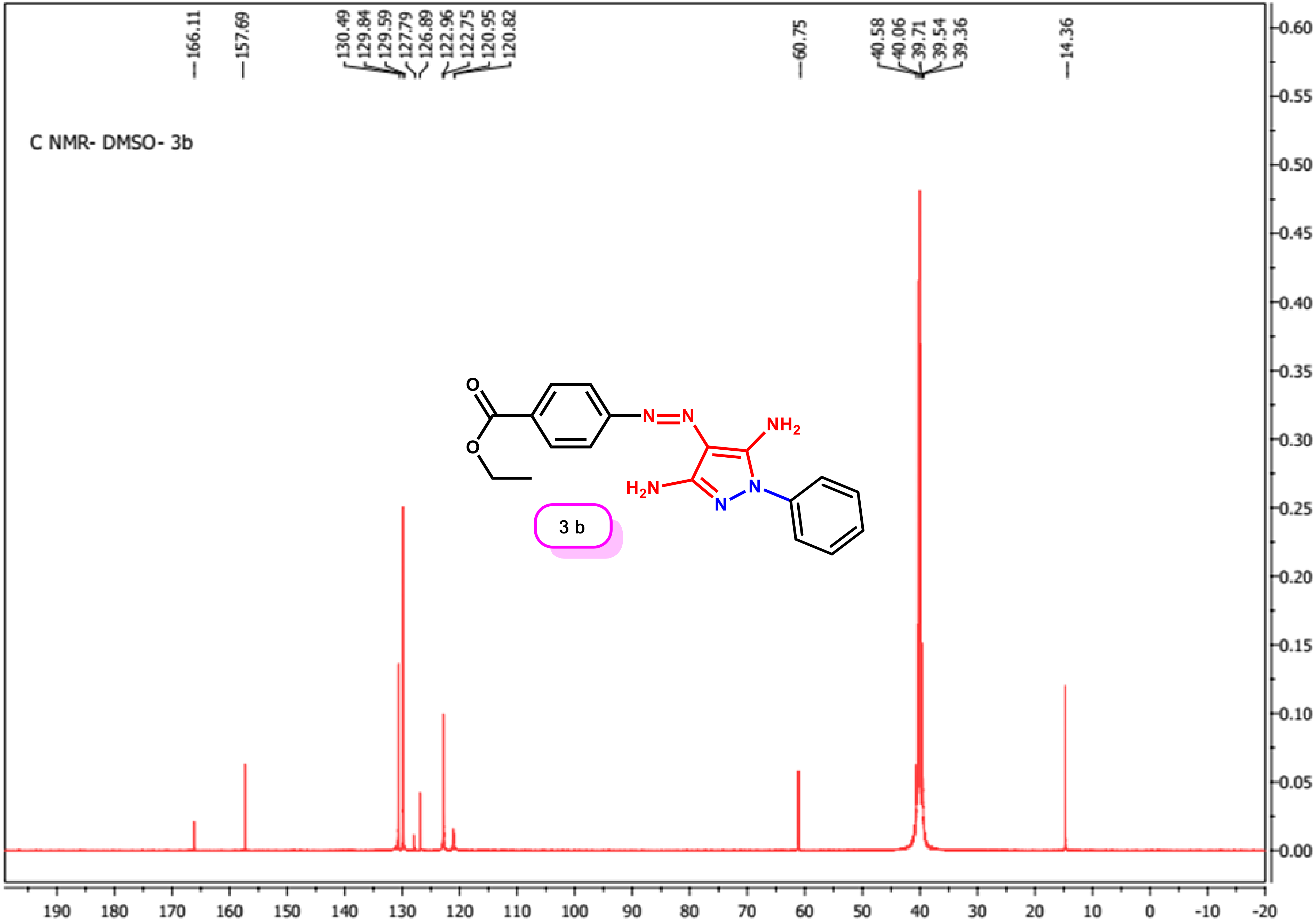

166.11
157.69
130.49
129.84
129.59
127.79
126.89
122.96
122.75
120.95
120.82
60.75
40.58
40.06
39.71
39.54
39.36
14.36
C NMR- DMSO- 3b
O
O
N
N
NH2
H2N
N
N
3 b
0.60
0.55
0.50
0.45
0.40
0.35
0.30
0.25
0.20
0.15
0.10
0.05
0.00
190
180
170
160
150
140
130
120
110
100
90
80
70
60
50
40
30
20
10
0
-10
-20

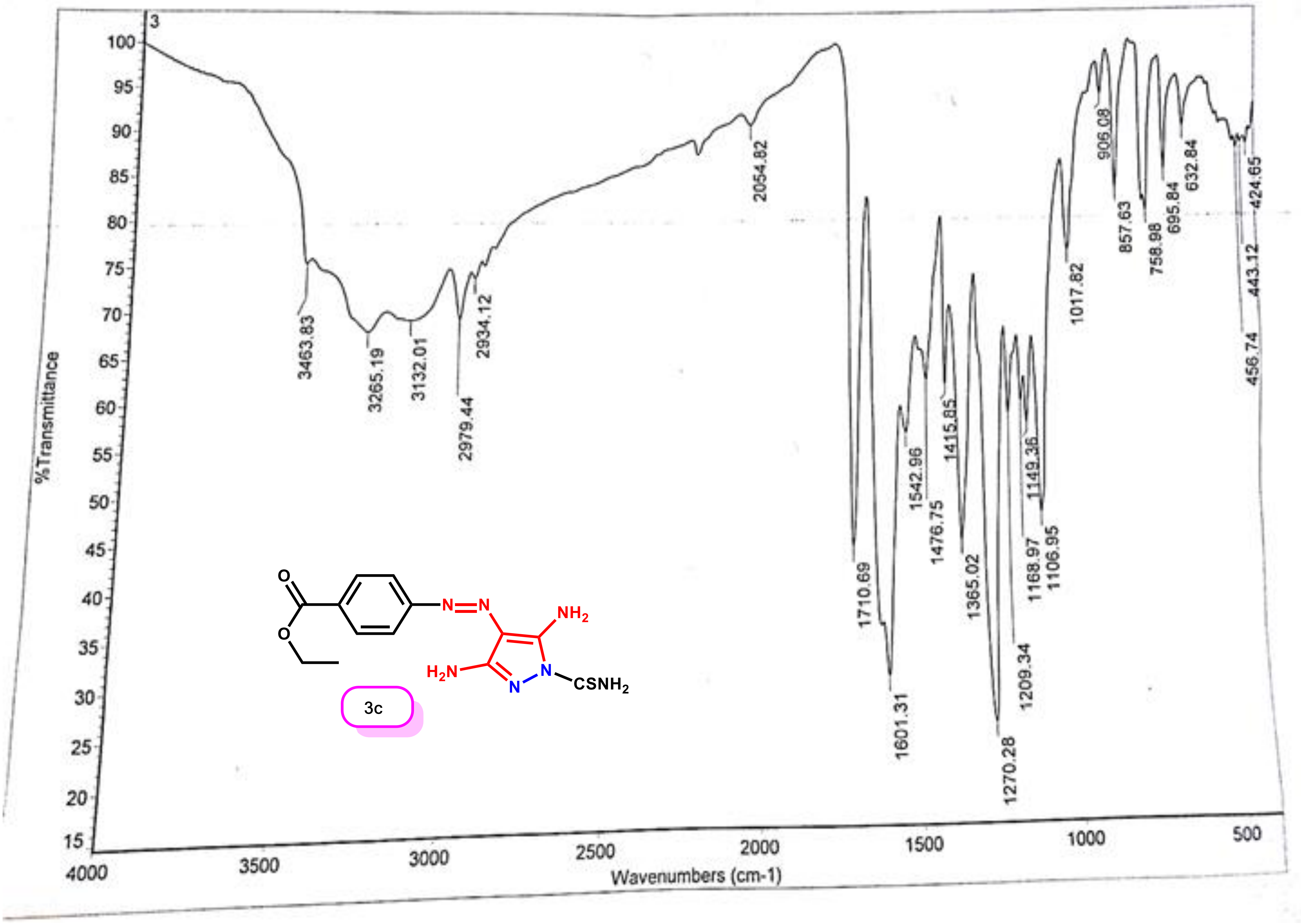
3
%Transmittance
Wavenumbers (cm-1)
3463.83
3265.19
3132.01
2979.44
2934.12
2054.82
1710.69
1601.31
1542.96
1476.75
1415.85
1365.02
1270.28
1209.34
1168.97
1149.36
1106.95
1017.82
906.08
857.63
758.98
695.84
632.84
456.74
443.12
424.65
O
O
N=N
NH2
H2N
N
N
CSNH2
3c

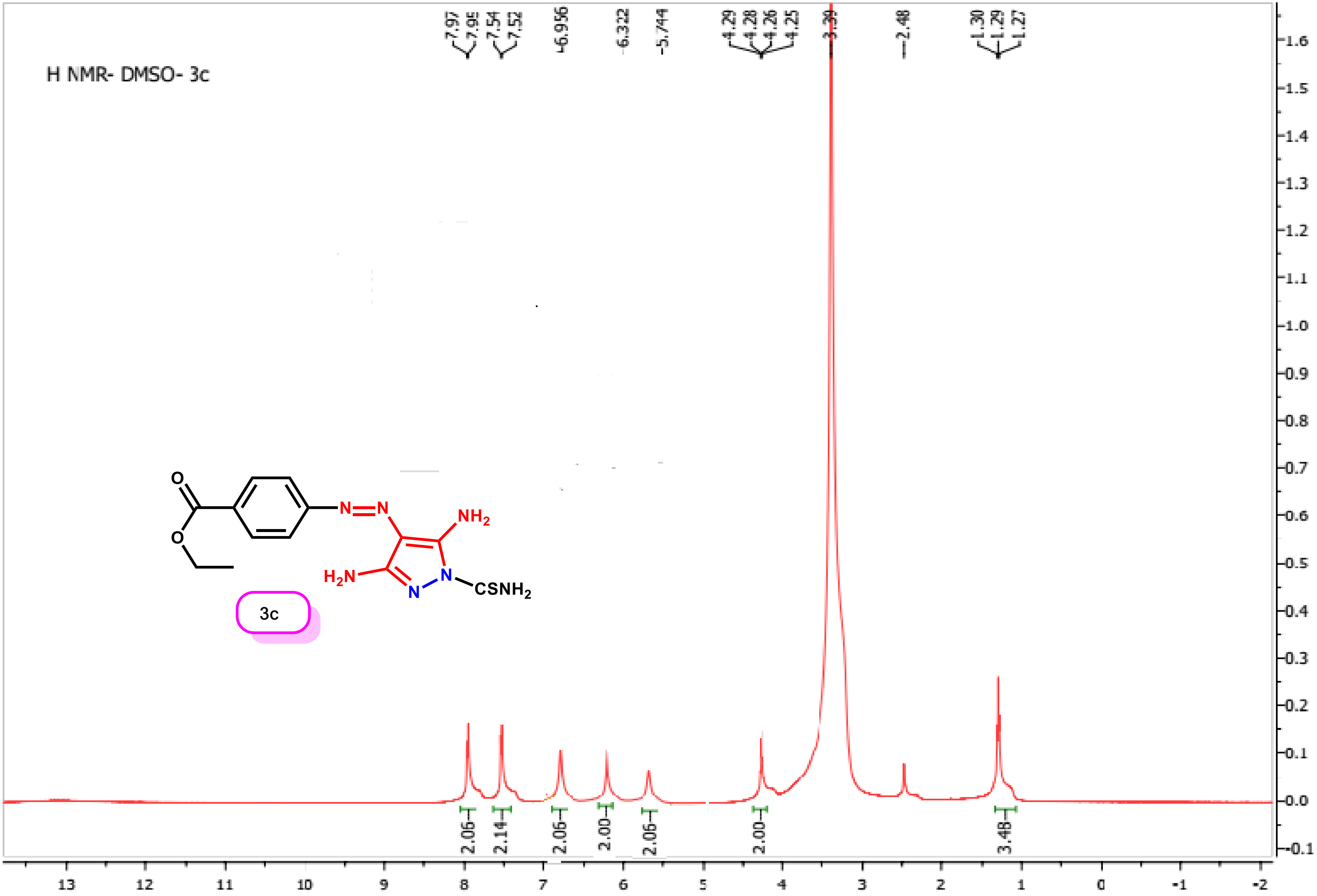
H NMR- DMSO- 3c
7.97
7.95
7.54
7.52
6.956
6.322
5.744
4.29
4.28
4.26
4.25
3.39
2.48
1.30
1.29
1.27
2.06
2.14
2.06
2.00
2.06
2.00
3.48
N=N
NH2
H2N
N
N
CSNH2
O
O
3c
13
12
11
10
9
8
7
6
5
4
3
2
1
0
-1
-2
1.6
1.5
1.4
1.3
1.2
1.1
1.0
0.9
0.8
0.7
0.6
0.5
0.4
0.3
0.2
0.1
0.0
-0.1

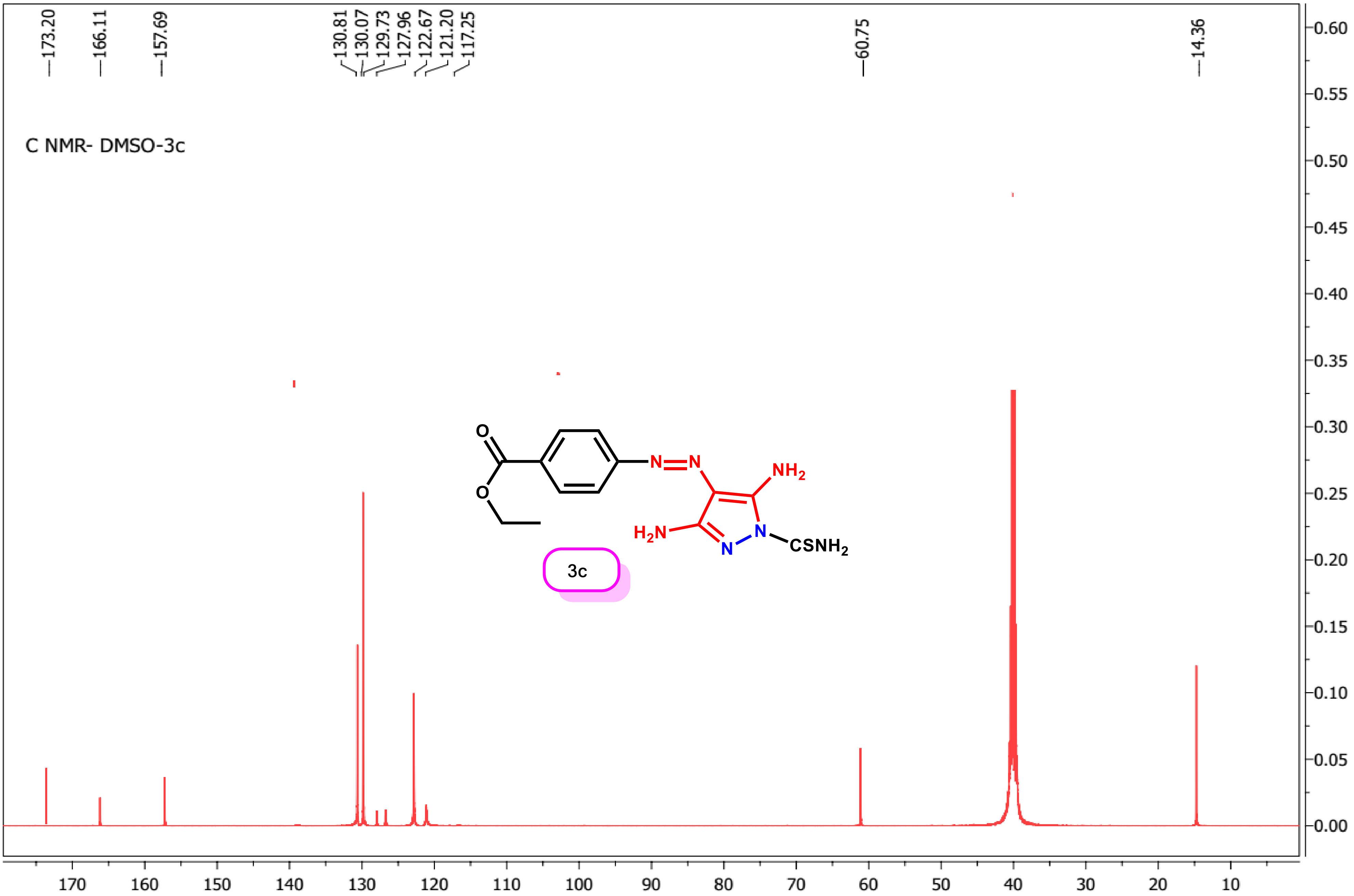

173.20
166.11
157.69
130.81
130.07
129.73
127.96
122.67
121.20
117.25
60.75
14.36
C NMR- DMSO-3c
O
O
N
N
NH2
H2N
N
N
CSNH2
3c
0.60
0.55
0.50
0.45
0.40
0.35
0.30
0.25
0.20
0.15
0.10
0.05
0.00
170
160
150
140
130
120
110
100
90
80
70
60
50
40
30
20
10

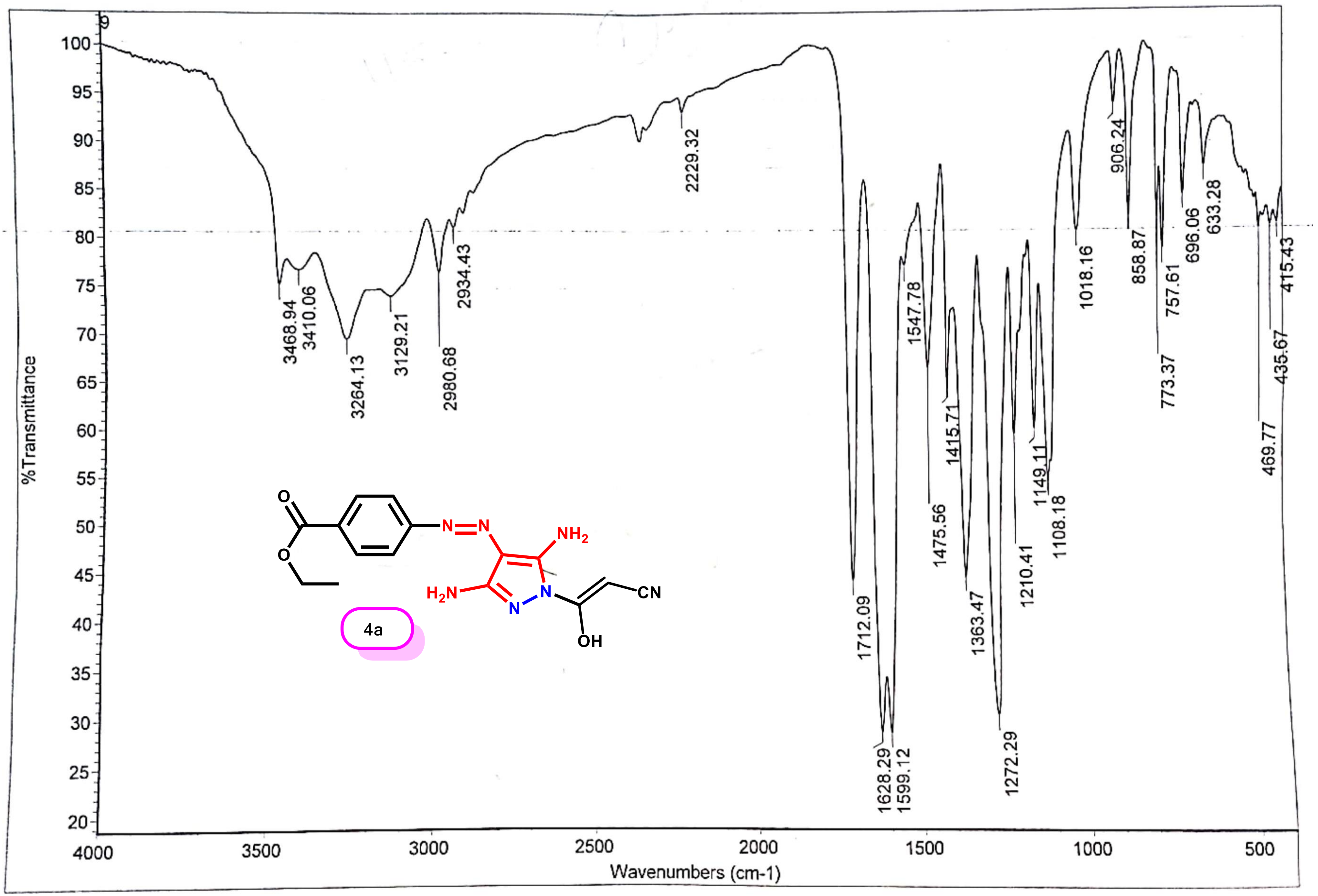

%Transmittance
Wavenumbers (cm-1)
100
95
90
85
80
75
70
65
60
55
50
45
40
35
30
25
20
4000
3500
3000
2500
2000
1500
1000
500
3468.94
3410.06
3264.13
3129.21
2980.68
2934.43
2229.32
1712.09
1628.29
1599.12
1547.78
1475.56
1415.71
1363.47
1272.29
1210.41
1149.11
1108.18
1018.16
906.24
858.87
773.37
757.61
696.06
633.28
469.77
435.67
415.43
O
O
N
N
NH2
H2N
N
N
CN
OH
4a

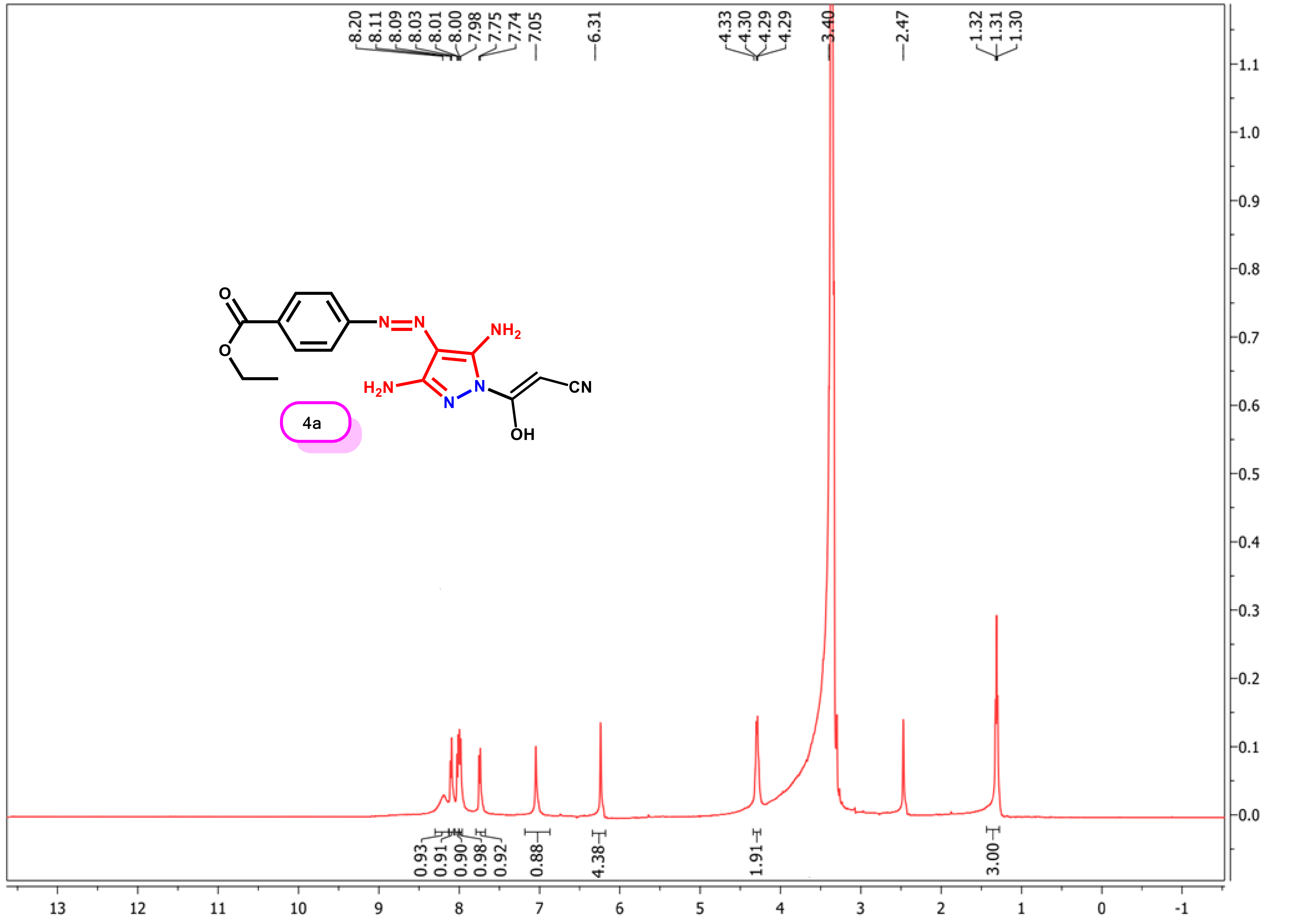
8.20
8.11
8.09
8.03
8.01
8.00
7.98
7.75
7.74
7.05
6.31
4.33
4.30
4.29
4.29
3.40
2.47
1.32
1.31
1.30
O
O
N=N
NH2
H2N
N
N
CN
OH
4a
0.93
0.91
0.90
0.98
0.92
0.88
4.38
1.91
3.00
1.1
1.0
0.9
0.8
0.7
0.6
0.5
0.4
0.3
0.2
0.1
0.0
13
12
11
10
9
8
7
6
5
4
3
2
1
0
-1

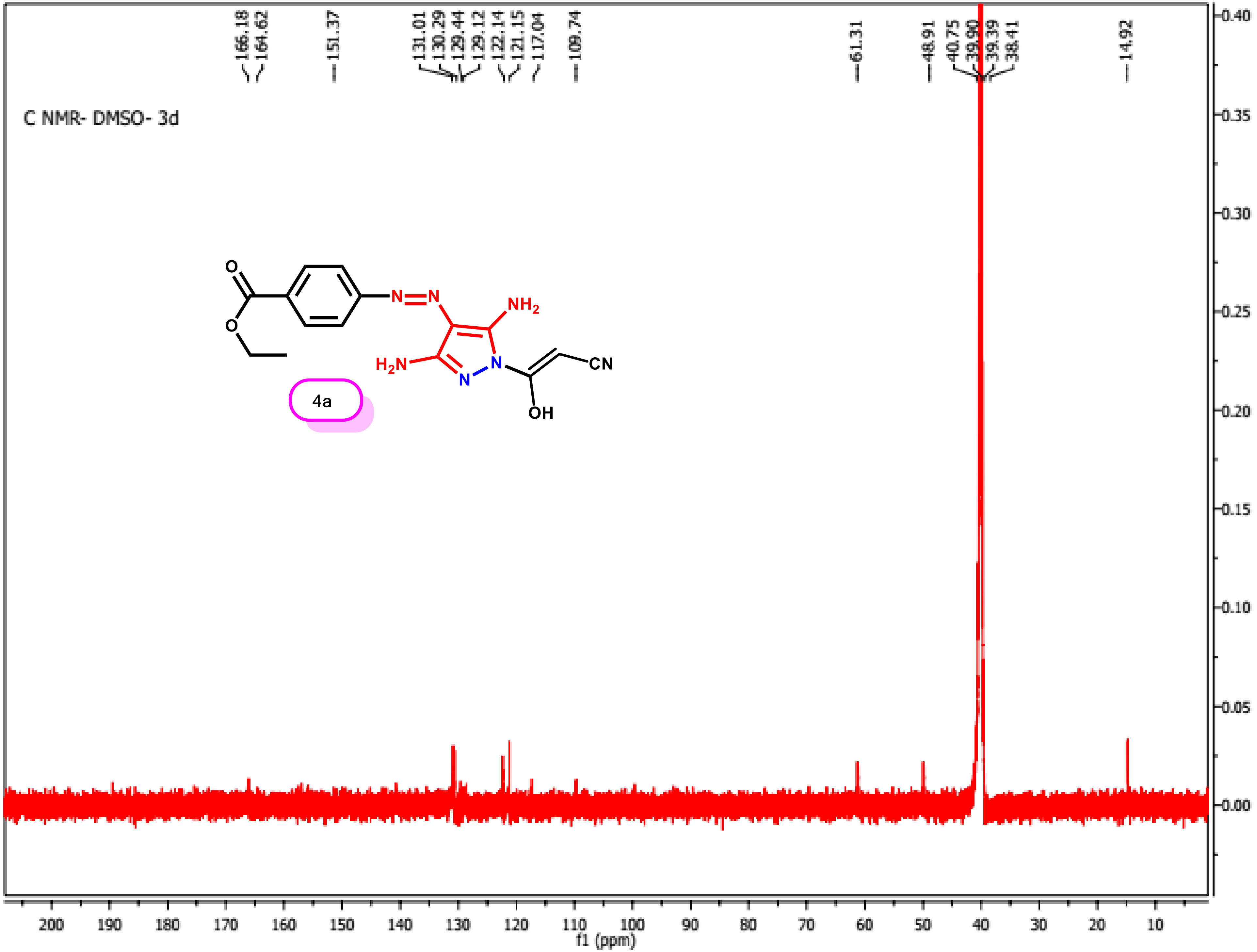

166.18
164.62
151.37
131.01
130.29
129.44
129.12
122.14
121.15
117.04
109.74
61.31
48.91
40.75
39.90
39.39
38.41
14.92
C NMR- DMSO- 3d
O
O
N=N
NH2
H2N
N
N
CN
OH
4a
f1 (ppm)

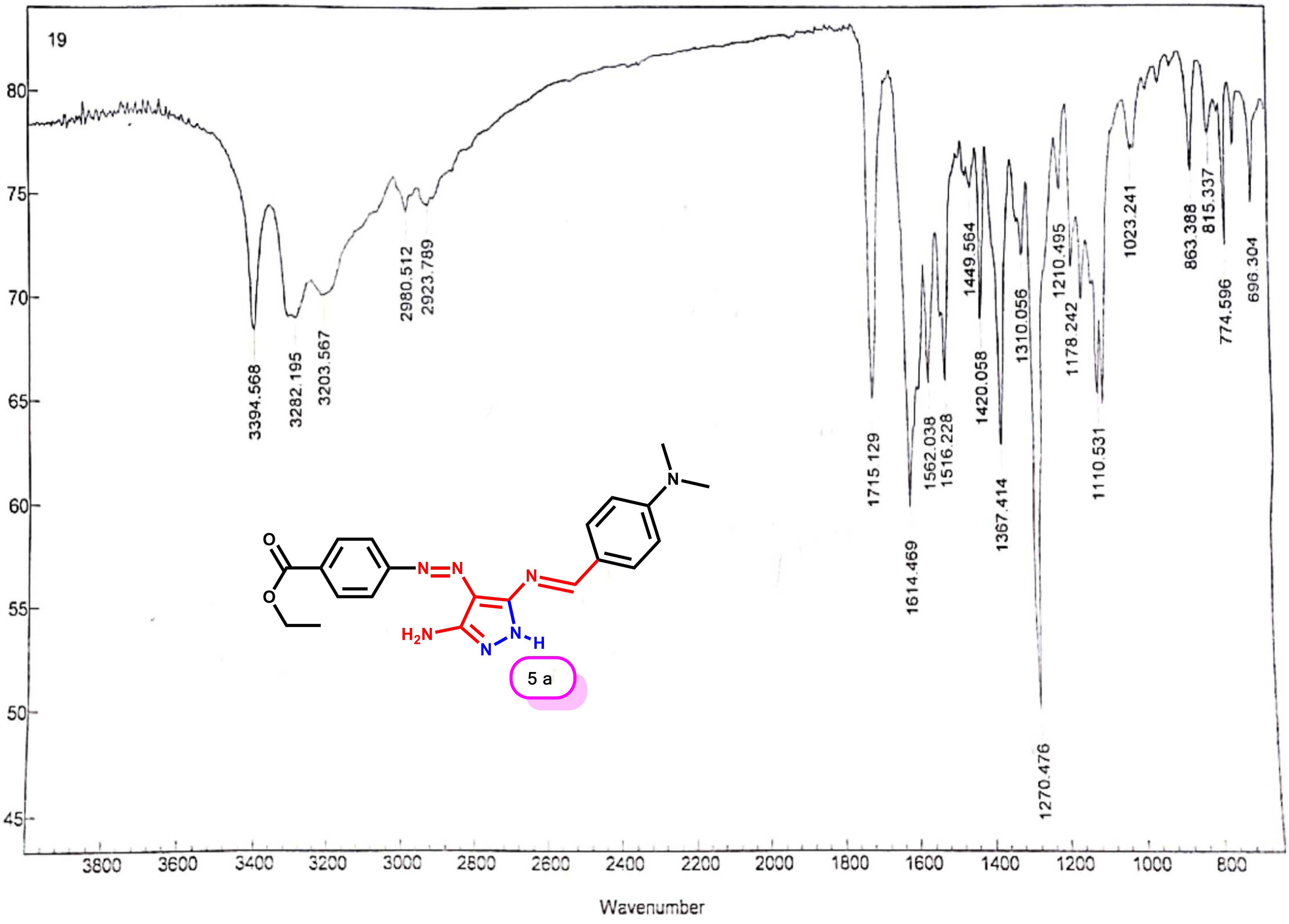
19
80
75
70
65
60
55
50
45
3394.568
3282.195
3203.567
2980.512
2923.789
1715.129
1614.469
1562.038
1516.228
1449.564
1420.058
1367.414
1310.056
1270.476
1210.495
1178.242
1110.531
1023.241
863.388
815.337
774.596
696.304
3800
3600
3400
3200
3000
2800
2600
2400
2200
2000
1800
1600
1400
1200
1000
800
Wavenumber
O
O
N=N
N
N
H₂N
N
N-H
5 a

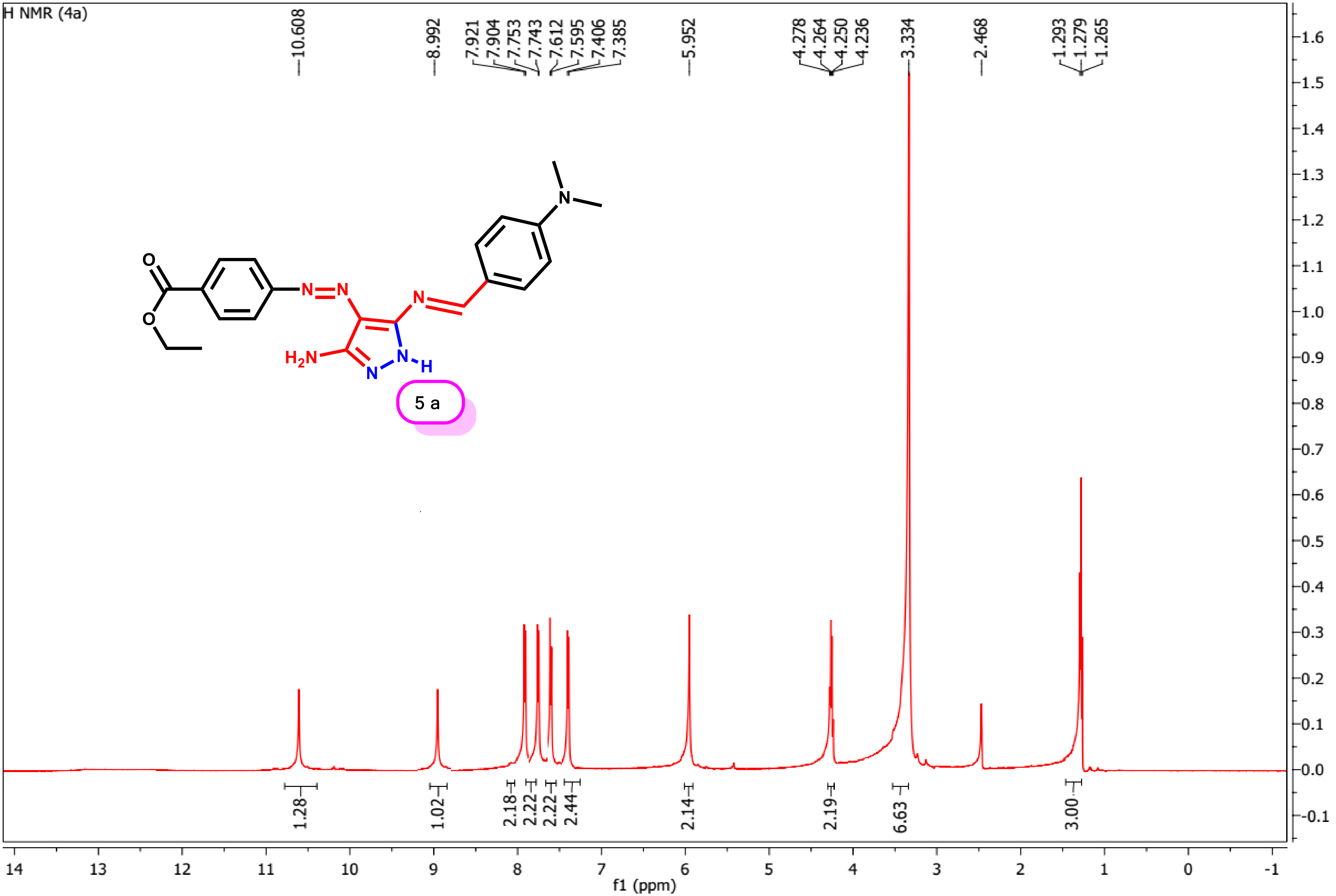
H NMR (4a)
10.608
8.992
7.921
7.904
7.753
7.743
7.612
7.595
7.406
7.385
5.952
4.278
4.264
4.250
4.236
3.334
2.468
1.293
1.279
1.265
O
O
N=N
N
H_2N
N
N
H
5 a
N
1.28
1.02
2.18
2.22
2.22
2.44
2.14
2.19
6.63
3.00
f1 (ppm)

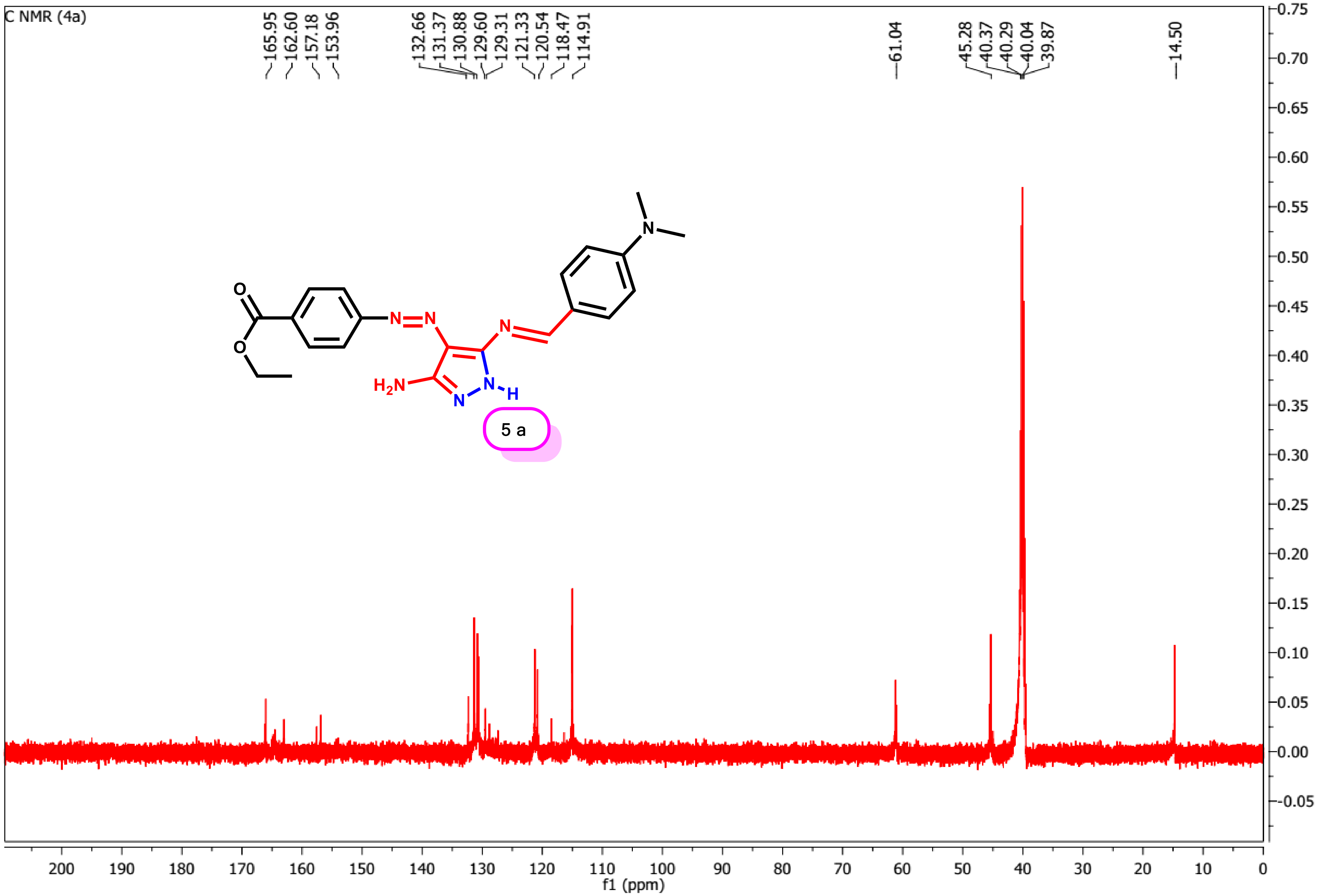
C NMR (4a)
165.95
162.60
157.18
153.96
132.66
131.37
130.88
129.60
129.31
121.33
120.54
118.47
114.91
61.04
45.28
40.37
40.29
40.04
39.87
14.50
5 a
f1 (ppm)

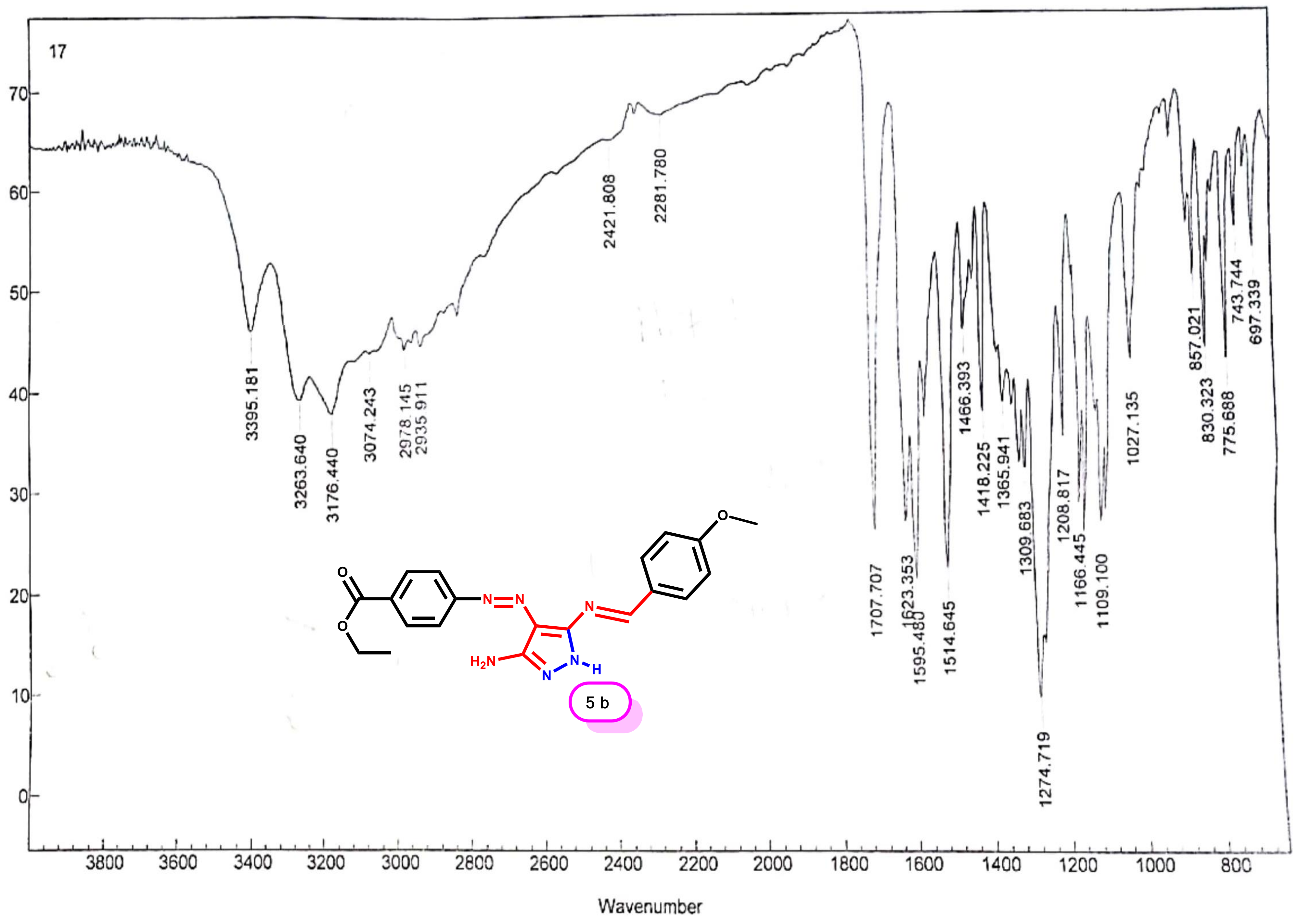

17
70
60
50
40
30
20
10
0
3395.181
3263.640
3176.440
3074.243
2978.145
2935.911
2421.808
2281.780
1707.707
1623.353
1595.480
1514.645
1466.393
1418.225
1365.941
1309.683
1274.719
1208.817
1166.445
1109.100
1027.135
857.021
830.323
775.688
743.744
697.339
3800
3600
3400
3200
3000
2800
2600
2400
2200
2000
1800
1600
1400
1200
1000
800
Wavenumber
O
O
N=N
N
H_2N
N
N
H
O
5 b

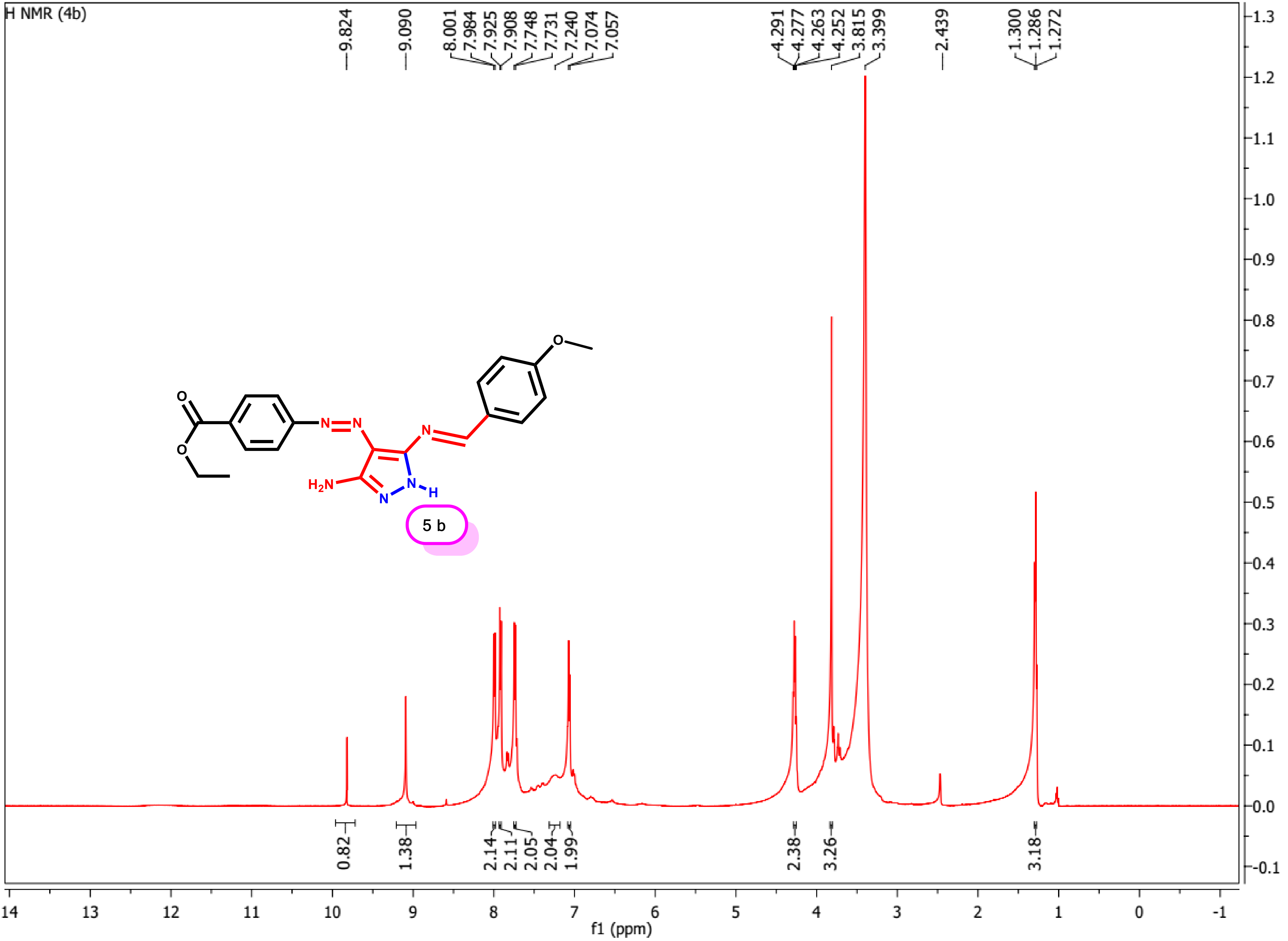
H NMR (4b)
9.824
9.090
8.001
7.984
7.925
7.908
7.748
7.731
7.240
7.074
7.057
4.291
4.277
4.263
4.252
3.815
3.399
2.439
1.300
1.286
1.272
0.82
1.38
2.14
2.11
2.05
2.04
1.99
2.38
3.26
3.18
O
N
H₂N
H
5 b
f1 (ppm)

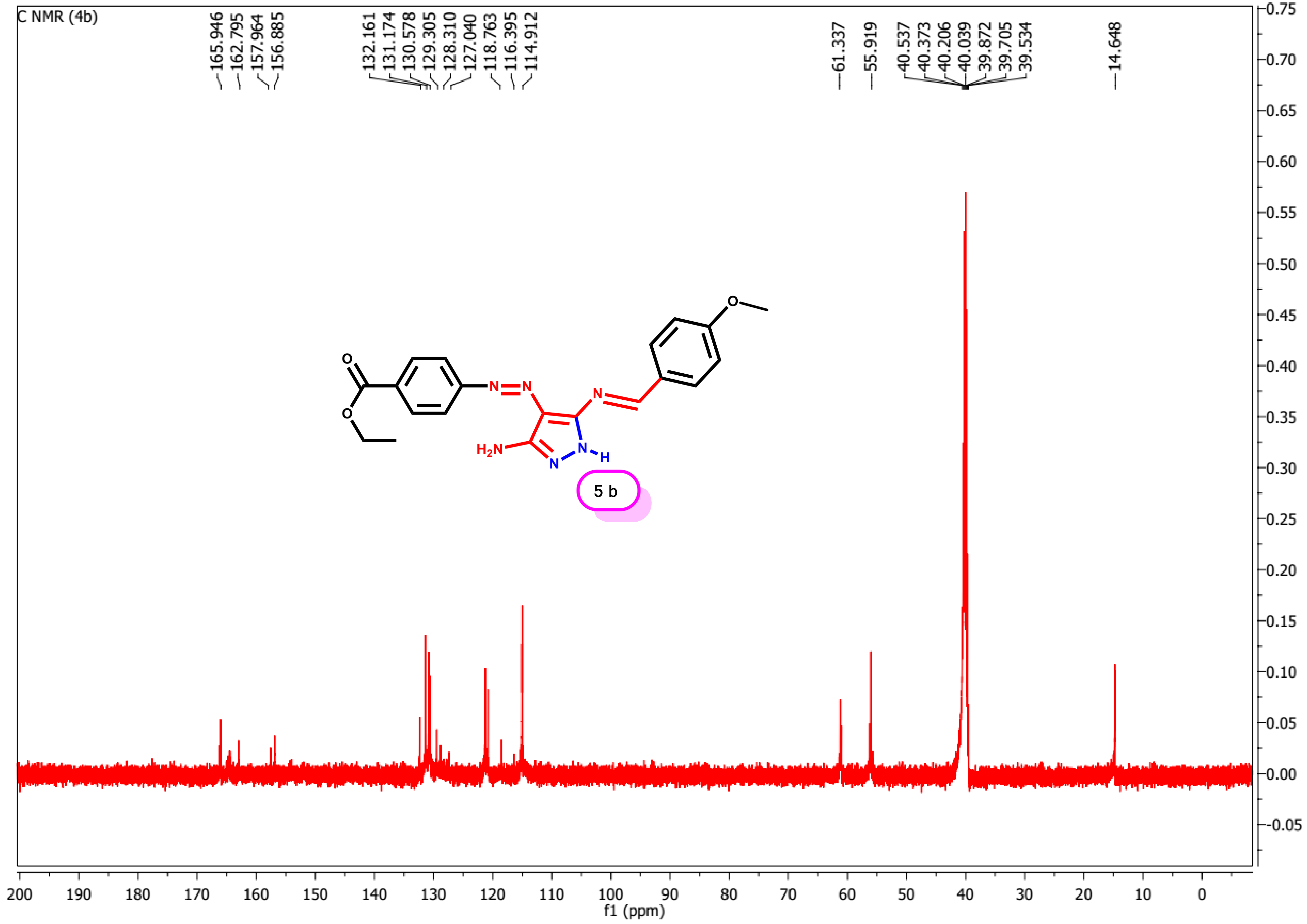
C NMR (4b)
165.946
162.795
157.964
156.885
132.161
131.174
130.578
129.305
128.310
127.040
118.763
116.395
114.912
61.337
55.919
40.537
40.373
40.206
40.039
39.872
39.705
39.534
14.648
O
O
N
N
N
H$_2$N
N
N
H
O
5 b
f1 (ppm)

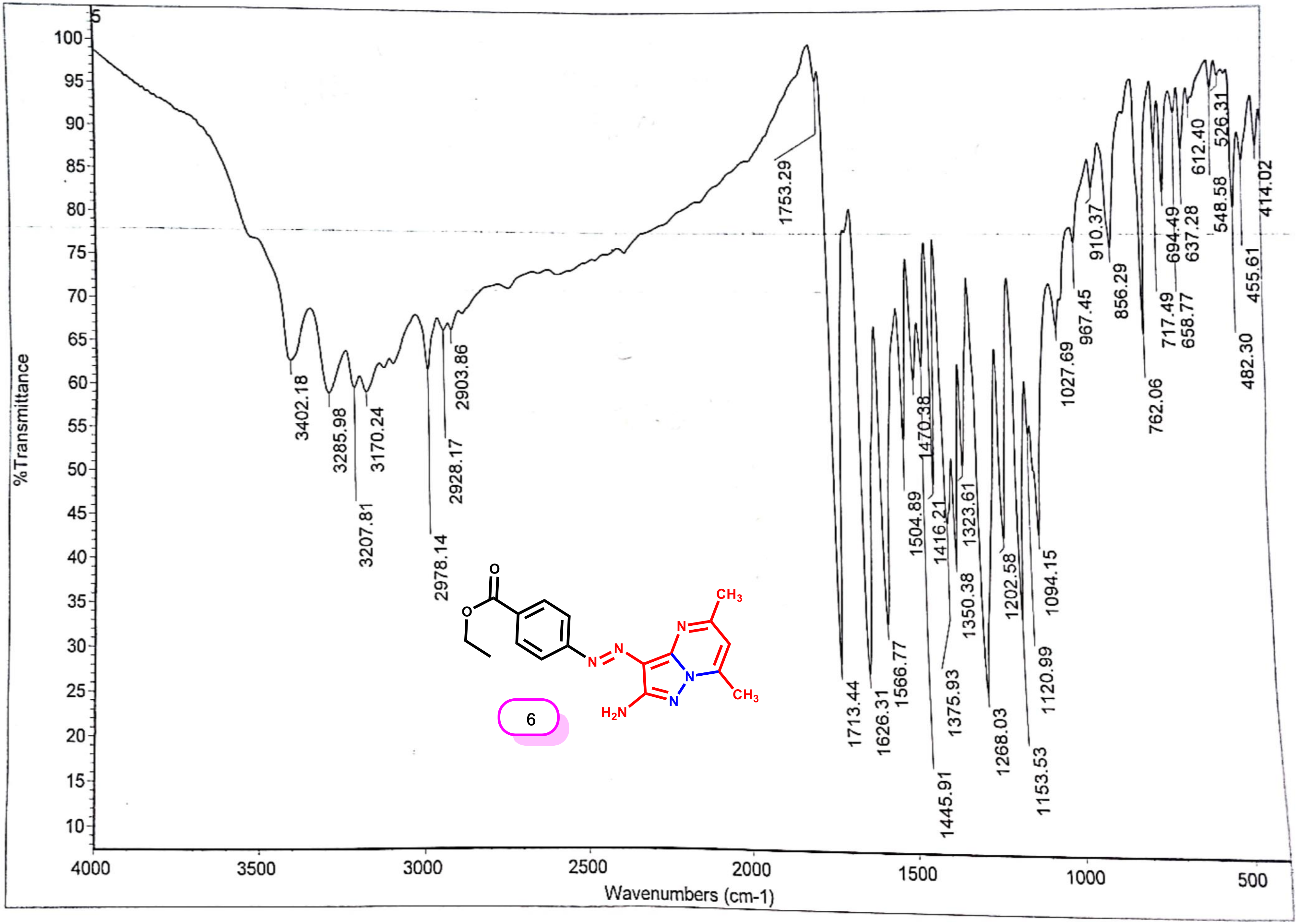

%Transmittance
Wavenumbers (cm-1)
3402.18
3285.98
3207.81
3170.24
2978.14
2928.17
2903.86
1753.29
1713.44
1626.31
1566.77
1504.89
1470.38
1445.91
1416.21
1375.93
1350.38
1323.61
1268.03
1202.58
1153.53
1120.99
1094.15
1027.69
967.45
910.37
856.29
762.06
717.49
694.49
658.77
637.28
612.40
548.58
526.31
482.30
455.61
414.02
O
O
N
N
N
N
N
CH_3
CH_3
H_2N
6

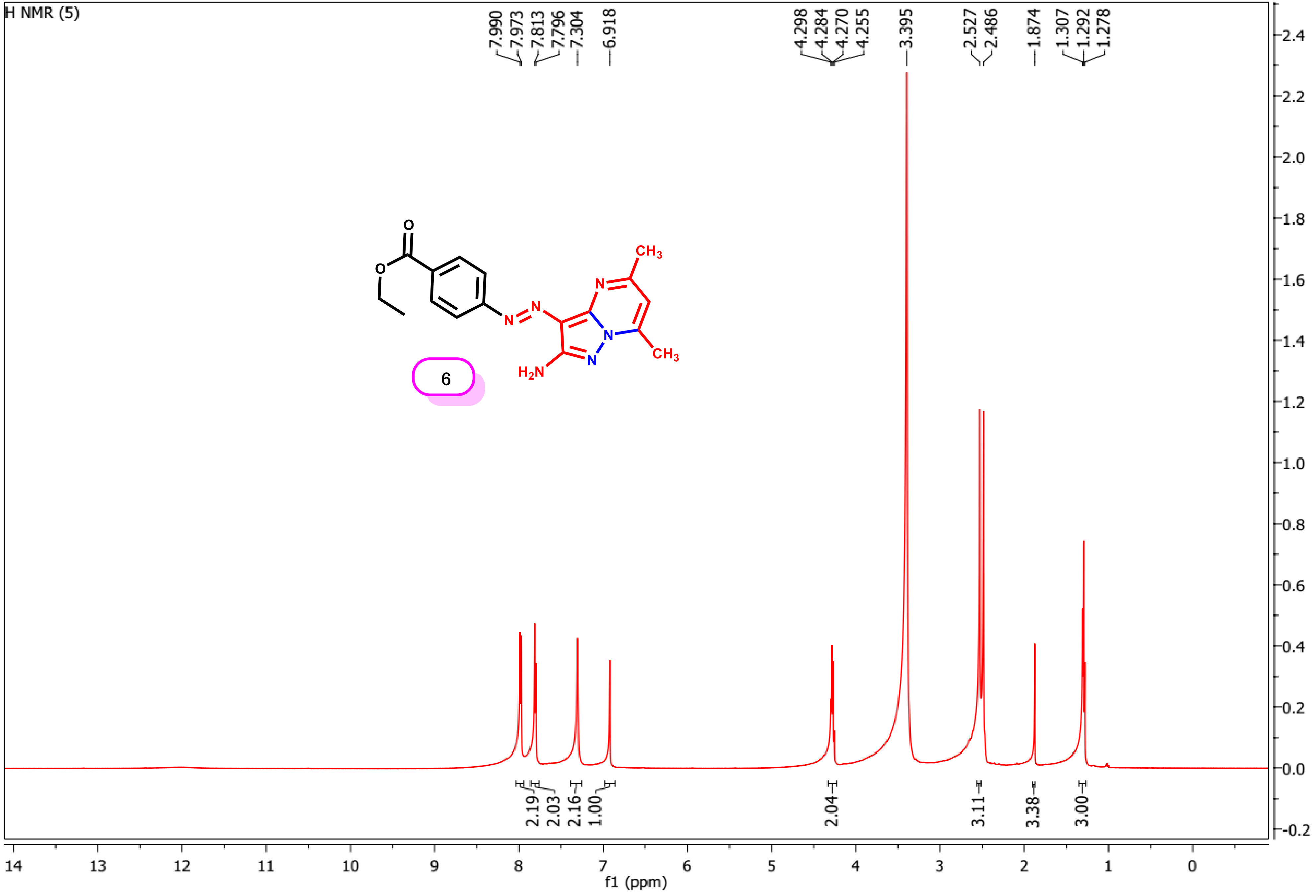

H NMR (5)
7.990
7.973
7.813
7.796
7.304
6.918
4.298
4.284
4.270
4.255
3.395
2.527
2.486
1.874
1.307
1.292
1.278
O
O
N
N
N
N
N
CH3
CH3
H2N
6
2.19
2.03
2.16
1.00
2.04
3.11
3.38
3.00
f1 (ppm)

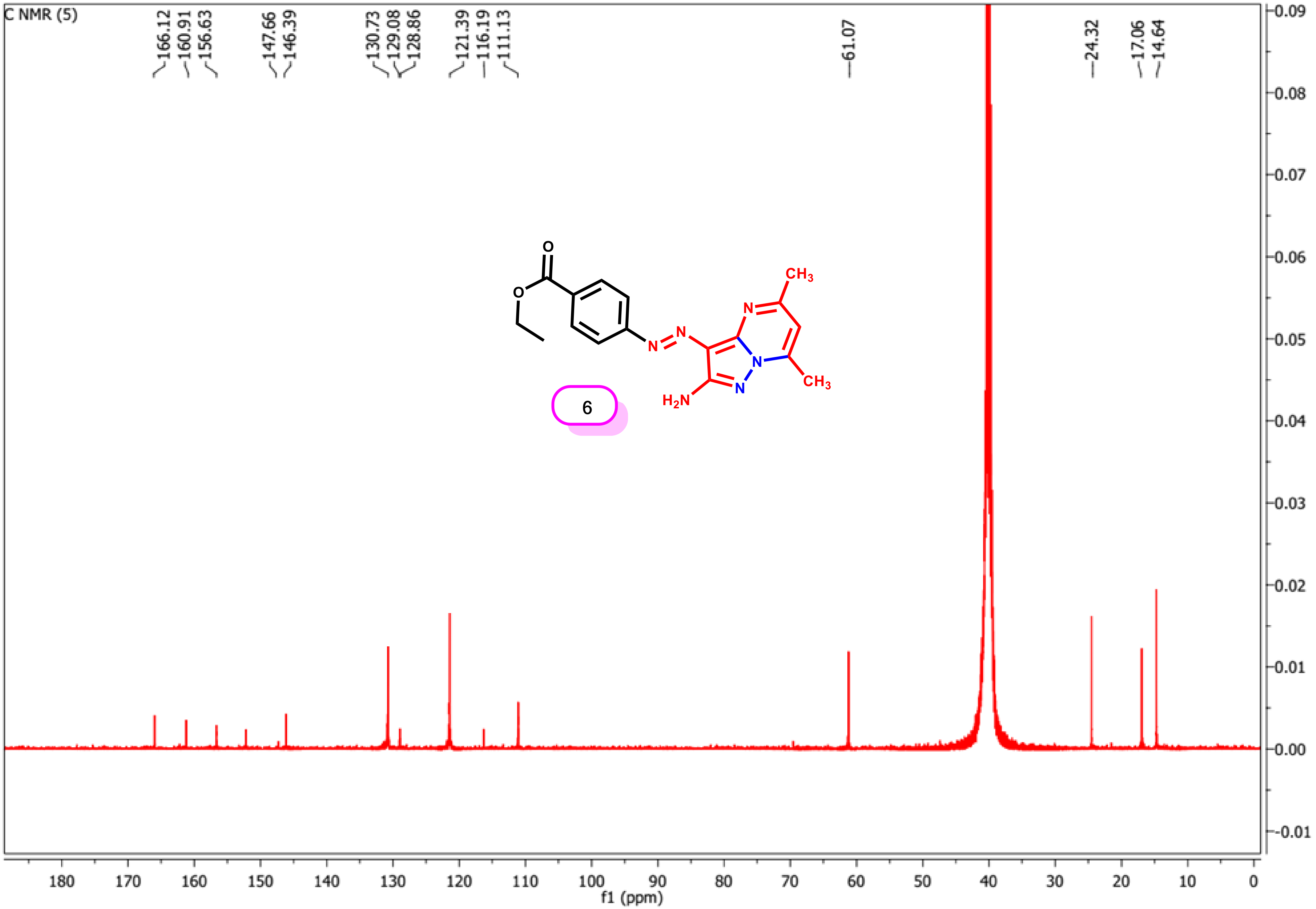
C NMR (5)
166.12
160.91
156.63
147.66
146.39
130.73
129.08
128.86
121.39
116.19
111.13
61.07
24.32
17.06
14.64
O
O
N
N
N
N
N
CH3
CH3
H2N
6
f1 (ppm)

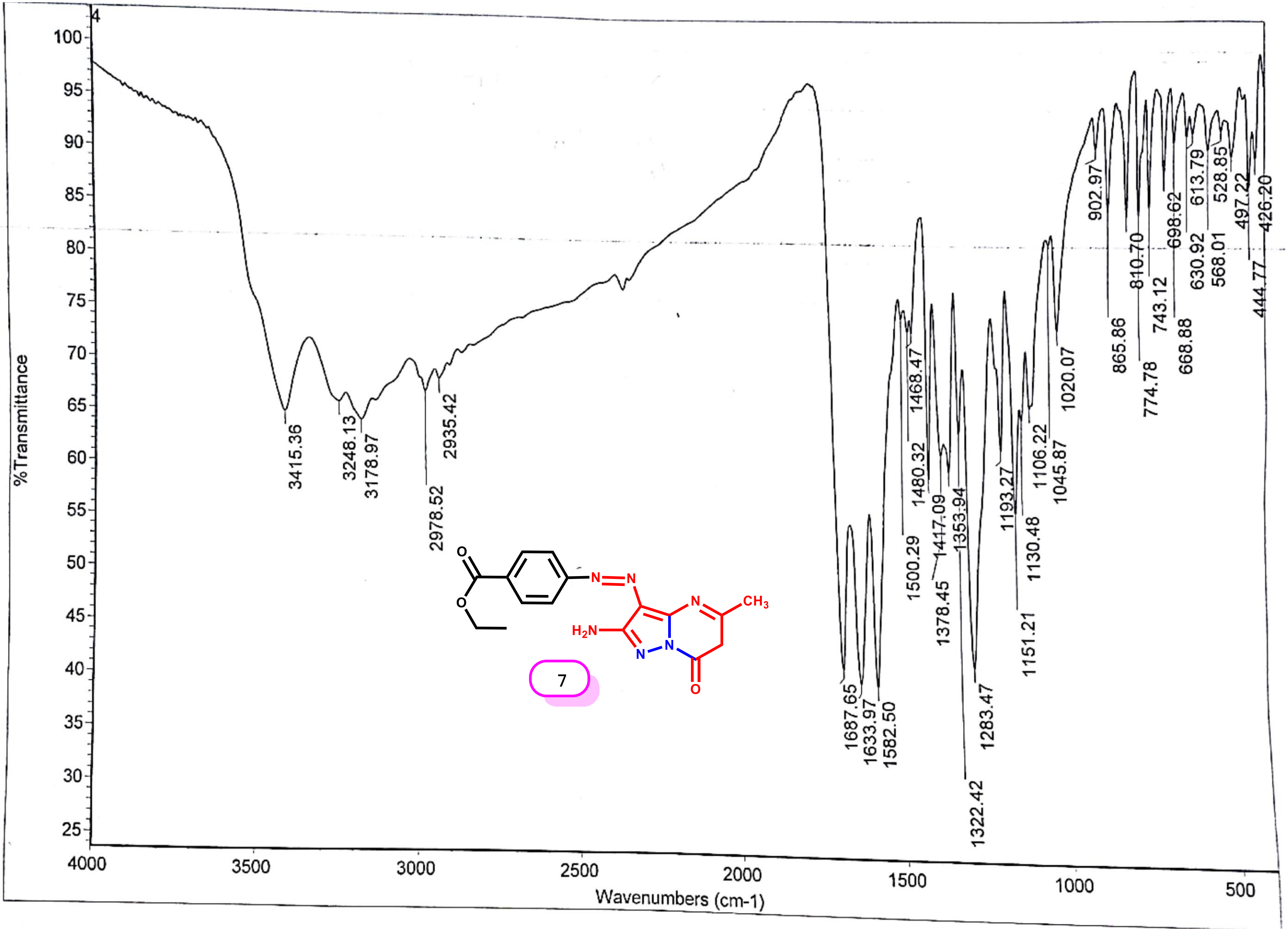
%Transmittance
Wavenumbers (cm-1)
100
95
90
85
80
75
70
65
60
55
50
45
40
35
30
25
4000
3500
3000
2500
2000
1500
1000
500
3415.36
3248.13
3178.97
2978.52
2935.42
1687.65
1633.97
1582.50
1500.29
1480.32
1468.47
1417.09
1378.45
1353.94
1322.42
1283.47
1193.27
1151.21
1130.48
1106.22
1045.87
1020.07
902.97
865.86
810.70
774.78
743.12
698.62
668.88
630.92
613.79
568.01
528.85
497.22
444.77
426.20
O
O
N
N
N
CH3
H2N
N
N
O
7

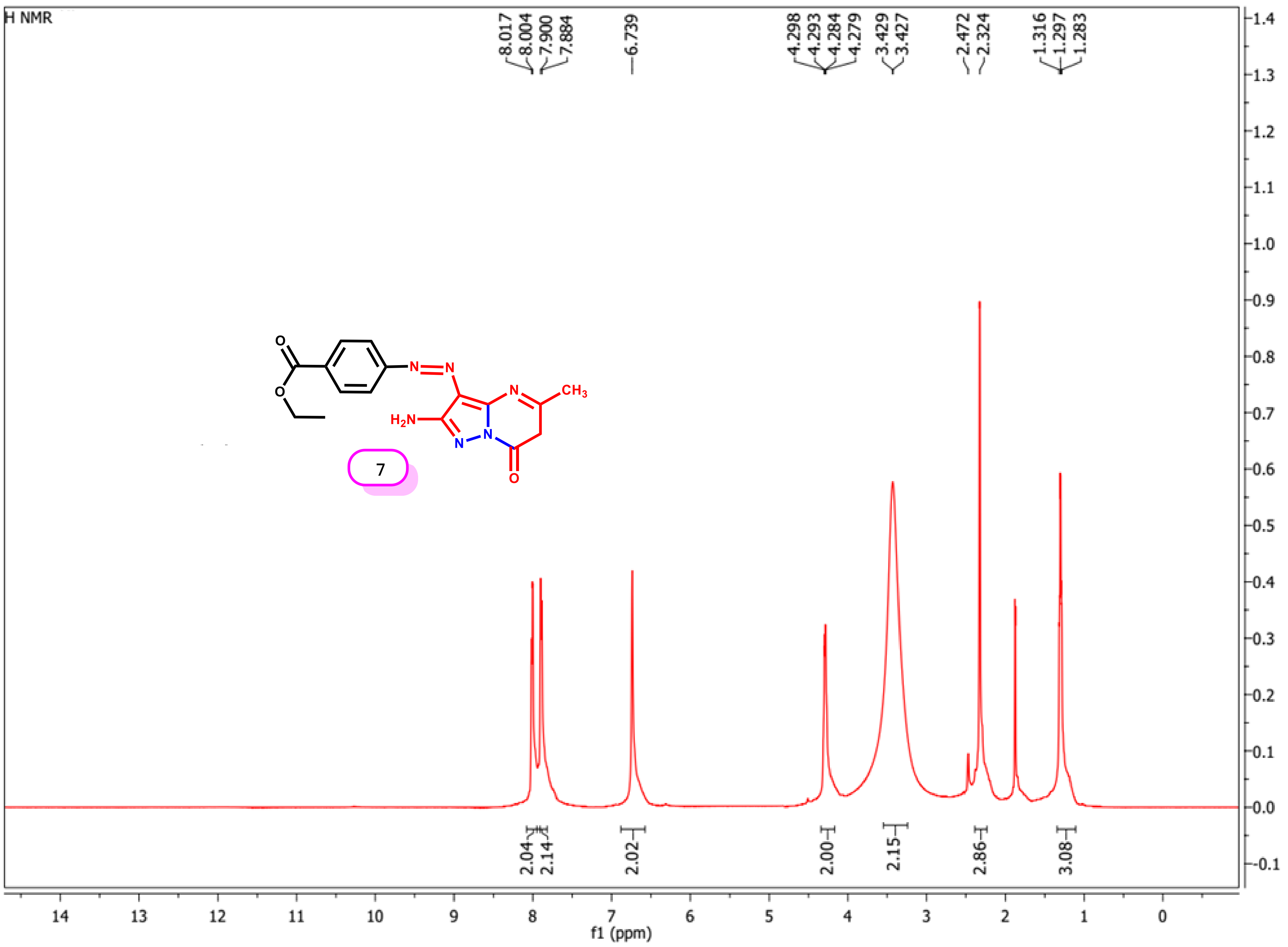
H NMR
8.017
8.004
7.900
7.884
6.739
4.298
4.293
4.284
4.279
3.429
3.427
2.472
2.324
1.316
1.297
1.283
2.04
2.14
2.02
2.00
2.15
2.86
3.08
f1 (ppm)
O
O
N
N
N
N
N
H_2N
CH_3
7

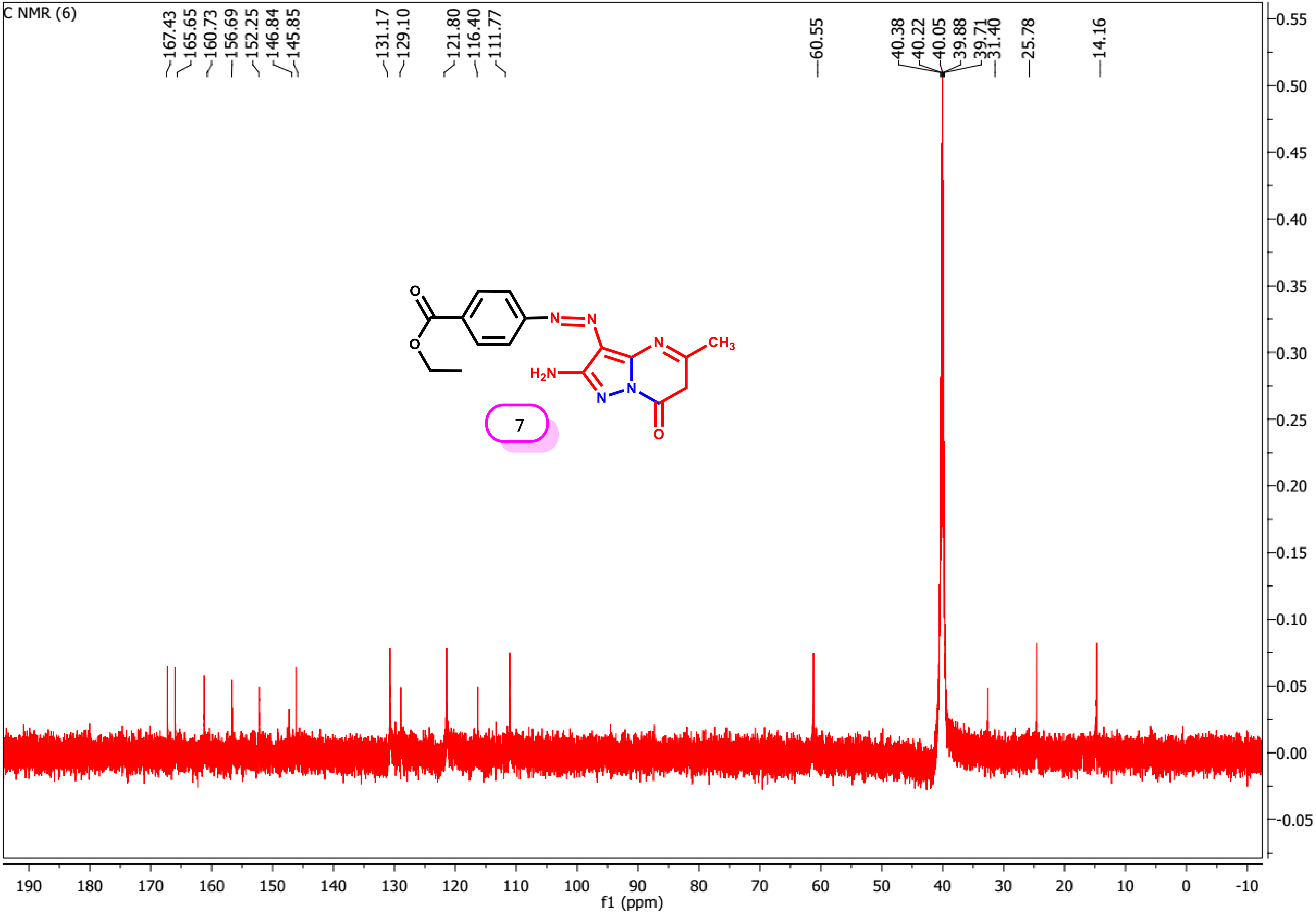

C NMR (6)
167.43
165.65
160.73
156.69
152.25
146.84
145.85
131.17
129.10
121.80
116.40
111.77
60.55
40.38
40.22
40.05
39.88
39.71
31.40
25.78
14.16
7
f1 (ppm)

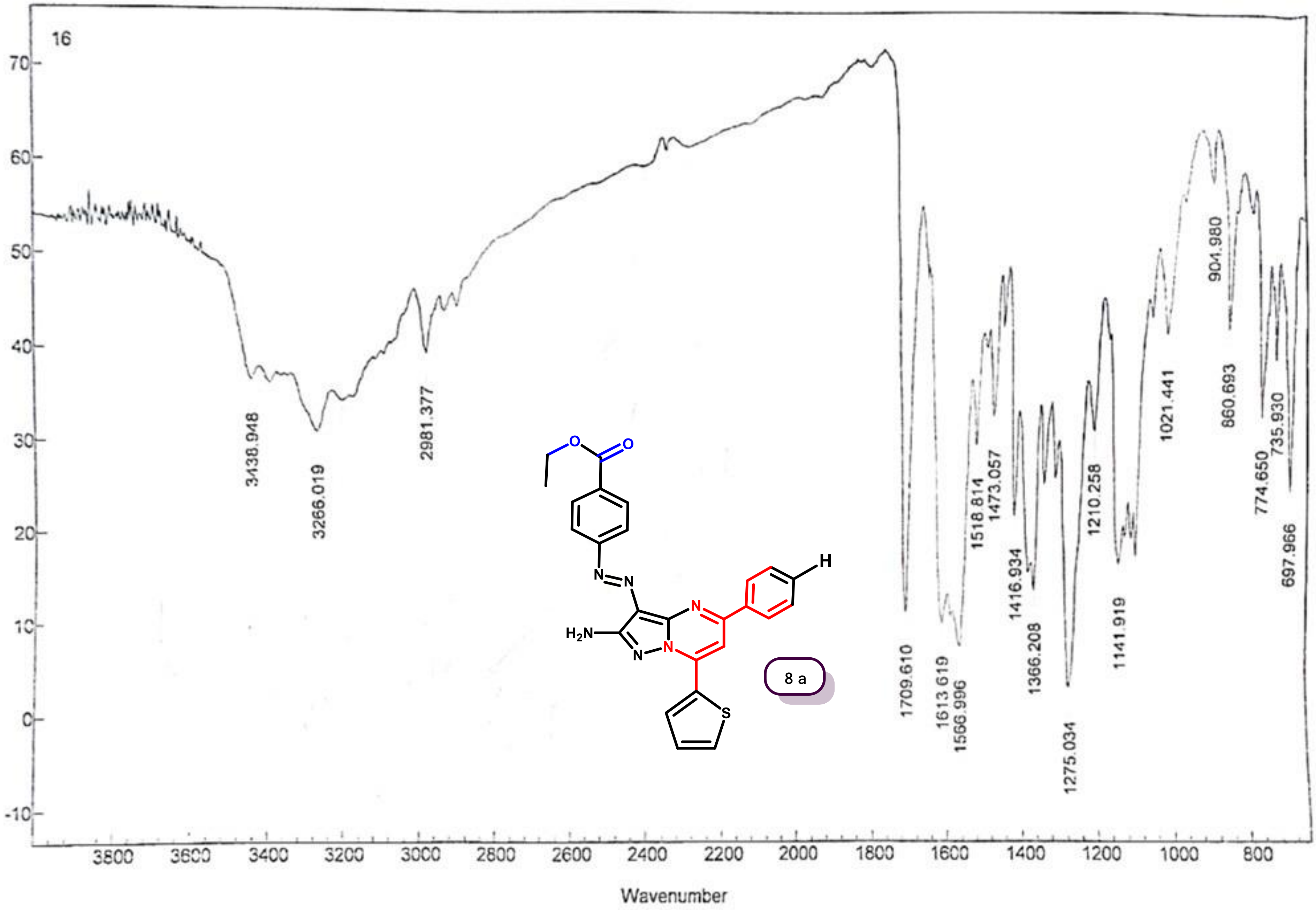
16
Wavenumber
3438.948
3266.019
2981.377
1709.610
1613.619
1566.996
1518.814
1473.057
1416.934
1366.208
1275.034
1210.258
1141.919
1021.441
904.980
860.693
774.650
735.930
697.966
8 a
H$_2$N
S
H

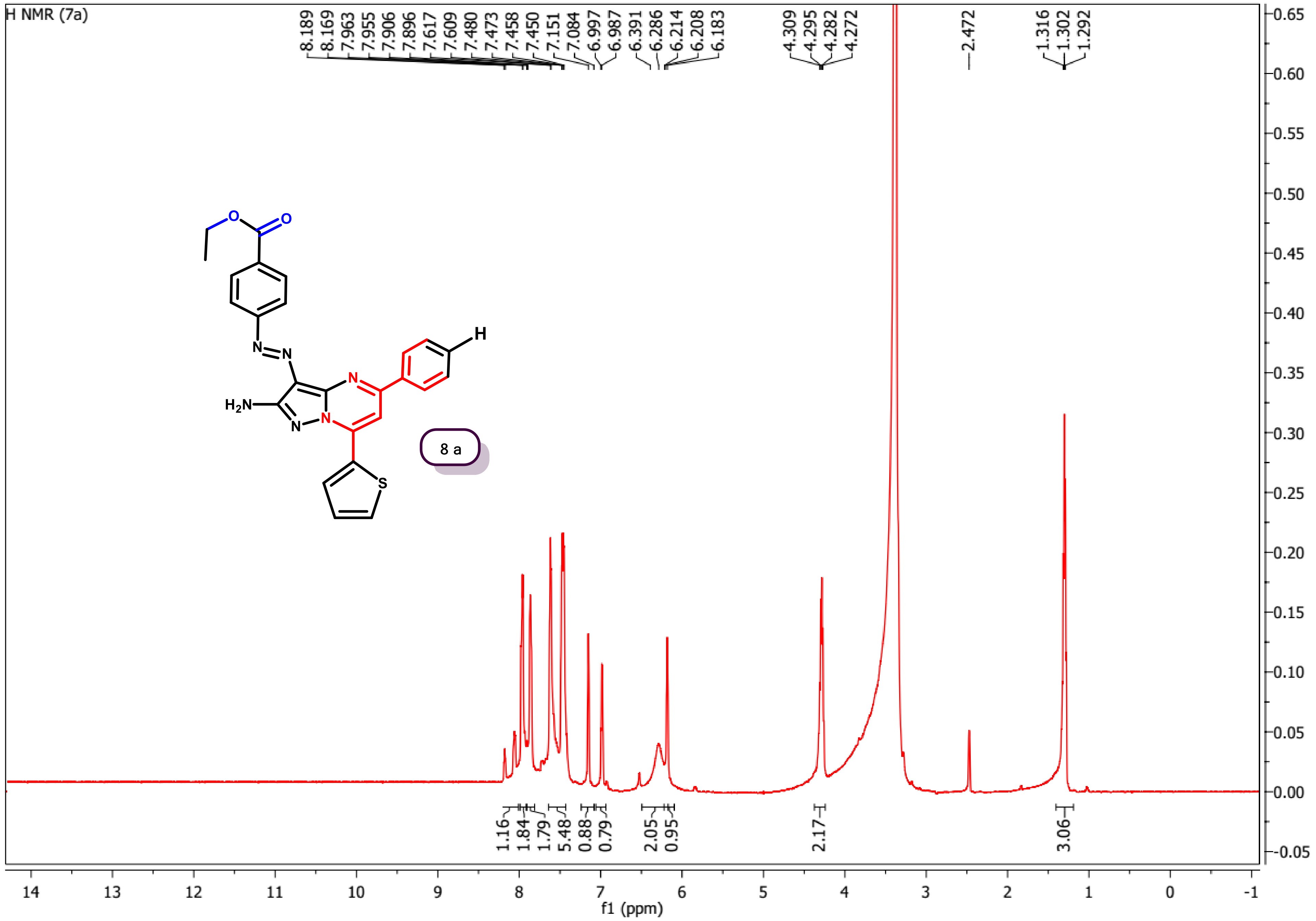
H NMR (7a)
8.189
8.169
7.963
7.955
7.906
7.896
7.617
7.609
7.480
7.473
7.458
7.450
7.151
7.084
6.997
6.987
6.391
6.286
6.214
6.208
6.183
4.309
4.295
4.282
4.272
2.472
1.316
1.302
1.292
H
H2N
N
S
O
8 a
1.16
1.84
1.79
5.48
0.88
0.79
2.05
0.95
2.17
3.06
f1 (ppm)

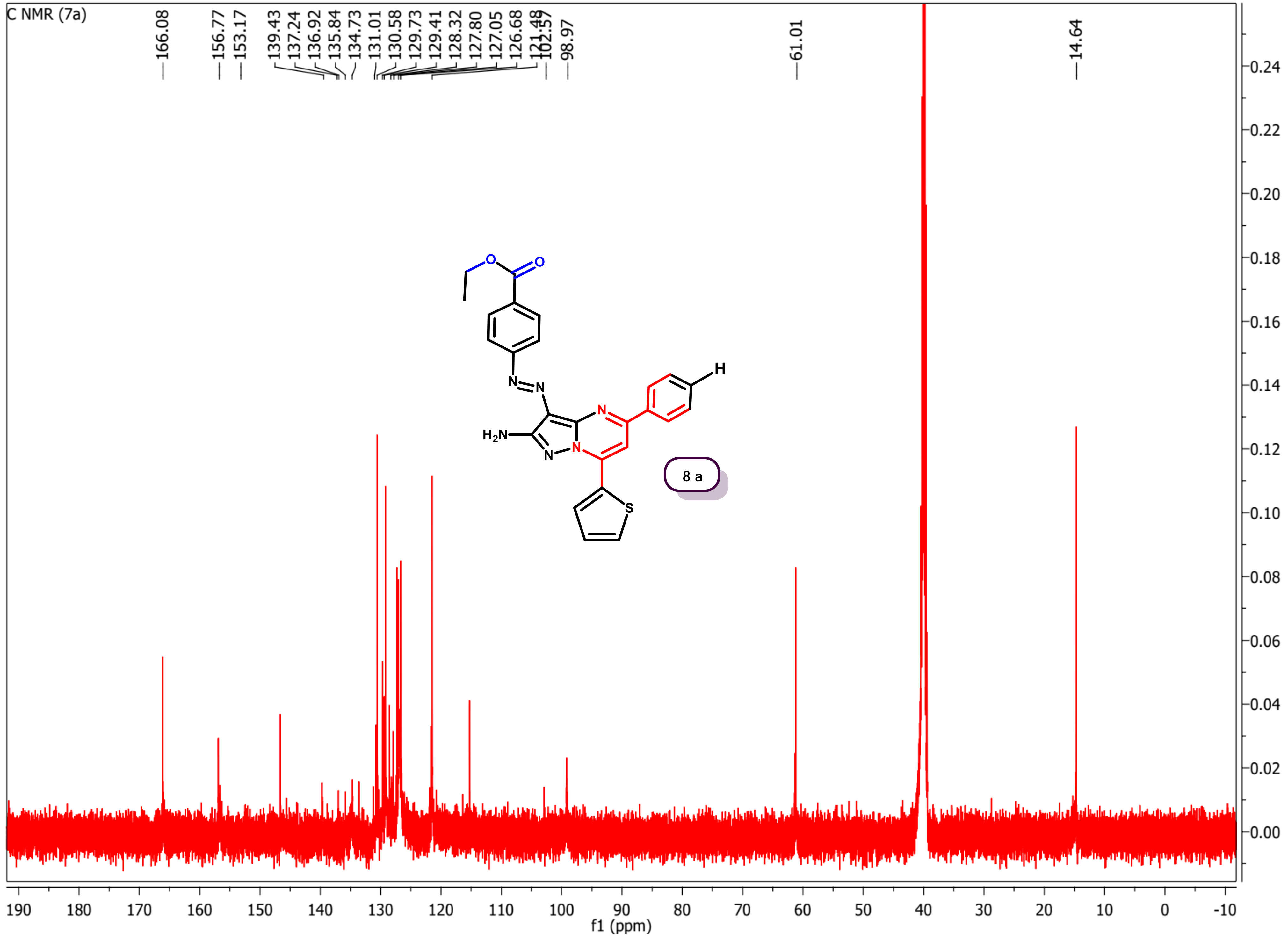
C NMR (7a)
166.08
156.77
153.17
139.43
137.24
136.92
135.84
134.73
131.01
130.58
129.73
129.41
128.32
127.80
127.05
126.68
121.48
102.57
98.97
61.01
14.64
H
H$_2$N
N
N
N
N
N
O
O
S
8 a
f1 (ppm)

t Resolutions Pro

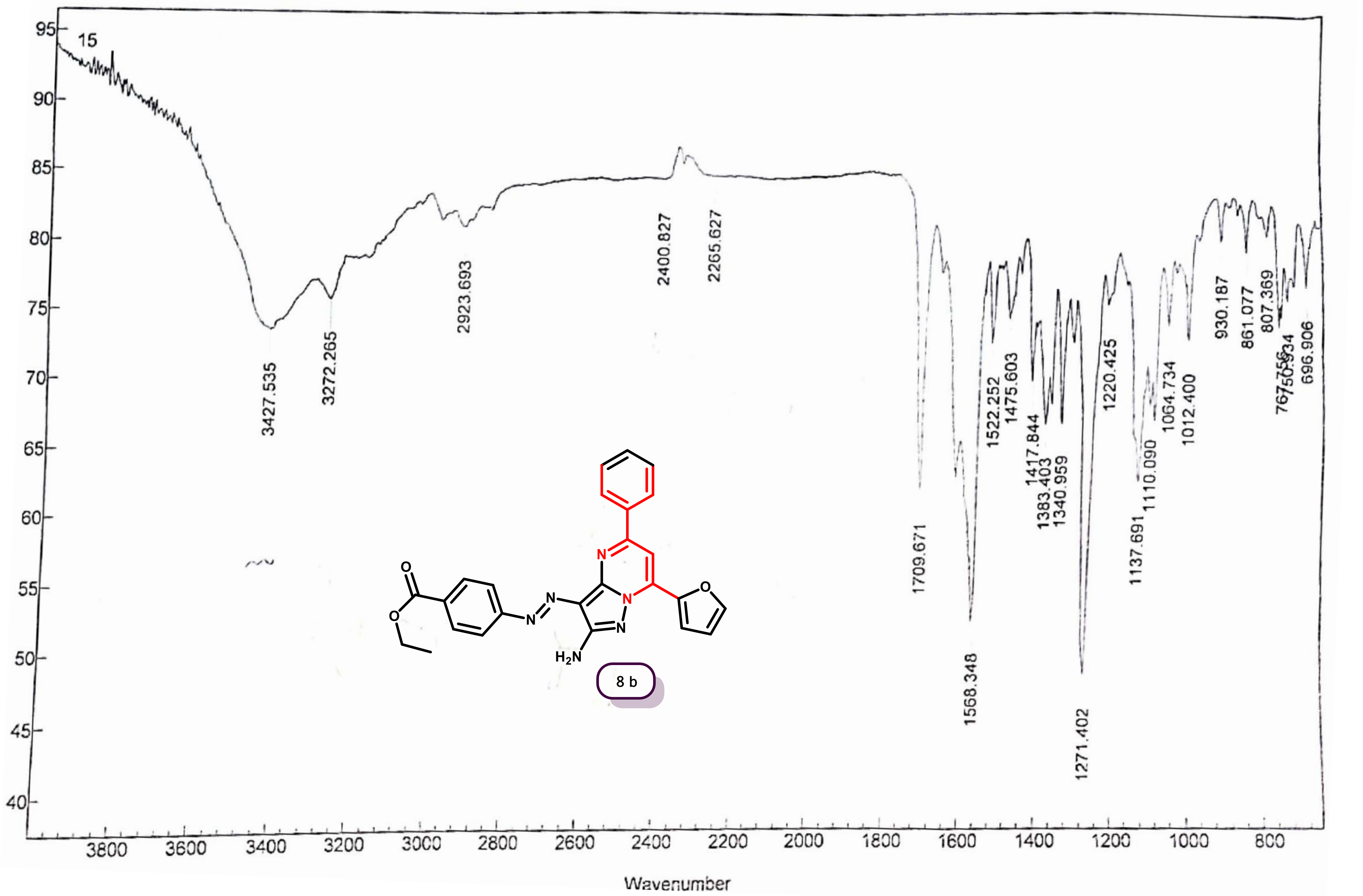

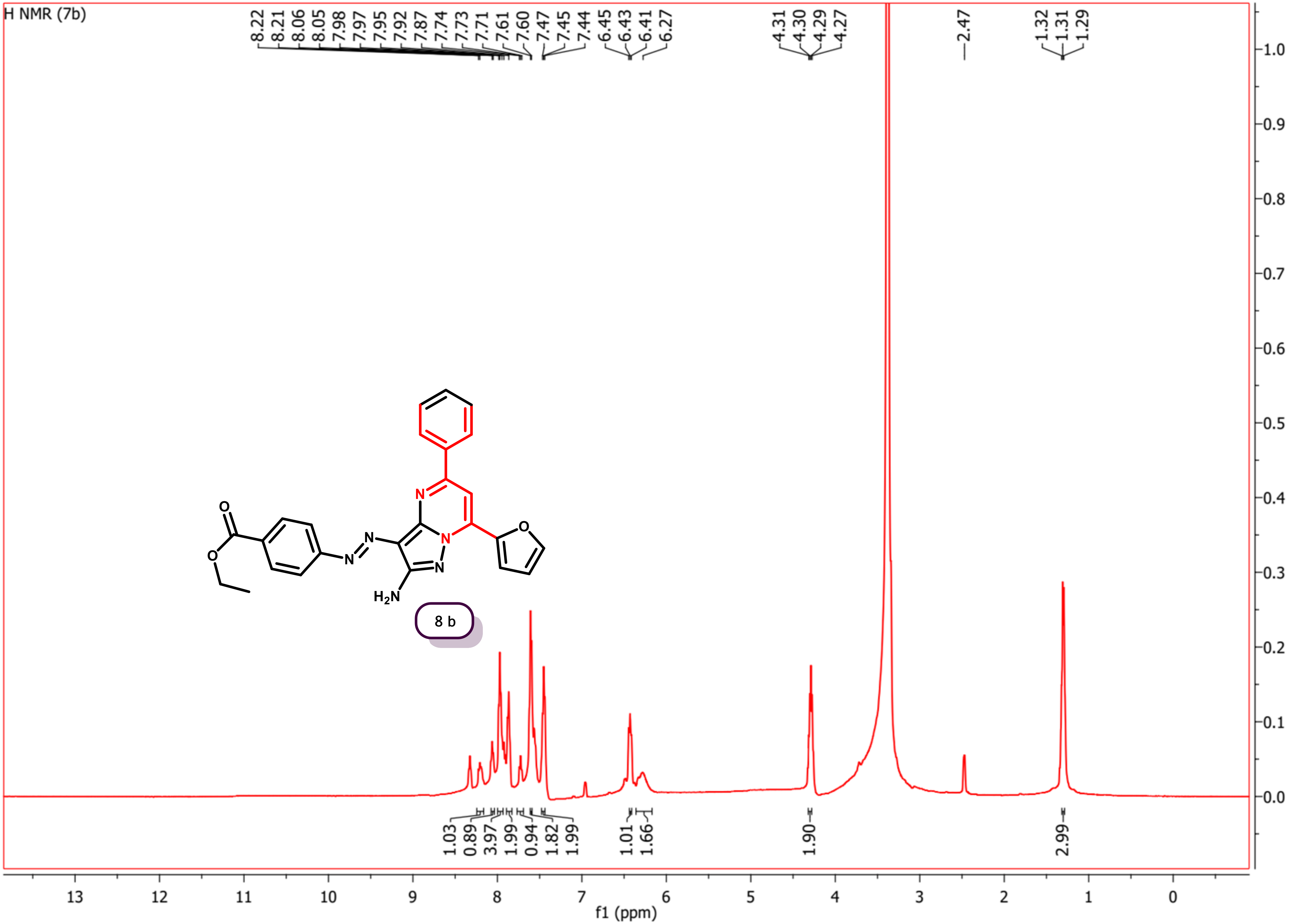
H NMR (7b)
8.22 8.21 8.06 8.05 7.98 7.97 7.95 7.92 7.87 7.74 7.73 7.71 7.61 7.60 7.47 7.45 7.44 6.45 6.43 6.41 6.27
4.31 4.30 4.29 4.27
2.47
1.32 1.31 1.29
O
O
N
N
N
N
N
O
H2N
8 b
1.03 0.89 3.97 1.99 0.94 1.82 1.99
1.01 1.66
1.90
2.99
13 12 11 10 9 8 7 6 5 4 3 2 1 0
f1 (ppm)
1.0 0.9 0.8 0.7 0.6 0.5 0.4 0.3 0.2 0.1 0.0

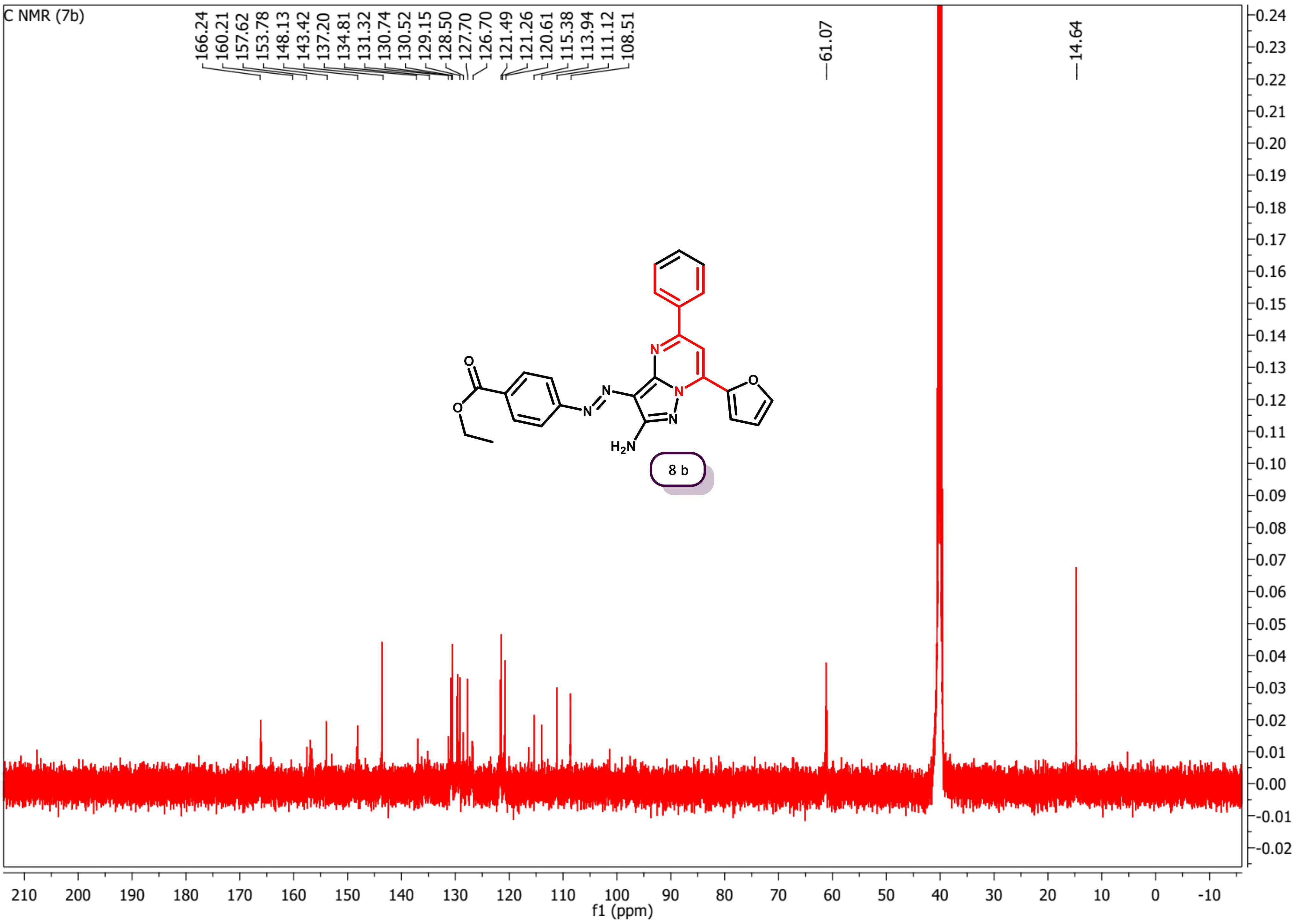
C NMR (7b)
166.24
160.21
157.62
153.78
148.13
143.42
137.20
134.81
131.32
130.74
130.52
129.15
128.50
127.70
126.70
121.49
121.26
120.61
115.38
113.94
111.12
108.51
61.07
14.64
8 b
H2N
f1 (ppm)

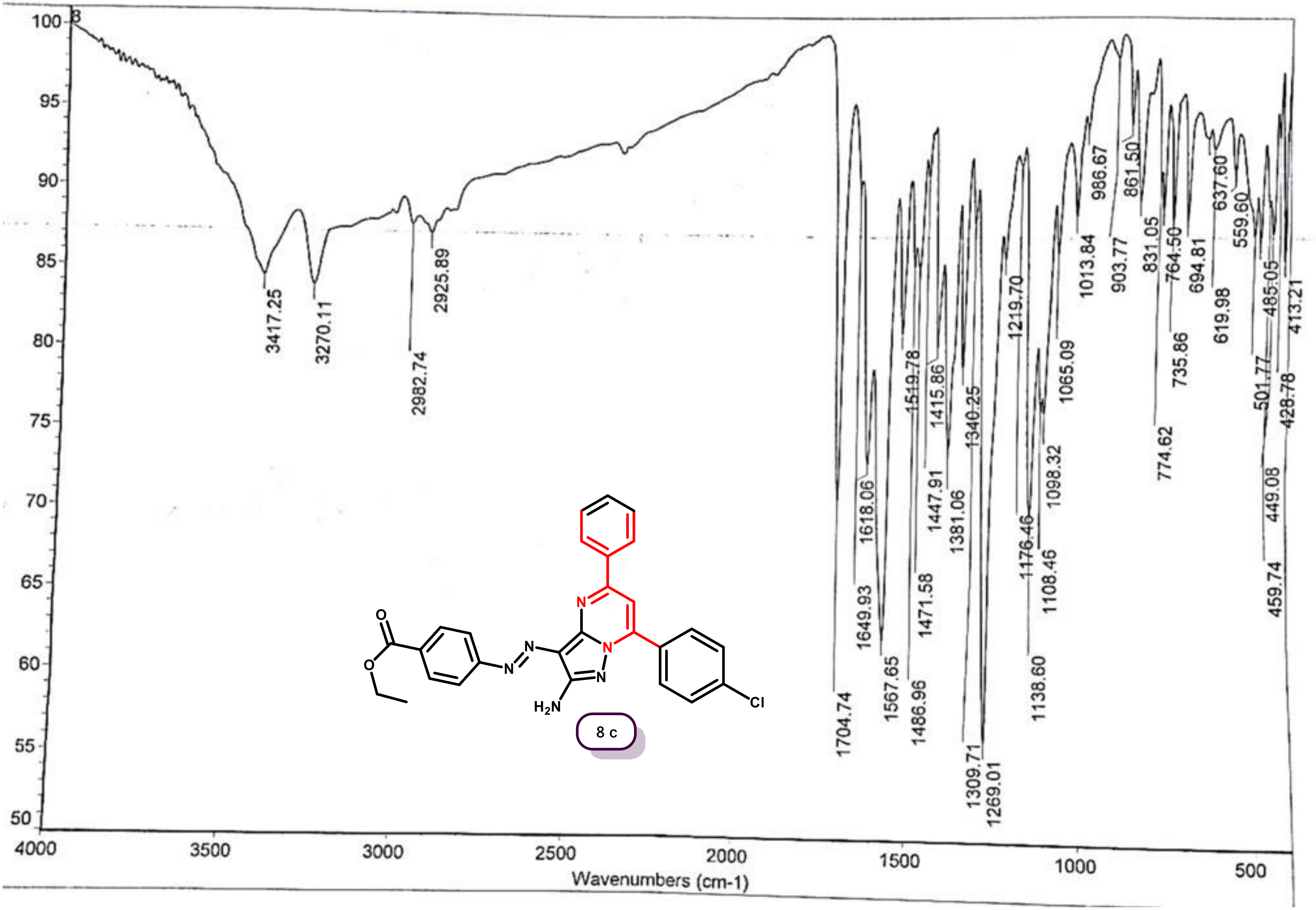

Wavenumbers (cm-1)
4000
3500
3000
2500
2000
1500
1000
500
100
95
90
85
80
75
70
65
60
55
50
3417.25
3270.11
2982.74
2925.89
1704.74
1649.93
1618.06
1567.65
1519.78
1486.96
1471.58
1447.91
1415.86
1381.06
1340.25
1309.71
1269.01
1219.70
1176.46
1138.60
1108.46
1098.32
1065.09
1013.84
986.67
903.77
861.50
831.05
774.62
764.50
735.86
694.81
637.60
619.98
559.60
501.77
485.05
459.74
449.08
428.78
413.21
O
O
N
N
N
N
N
H2N
Cl
8 c

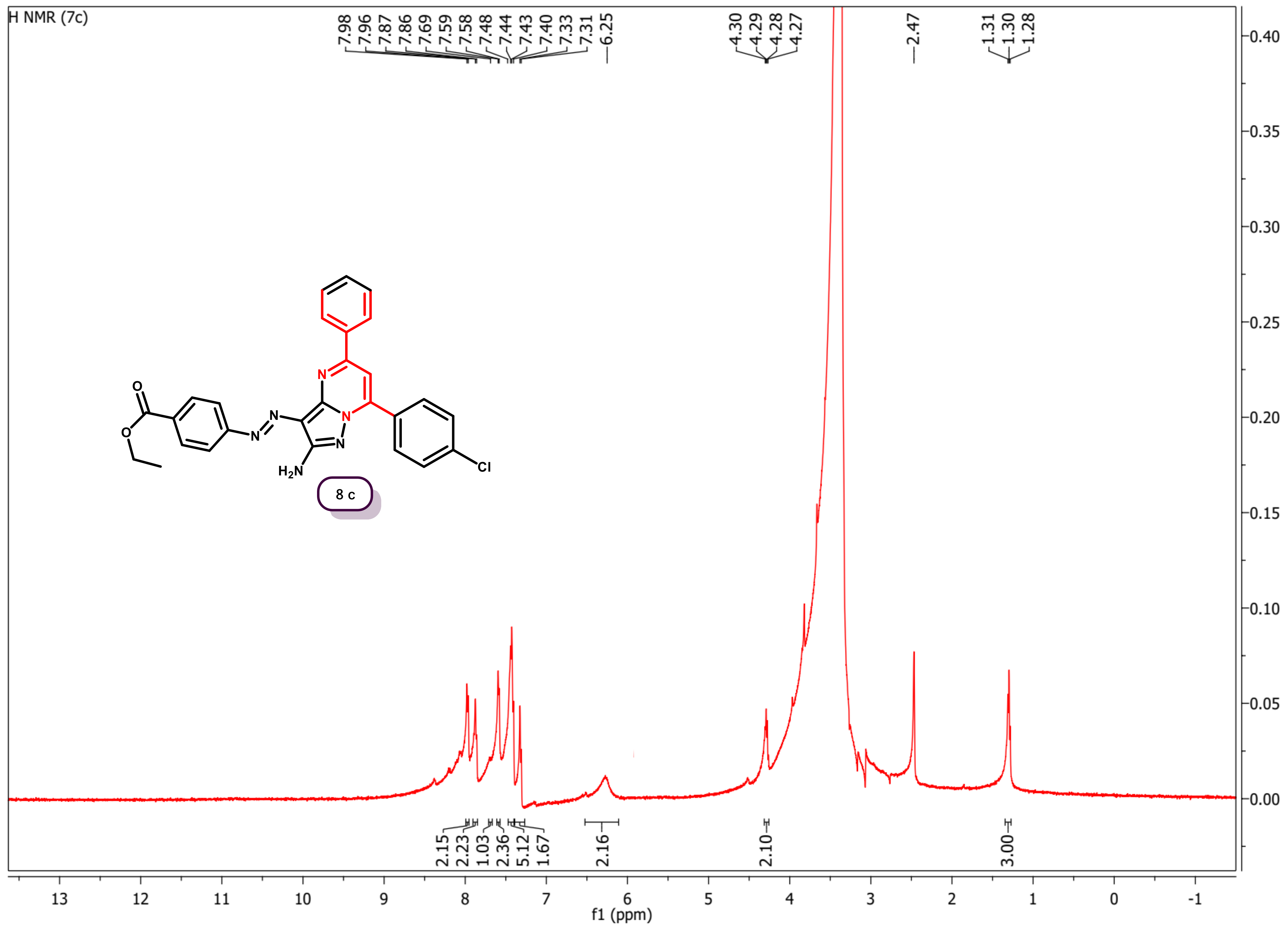
H NMR (7c)
7.98
7.96
7.87
7.86
7.69
7.59
7.58
7.48
7.44
7.43
7.40
7.33
7.31
6.25
4.30
4.29
4.28
4.27
2.47
1.31
1.30
1.28
8 c
2.15
2.23
1.03
2.36
5.12
1.67
2.16
2.10
3.00
f1 (ppm)

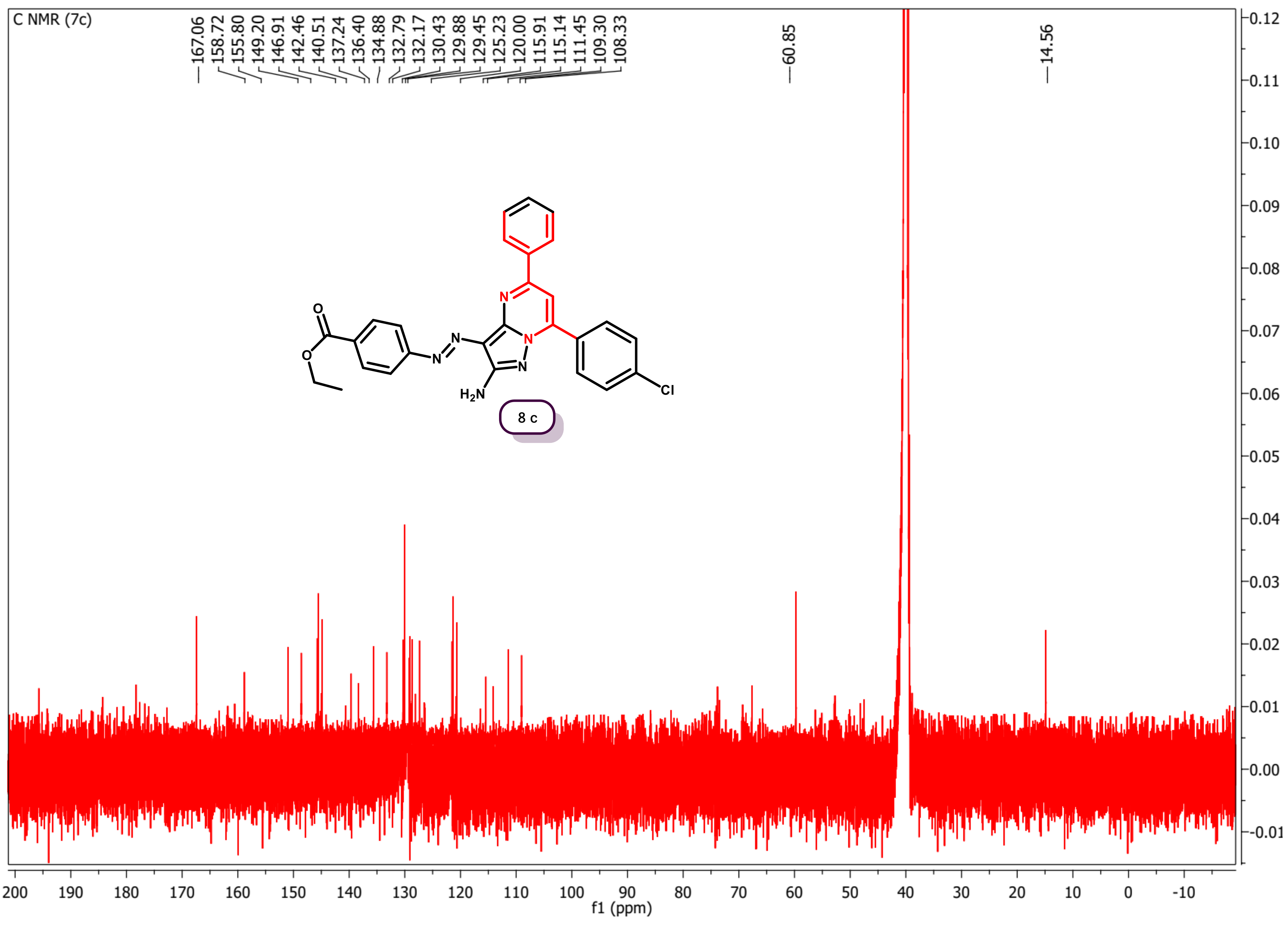
C NMR (7c)
167.06
158.72
155.80
149.20
146.91
142.46
140.51
137.24
136.40
134.88
132.79
132.17
130.43
129.88
129.45
125.23
120.00
115.91
115.14
111.45
109.30
108.33
60.85
14.56
O
O
N
N
N
N
N
H2N
Cl
8 c
f1 (ppm)

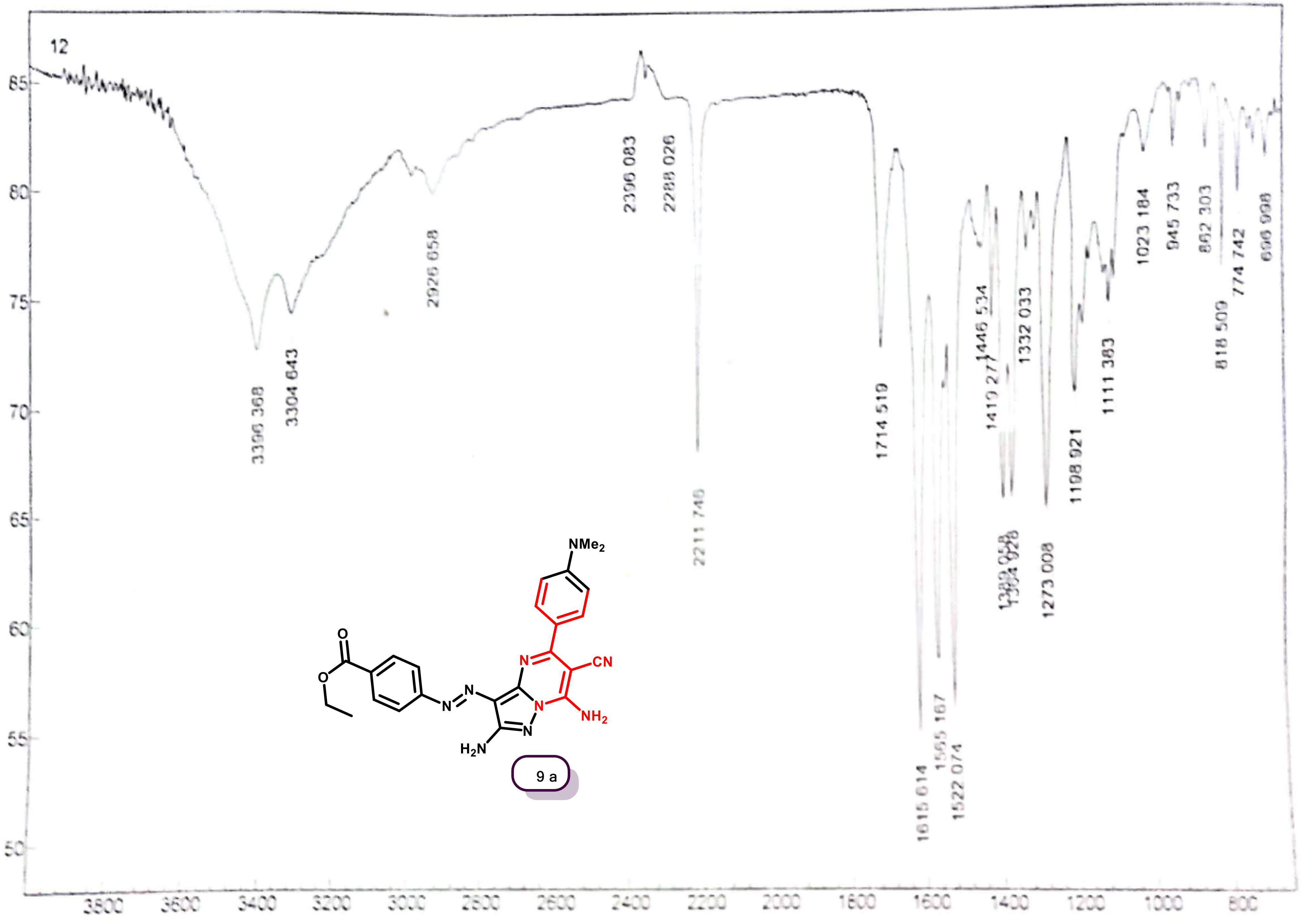

12
85
80
75
70
65
60
55
50
3396 368
3304 643
2926 658
2396 083
2288 026
2211 746
1714 519
1615 614
1565 167
1522 074
1446 534
1419 277
1332 033
1273 008
1198 921
1111 383
1023 184
945 733
862 303
818 509
774 742
696 998
3800
3600
3400
3200
3000
2800
2600
2400
2200
2000
1800
1600
1400
1200
1000
800
NMe2
CN
NH2
H2N
O
N
9 a

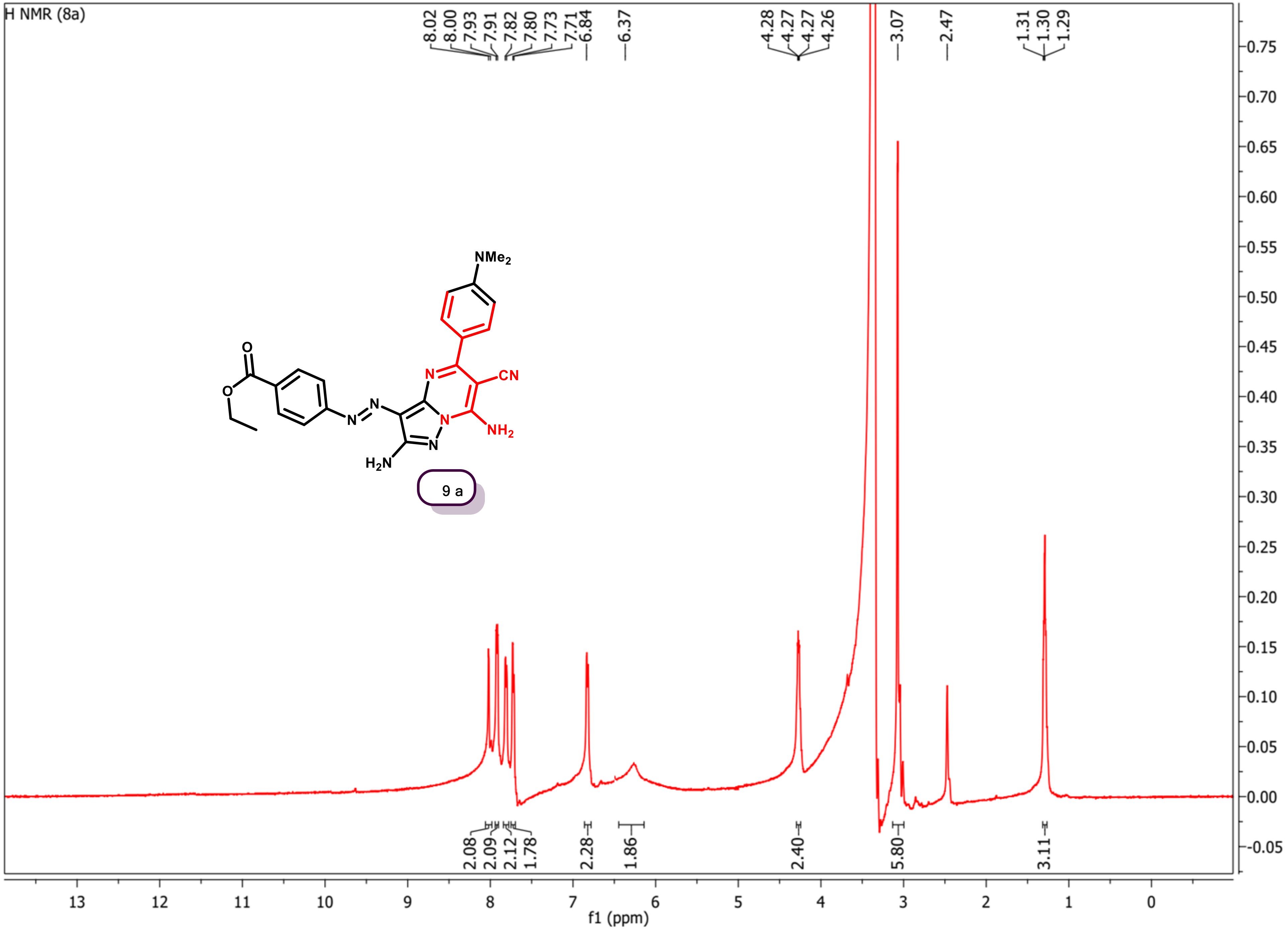
H NMR (8a)
8.02 8.00 7.93 7.91 7.82 7.80 7.73 7.71 6.84 6.37 4.28 4.27 4.27 4.26 3.07 2.47 1.31 1.30 1.29
NMe2
O
O
N
N
N
N
N
CN
NH2
H2N
9 a
2.08 2.09 2.12 1.78 2.28 1.86 2.40 5.80 3.11
f1 (ppm)

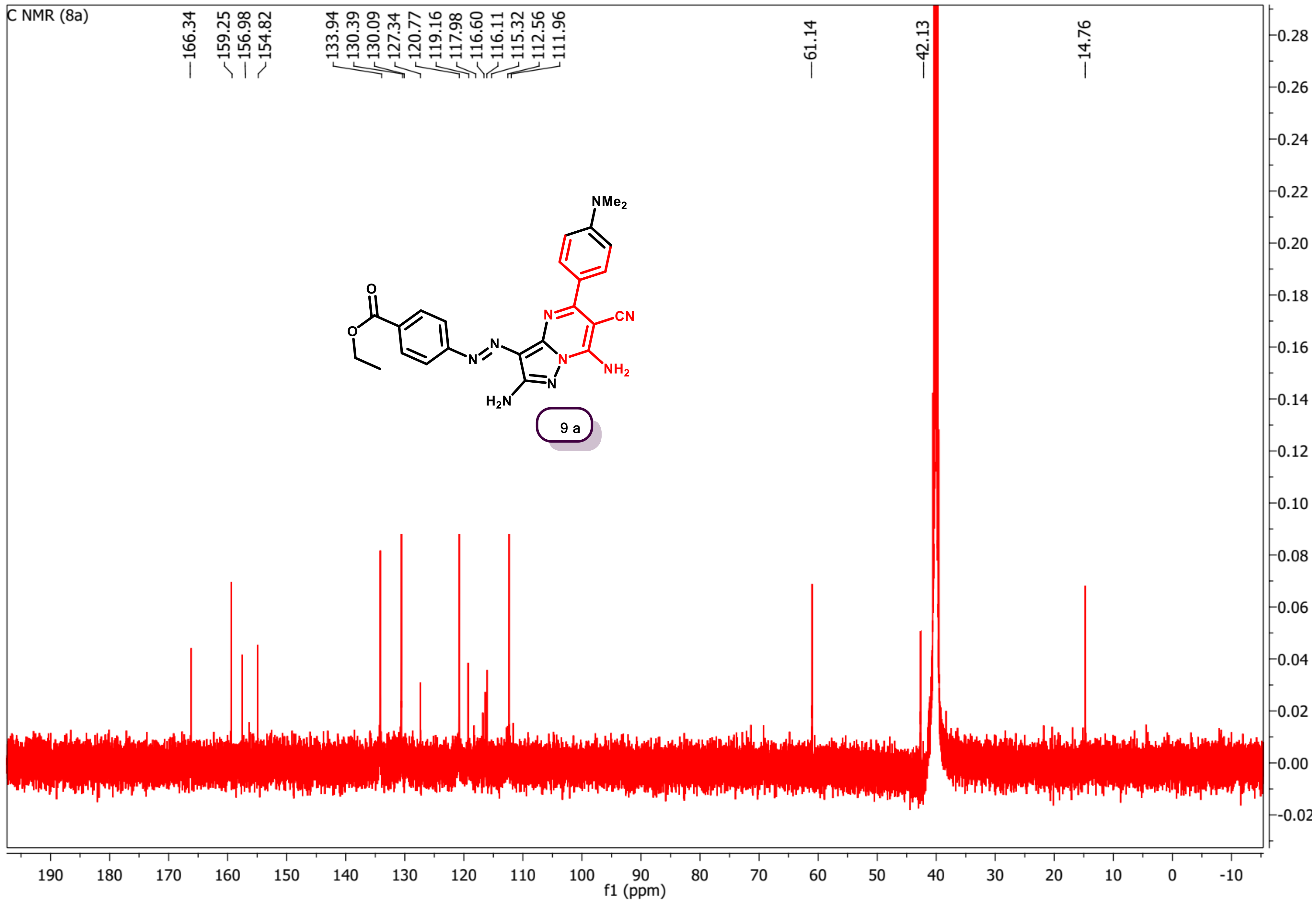
C NMR (8a)
166.34
159.25
156.98
154.82
133.94
130.39
130.09
127.34
120.77
119.16
117.98
116.60
116.11
115.32
112.56
111.96
61.14
42.13
14.76
NMe2
O
O
N
N
N
CN
N
N
NH2
H2N
9 a
f1 (ppm)

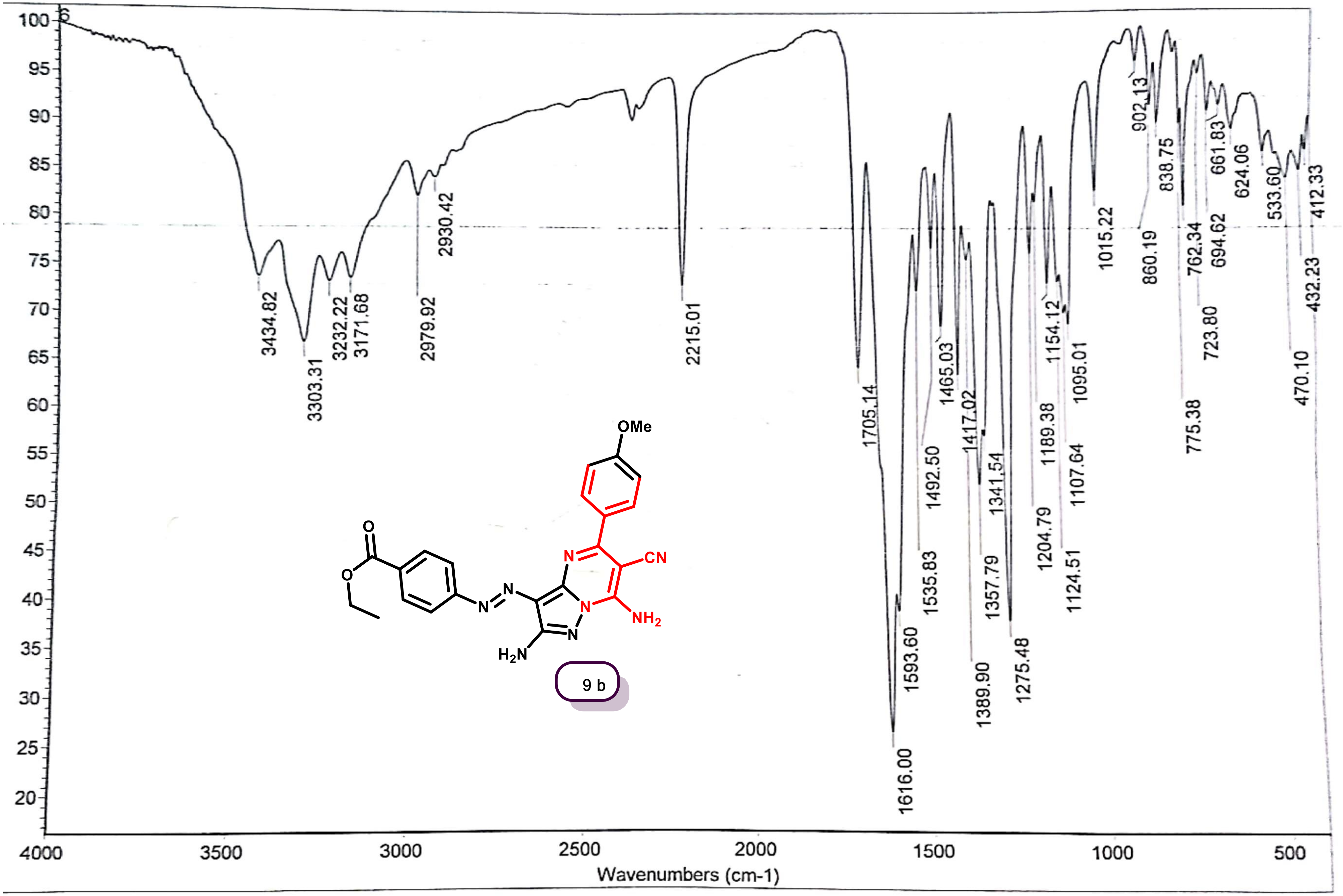

100
95
90
85
80
75
70
65
60
55
50
45
40
35
30
25
20
4000
3500
3000
2500
2000
1500
1000
500
Wavenumbers (cm-1)
3434.82
3303.31
3232.22
3171.68
2979.92
2930.42
2215.01
1705.14
1616.00
1593.60
1535.83
1492.50
1465.03
1417.02
1389.90
1357.79
1341.54
1275.48
1204.79
1189.38
1154.12
1124.51
1107.64
1095.01
1015.22
902.13
860.19
838.75
775.38
762.34
723.80
694.62
661.83
624.06
533.60
470.10
432.23
412.33
OMe
O
O
N
N
N
N
N
CN
NH_2
H_2N
9 b

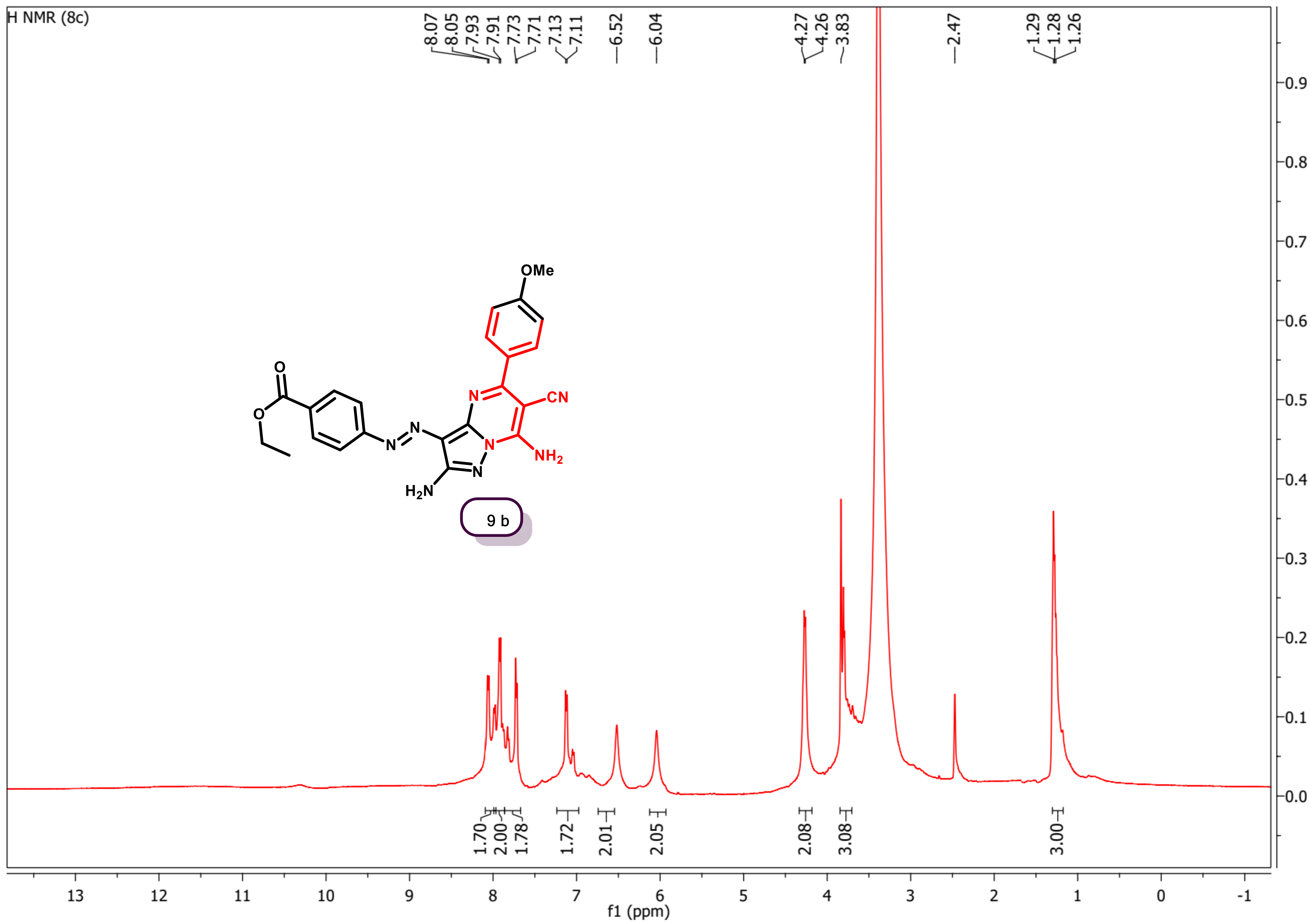

H NMR (8c)
8.07
8.05
7.93
7.91
7.73
7.71
7.13
7.11
6.52
6.04
4.27
4.26
3.83
2.47
1.29
1.28
1.26
OMe
O
O
N
N
N
CN
N
N
NH2
H2N
9 b
1.70
2.00
1.78
1.72
2.01
2.05
2.08
3.08
3.00
13
12
11
10
9
8
7
6
5
4
3
2
1
0
-1
f1 (ppm)
0.9
0.8
0.7
0.6
0.5
0.4
0.3
0.2
0.1
0.0

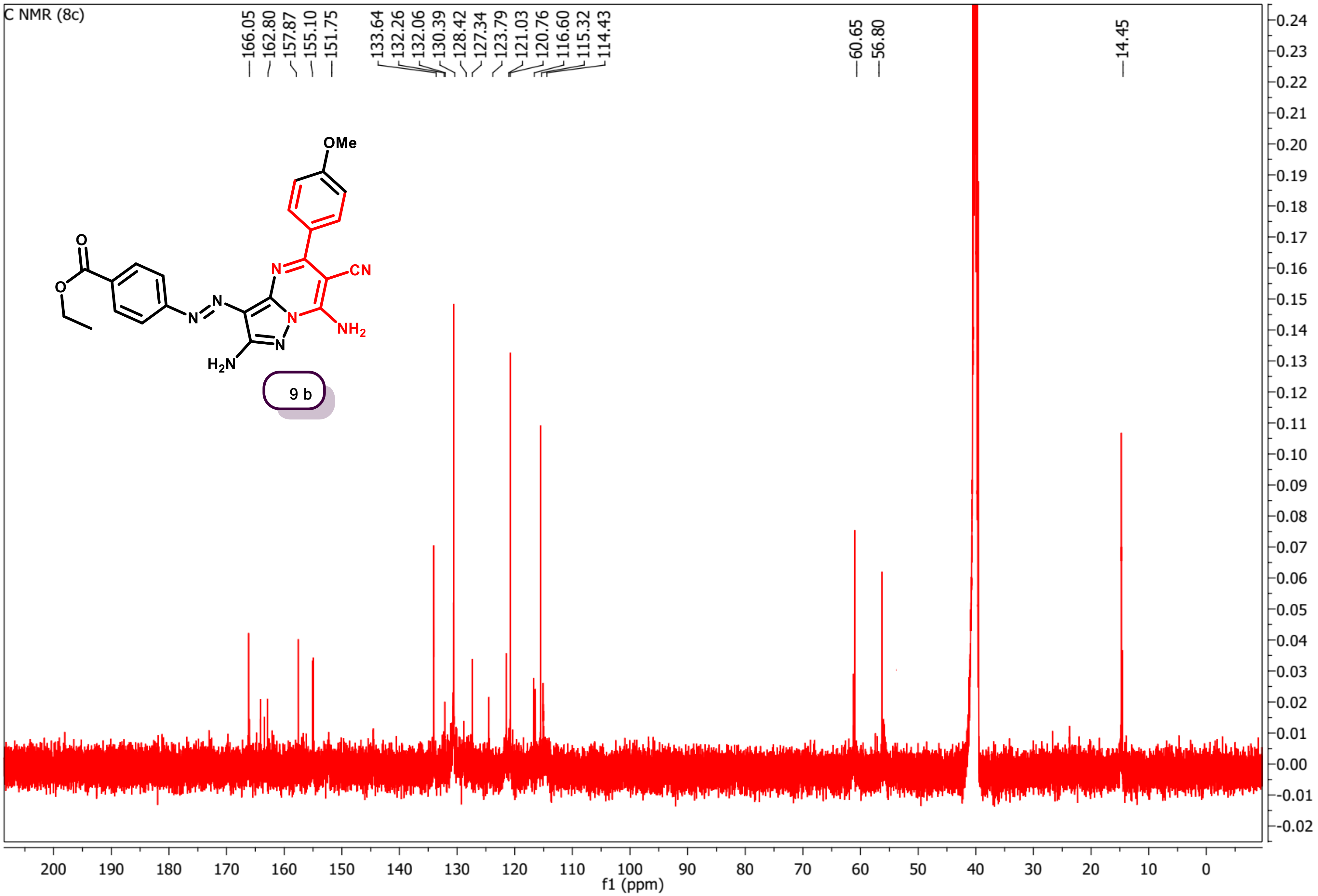
C NMR (8c)
166.05
162.80
157.87
155.10
151.75
133.64
132.26
132.06
130.39
128.42
127.34
123.79
121.03
120.76
116.60
115.32
114.43
60.65
56.80
14.45
OMe
O
O
N
N
N
N
CN
N
NH2
N
H2N
9 b
f1 (ppm)

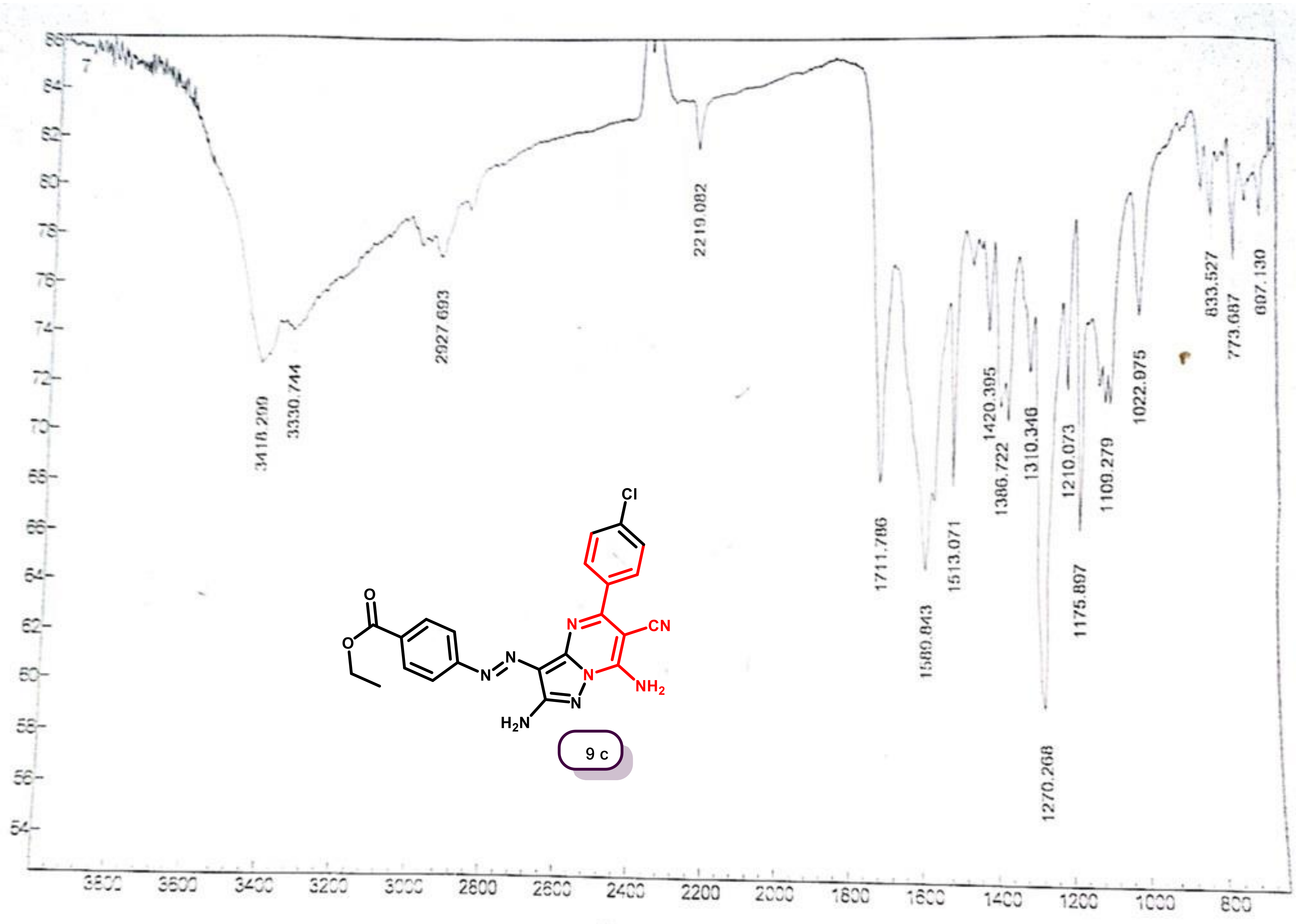

3418.299
3330.744
2927.693
2219.082
1711.786
1589.843
1513.071
1420.395
1386.722
1310.346
1270.268
1210.073
1175.897
1109.279
1022.975
833.527
773.687
697.130
3800
3600
3400
3200
3000
2800
2600
2400
2200
2000
1800
1600
1400
1200
1000
800
Cl
CN
NH2
H2N
9 c

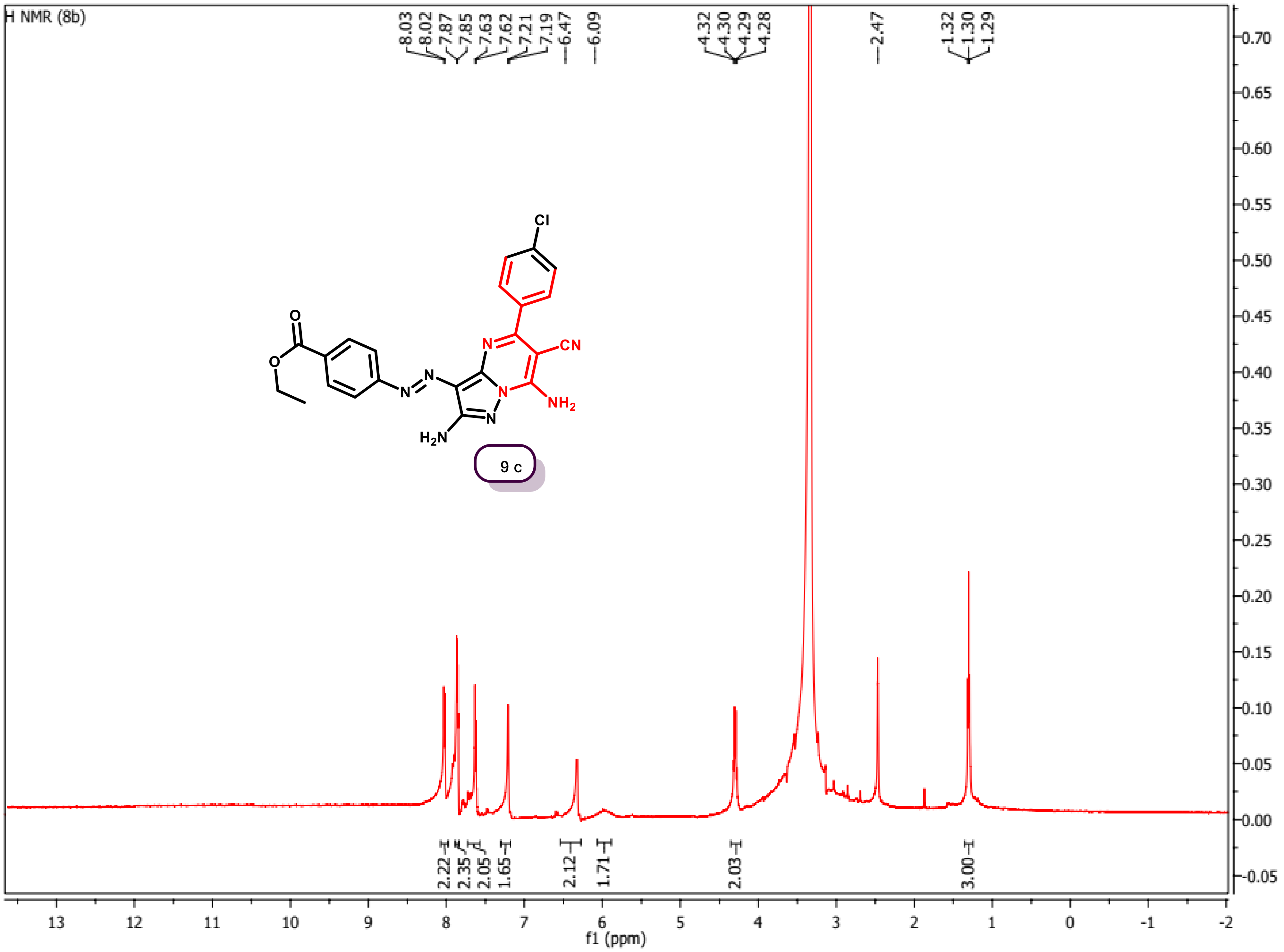
H NMR (8b)
8.03
8.02
7.87
7.85
7.63
7.62
7.21
7.19
6.47
6.09
4.32
4.30
4.29
4.28
2.47
1.32
1.30
1.29
2.22
2.35
2.05
1.65
2.12
1.71
2.03
3.00
Cl
CN
NH2
H2N
N
O
9 c
f1 (ppm)

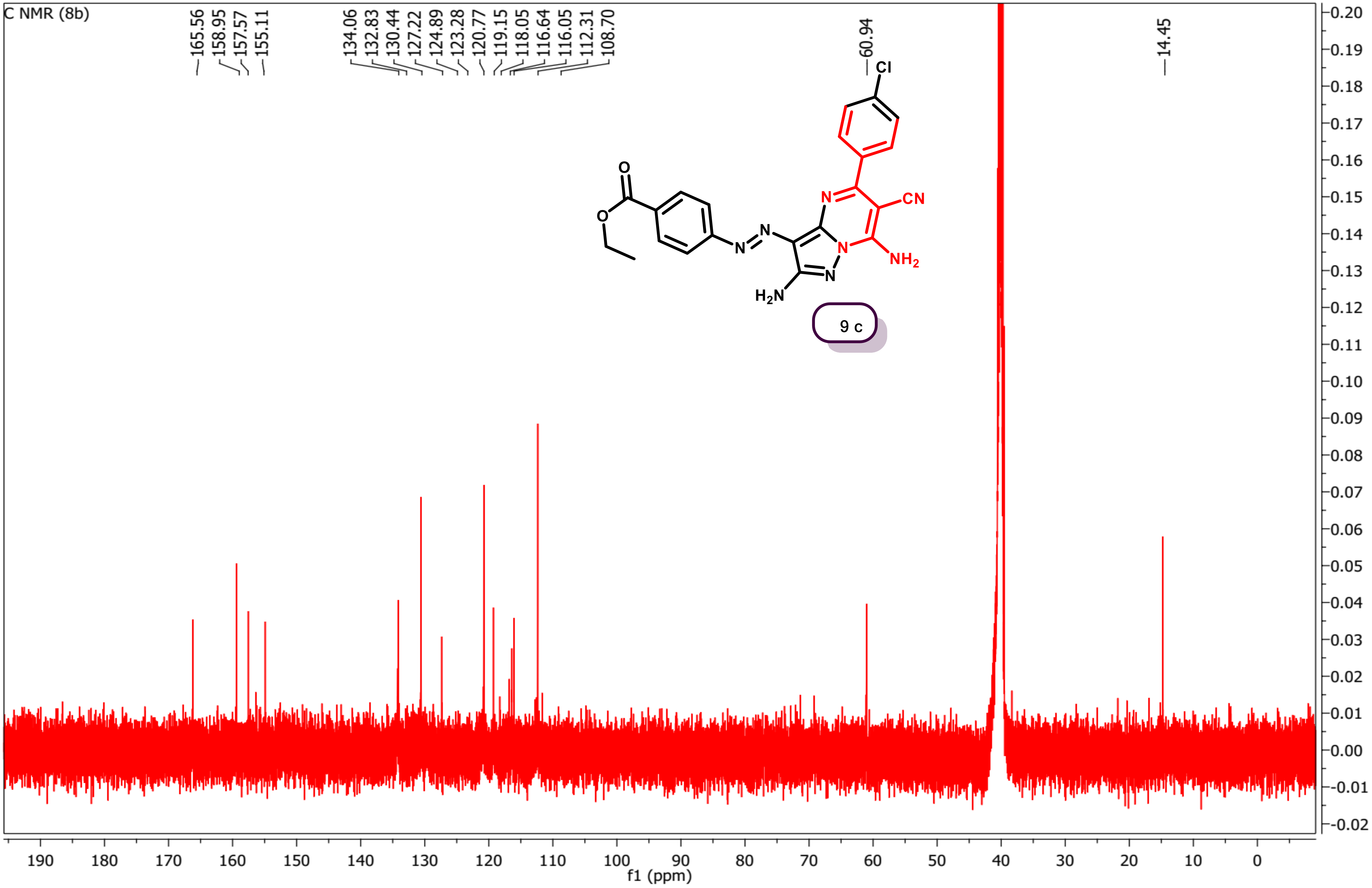
C NMR (8b)
165.56
158.95
157.57
155.11
134.06
132.83
130.44
127.22
124.89
123.28
120.77
119.15
118.05
116.64
116.05
112.31
108.70
60.94
14.45
Cl
O
O
CN
N
N
N
N
N
NH2
H2N
9 c
f1 (ppm)

[illegible]

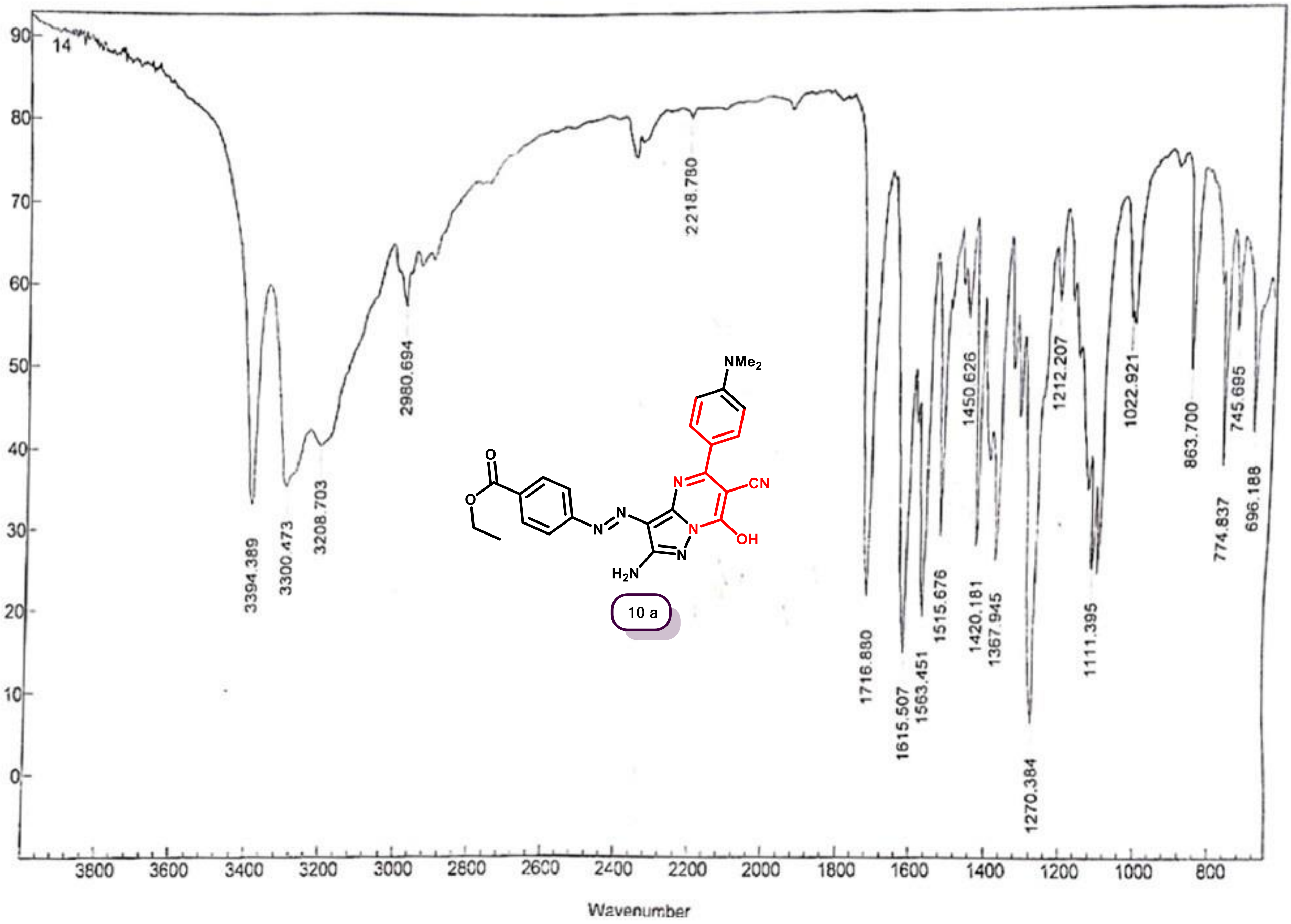

14
90
80
70
60
50
40
30
20
10
0
3394.389
3300.473
3208.703
2980.694
2218.780
1716.880
1615.507
1563.451
1515.676
1450.626
1420.181
1367.945
1270.384
1212.207
1111.395
1022.921
863.700
774.837
745.695
696.188
NMe2
CN
OH
N
N
N
N=N
H2N
O
10 a
3800
3600
3400
3200
3000
2800
2600
2400
2200
2000
1800
1600
1400
1200
1000
800
Wavenumber

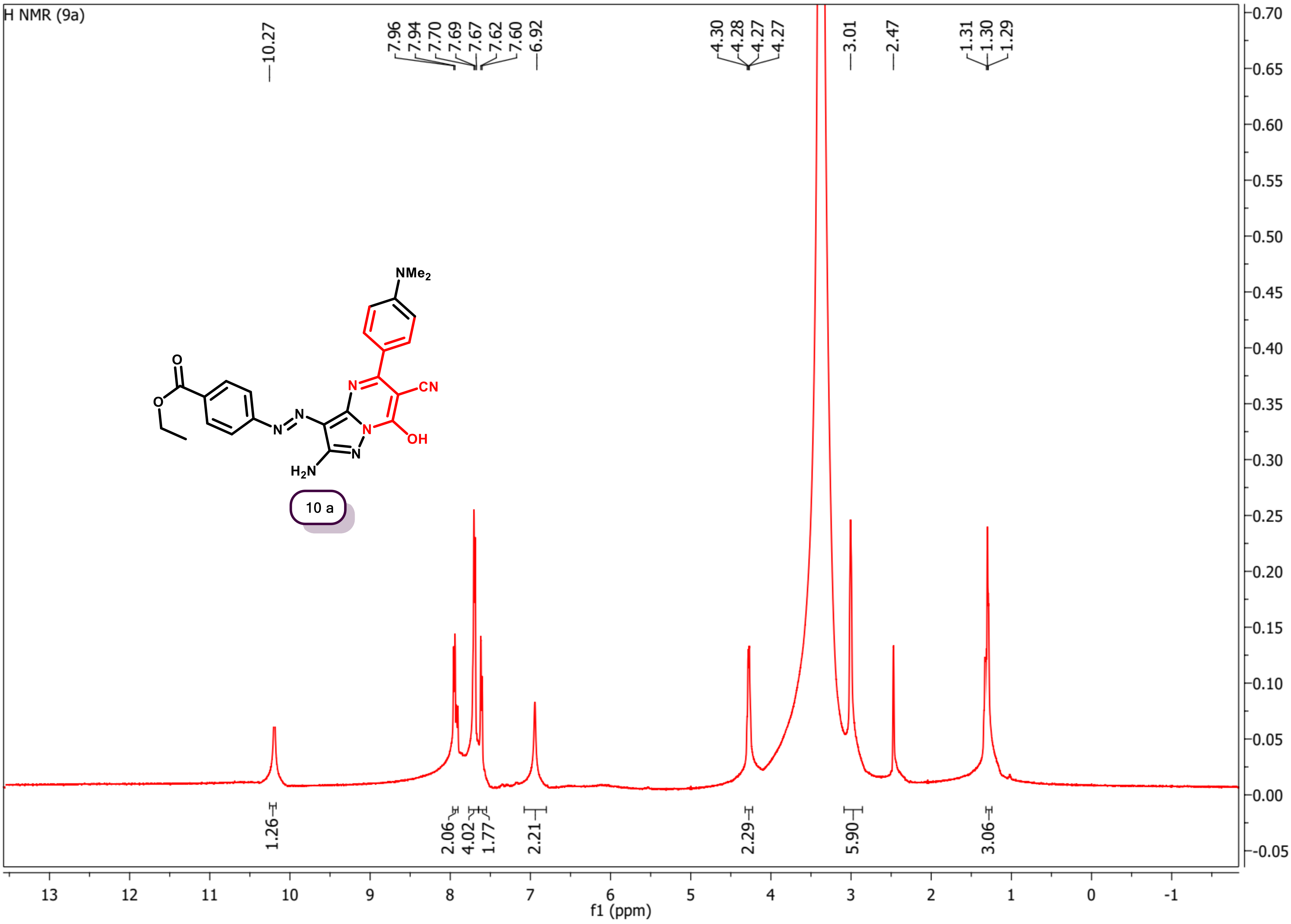
H NMR (9a)
10.27
7.96
7.94
7.70
7.69
7.67
7.62
7.60
6.92
4.30
4.28
4.27
4.27
3.01
2.47
1.31
1.30
1.29
NMe2
O
O
N
N
N
CN
N
N
OH
H2N
10 a
1.26
2.06
4.02
1.77
2.21
2.29
5.90
3.06
f1 (ppm)

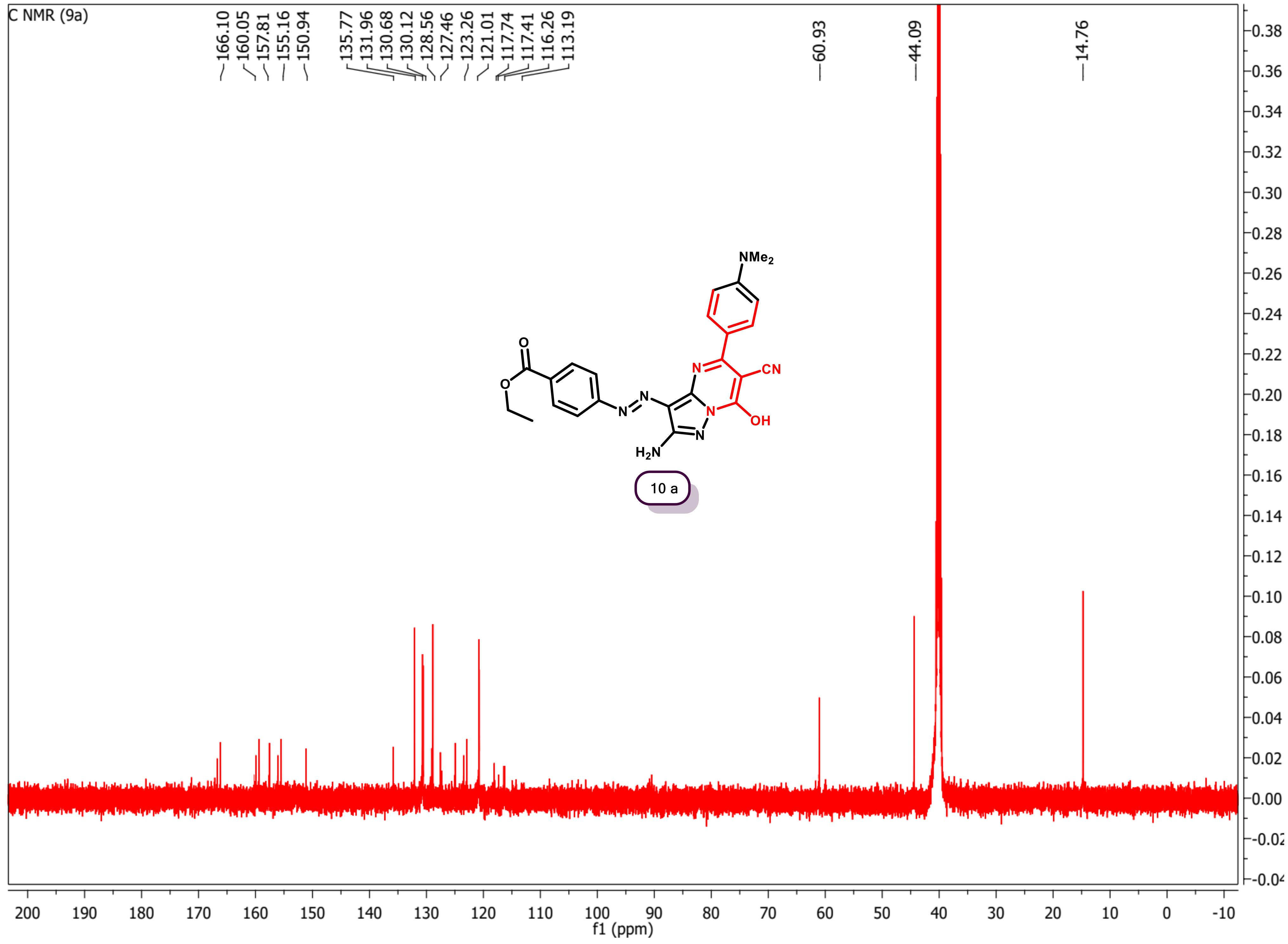

C NMR (9a)
166.10
160.05
157.81
155.16
150.94
135.77
131.96
130.68
130.12
128.56
127.46
123.26
121.01
117.74
117.41
116.26
113.19
60.93
44.09
14.76
NMe2
O
O
N
N
N
CN
N
OH
N
H2N
10 a
200
190
180
170
160
150
140
130
120
110
100
90
80
70
60
50
40
30
20
10
0
-10
f1 (ppm)

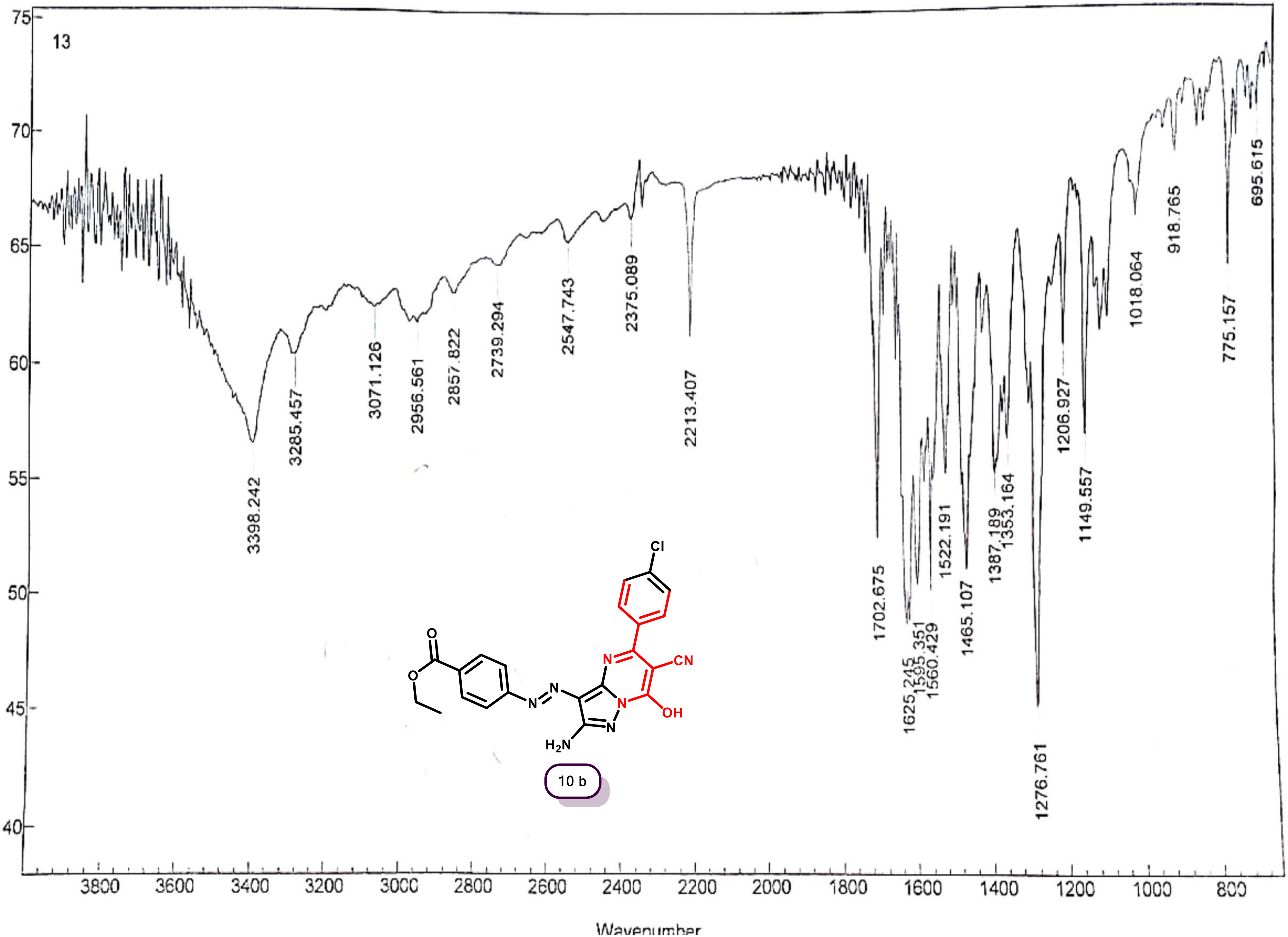
13
75
70
65
60
55
50
45
40
3398.242
3285.457
3071.126
2956.561
2857.822
2739.294
2547.743
2375.089
2213.407
1702.675
1625.245
1595.351
1560.429
1522.191
1465.107
1387.189
1353.164
1276.761
1206.927
1149.557
1018.064
918.765
775.157
695.615
3800
3600
3400
3200
3000
2800
2600
2400
2200
2000
1800
1600
1400
1200
1000
800
Wavenumber
Cl
O
O
N
N
N
N
N
CN
OH
H_2N
10 b

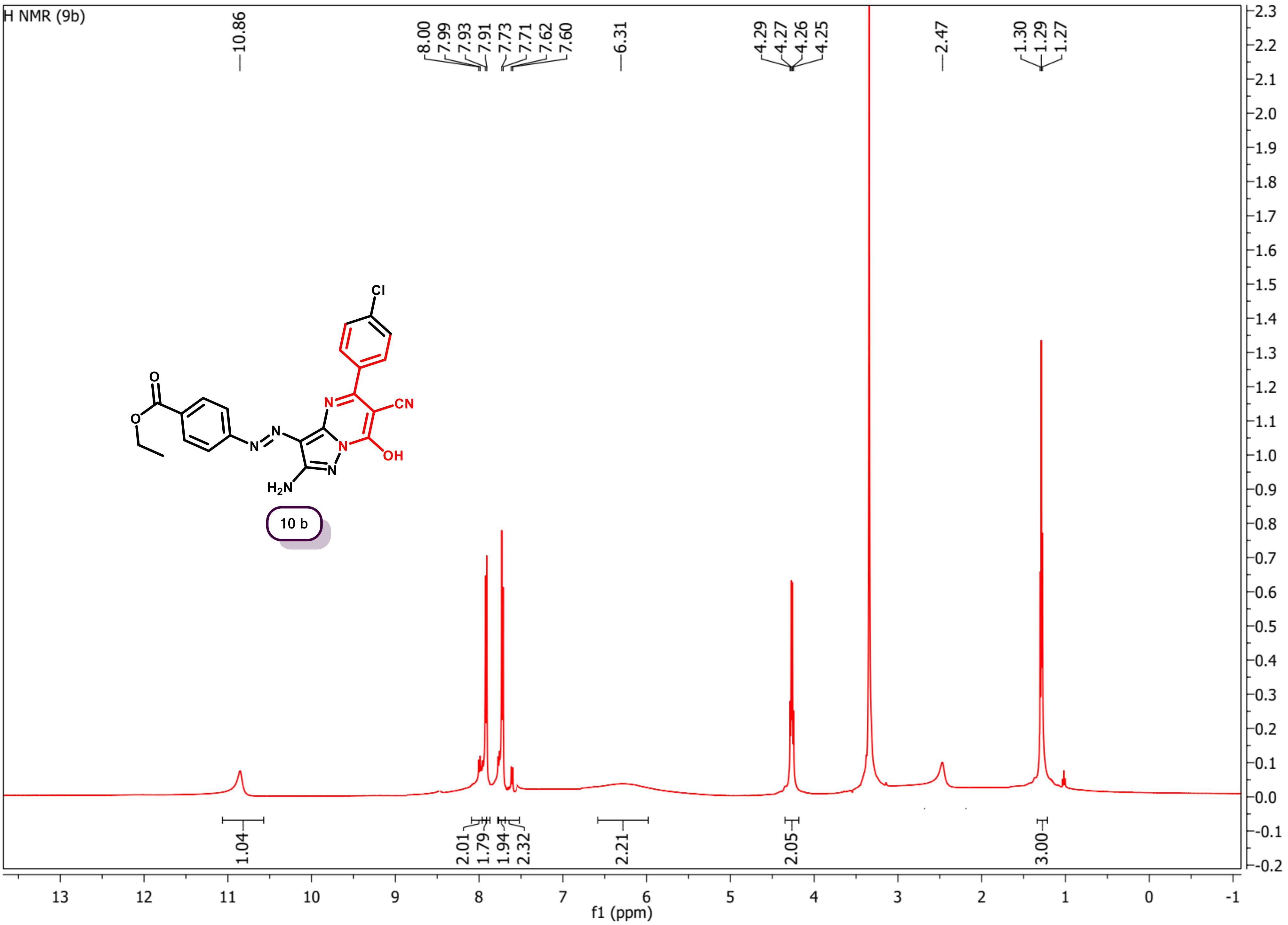
H NMR (9b)
10.86
8.00
7.99
7.93
7.91
7.73
7.71
7.62
7.60
6.31
4.29
4.27
4.26
4.25
2.47
1.30
1.29
1.27
Cl
O
O
N
N
N
N
N
CN
OH
H2N
10 b
1.04
2.01
1.79
1.94
2.32
2.21
2.05
3.00
f1 (ppm)

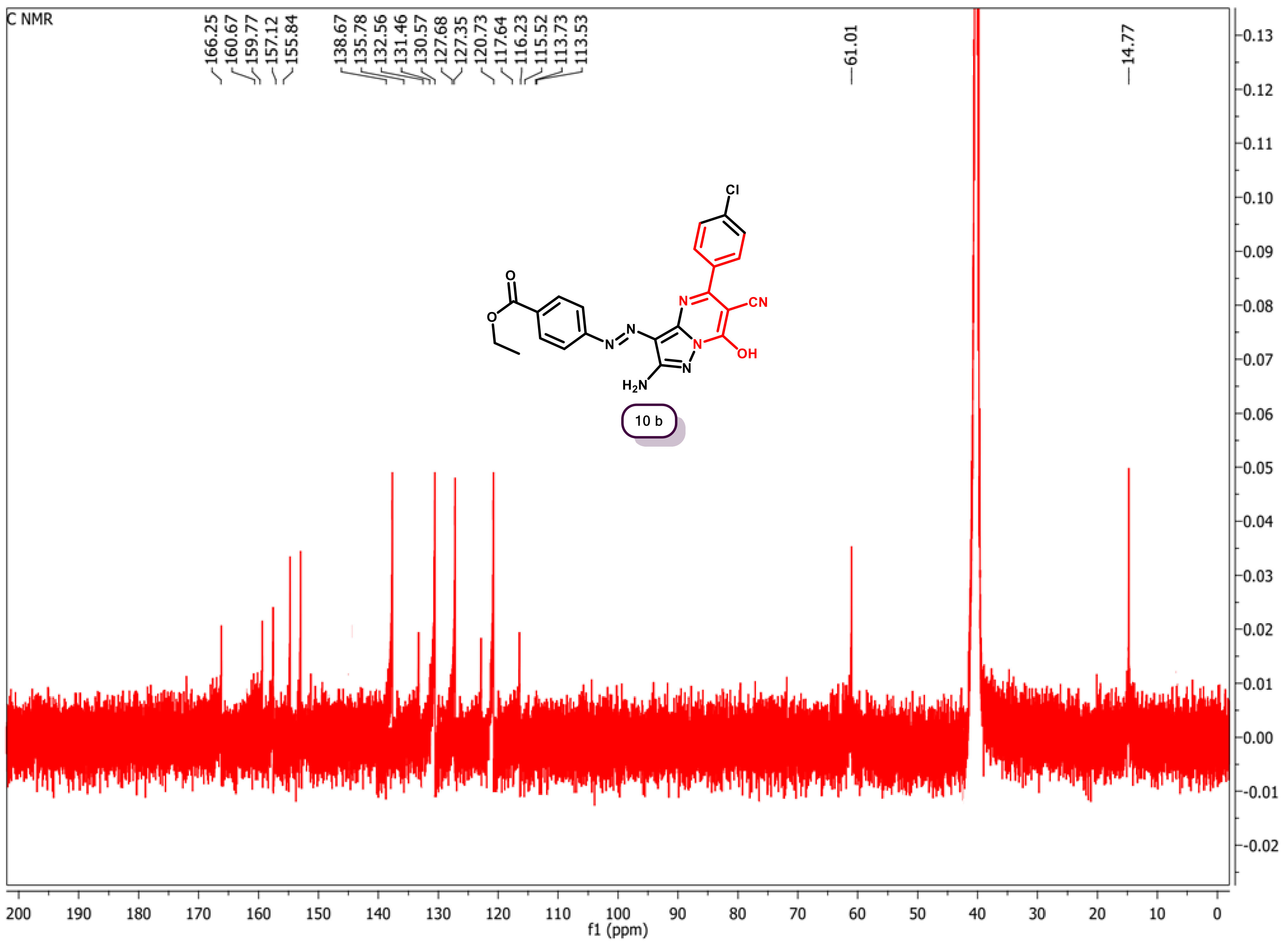

C NMR
166.25
160.67
159.77
157.12
155.84
138.67
135.78
132.56
131.46
130.57
127.68
127.35
120.73
117.64
116.23
115.52
113.73
113.53
61.01
14.77
Cl
O
O
N
N
N
N
N
CN
OH
H_2N
10 b
f1 (ppm)

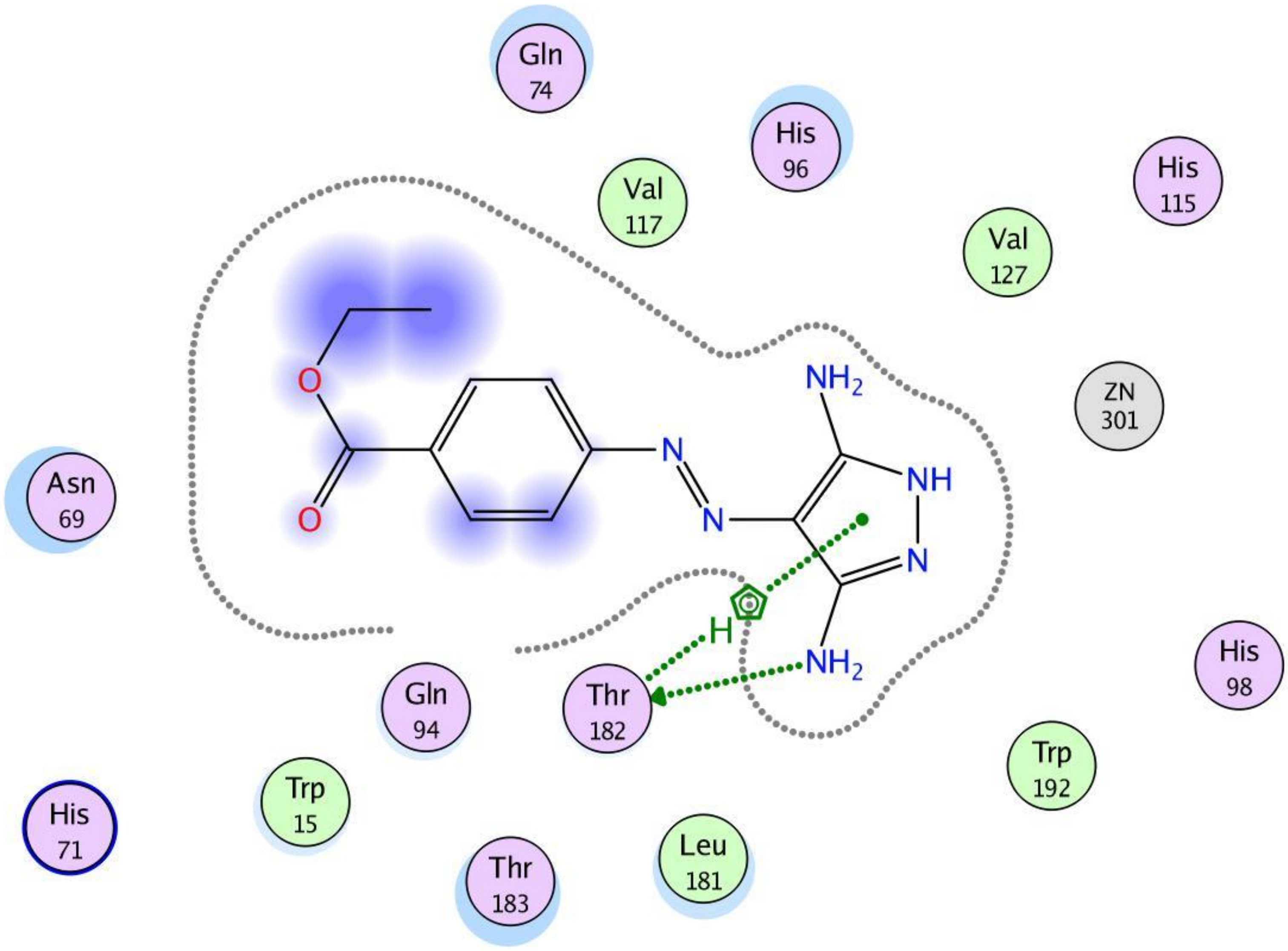

2D enzyme-ligand interaction of compounds **3a** inside the active site of alpha CA (PDB: 5HPJ)

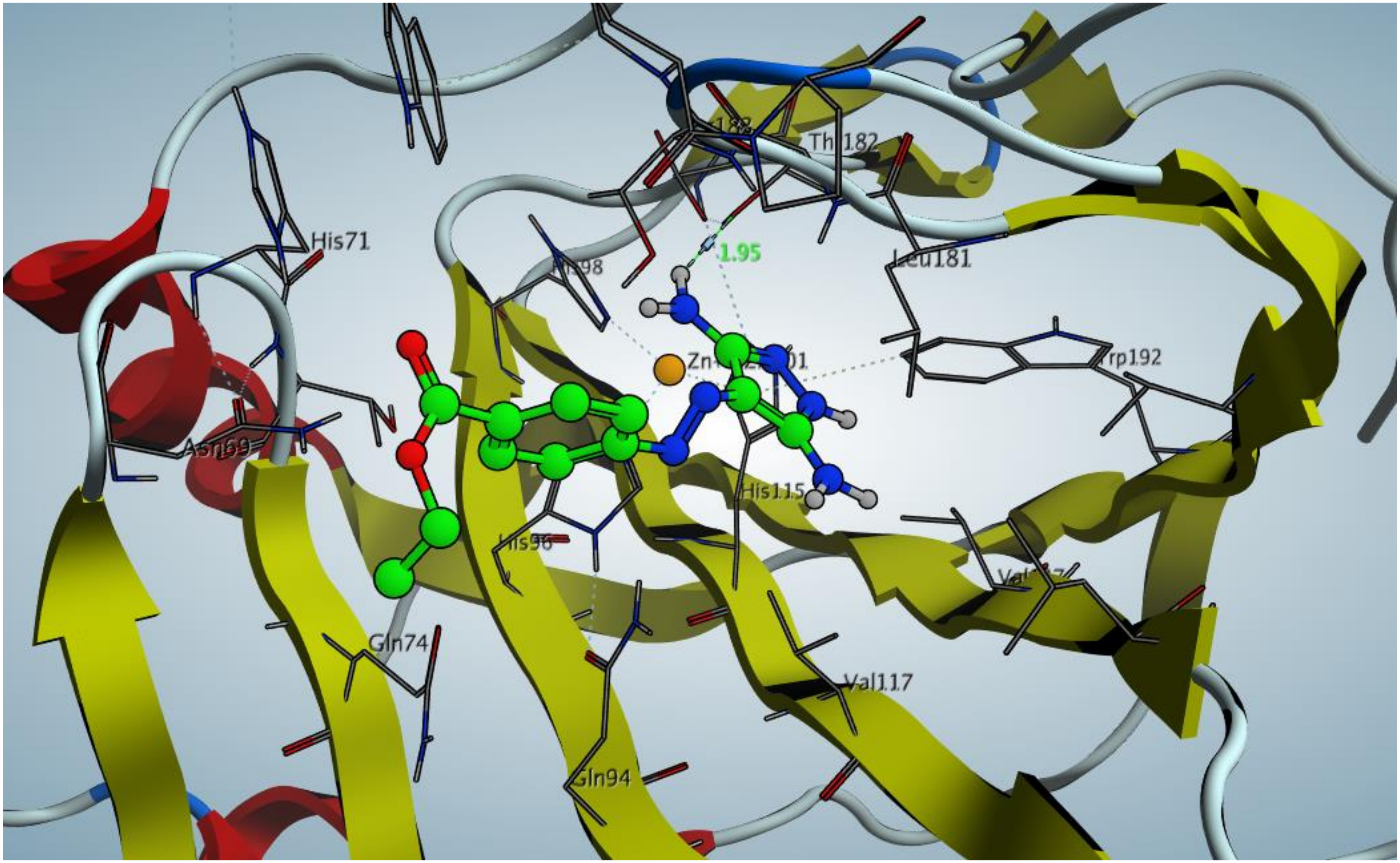

3D enzyme-ligand interaction of compounds **3a** inside the active site of alpha CA (PDB: 5HPJ)

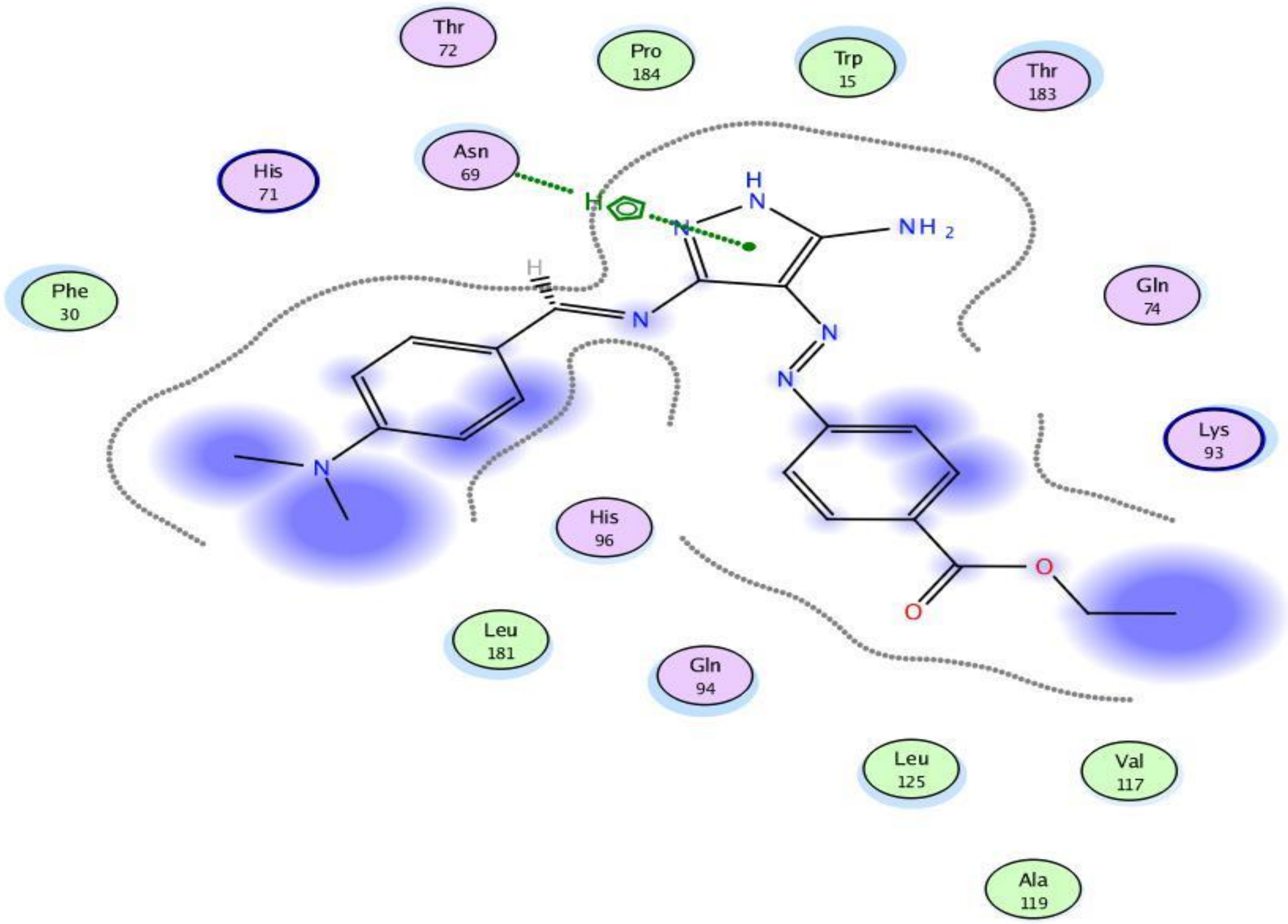

2D enzyme-ligand interaction of compounds **5a** inside the active site of alpha CA (PDB: 5HPJ)

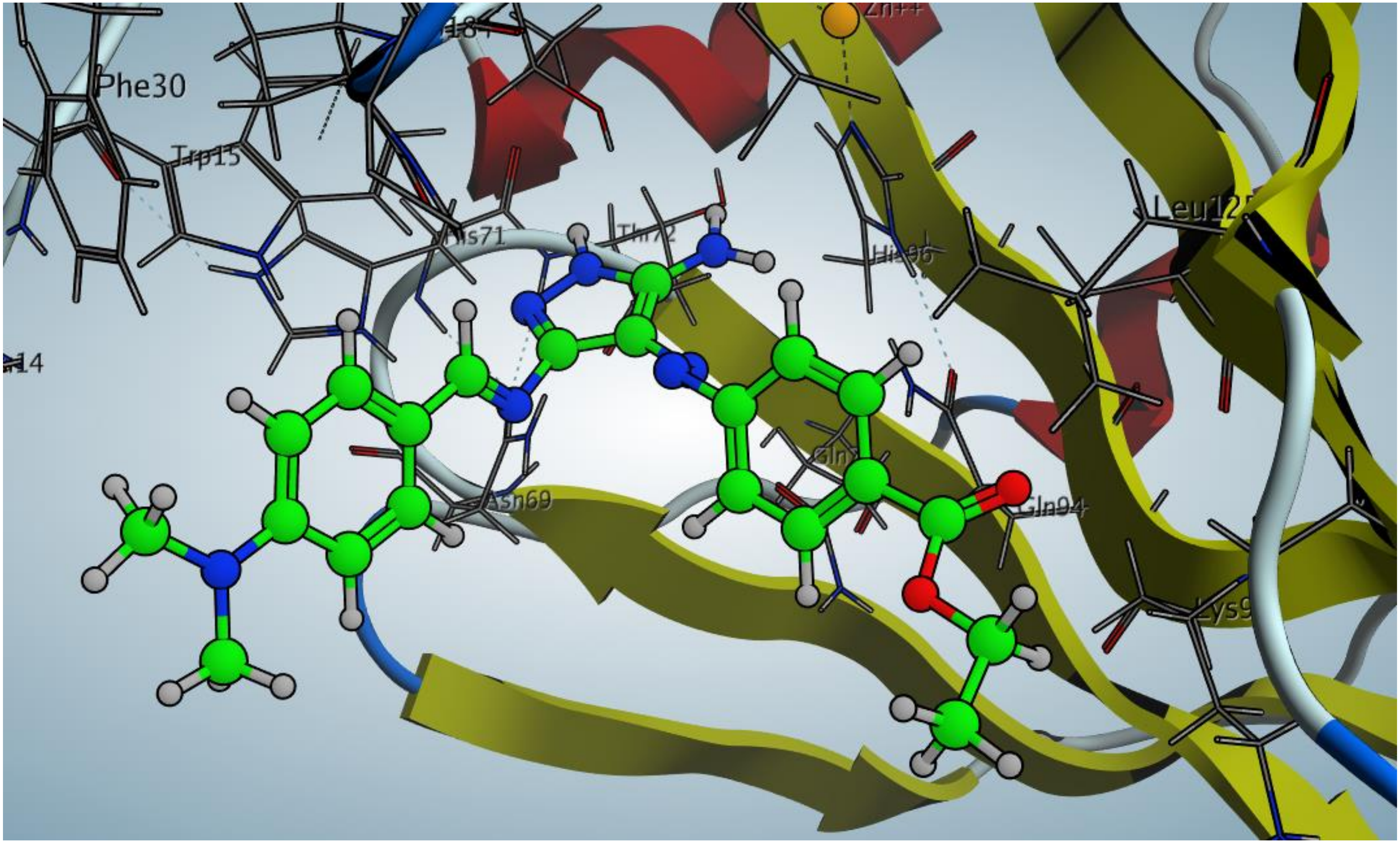

3D enzyme-ligand interaction of compounds **5a** inside the active site of alpha CA (PDB: 5HPJ)

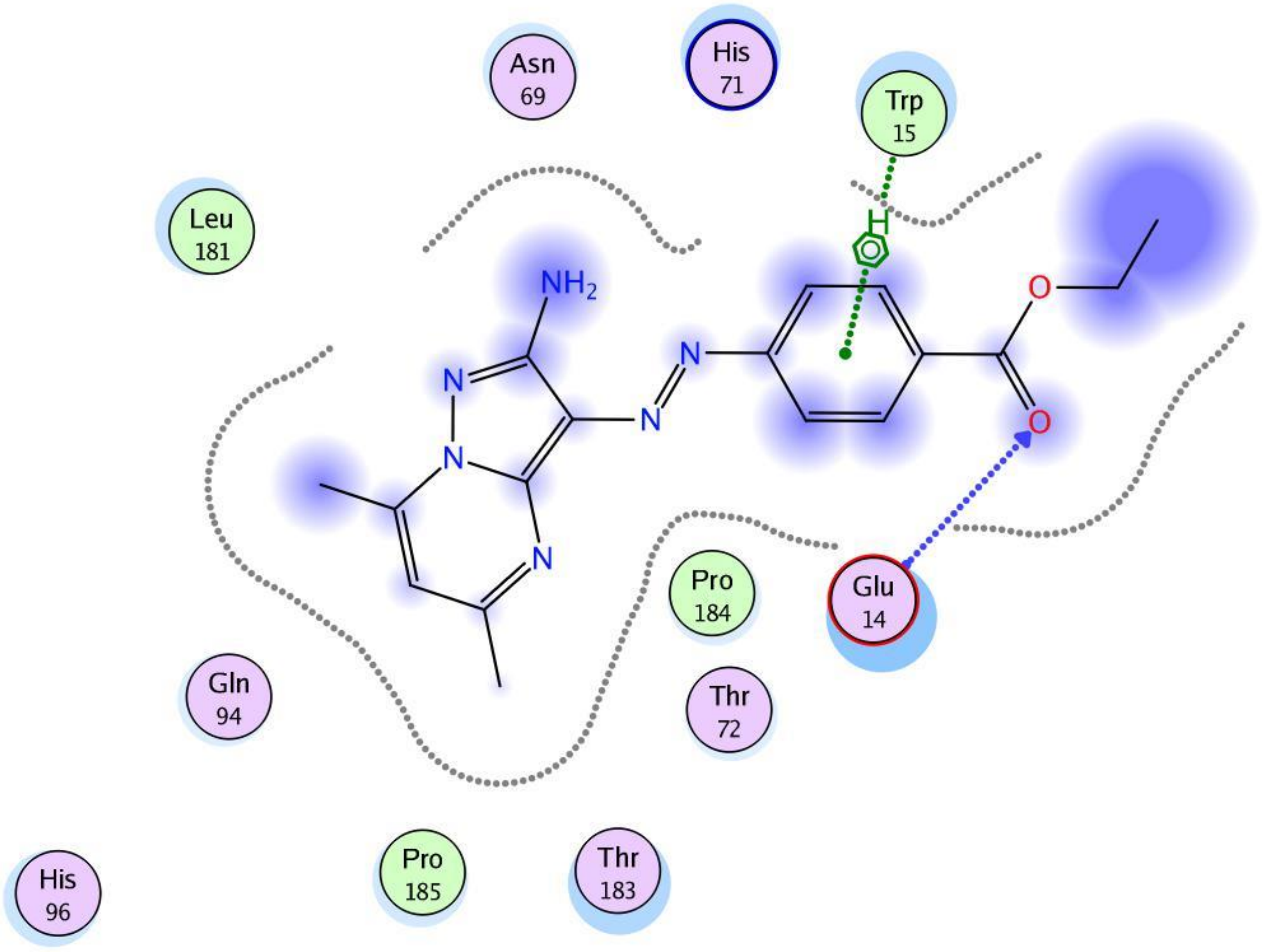

2D enzyme-ligand interaction of compounds **6** inside the active site of alpha CA (PDB: 5HPJ)

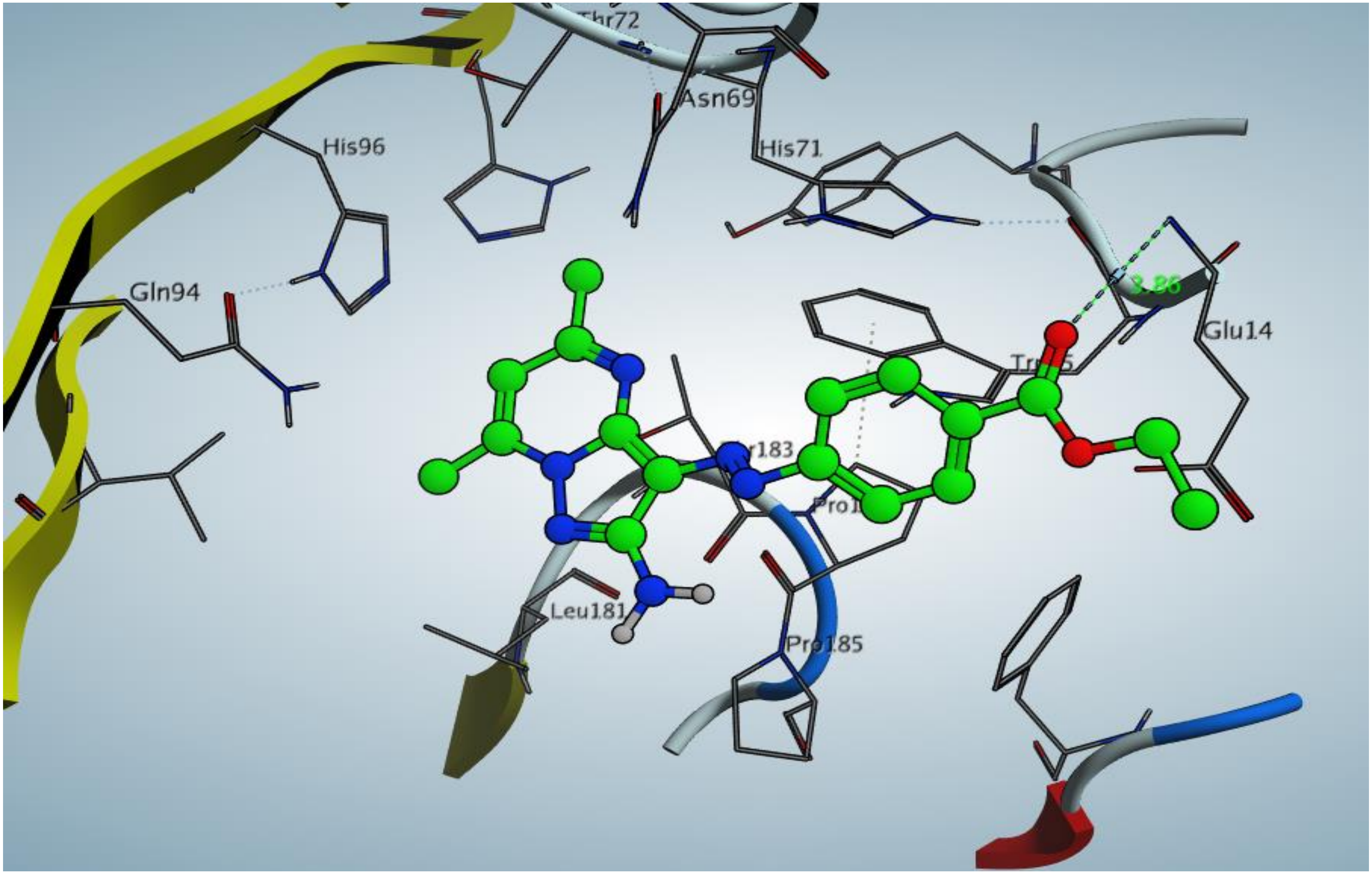

3D enzyme-ligand interaction of compounds **6** inside the active site of alpha CA (PDB: 5HPJ)

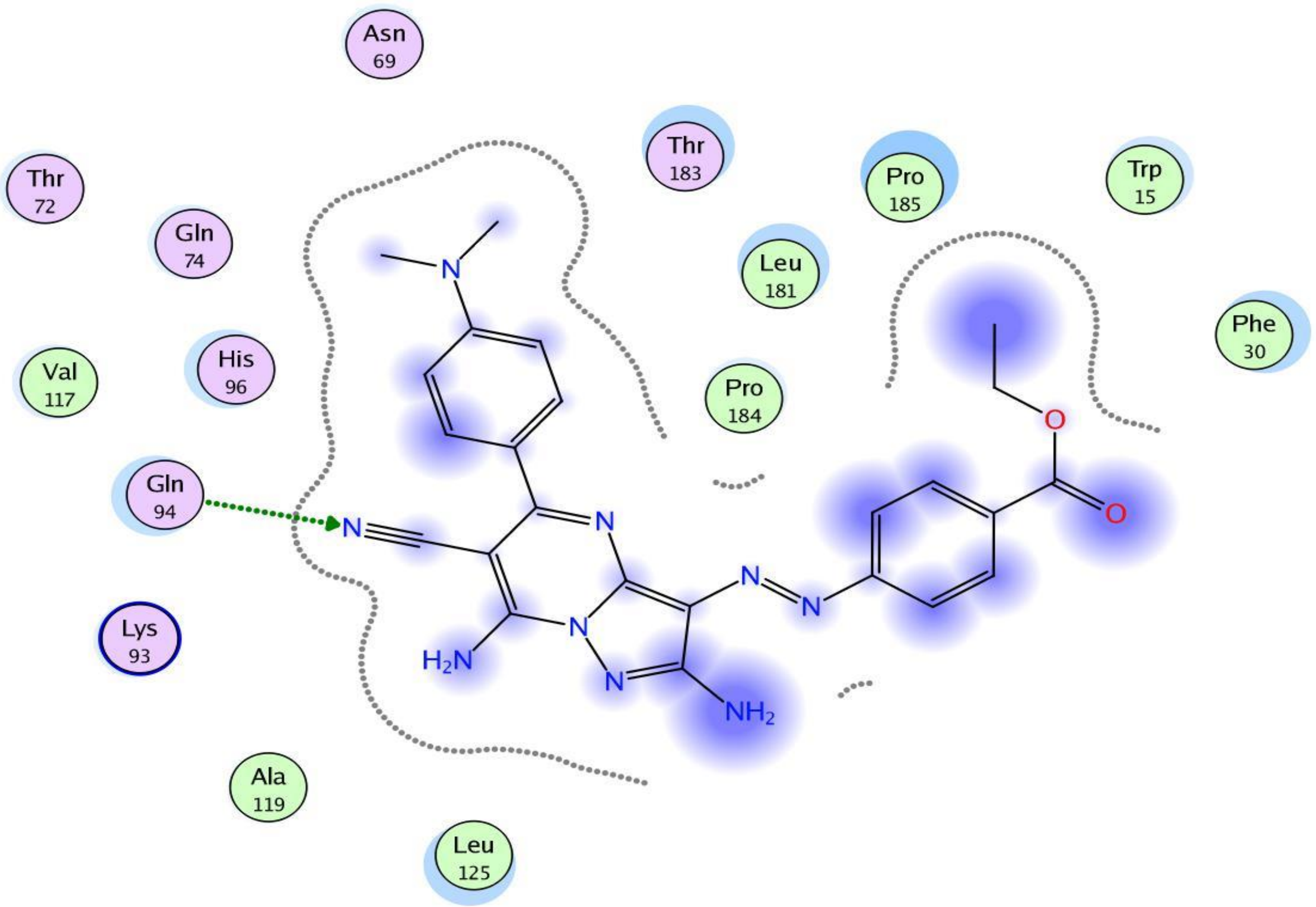

2D enzyme-ligand interaction of compounds **9a** inside the active site of alpha CA (PDB: 5HPJ)

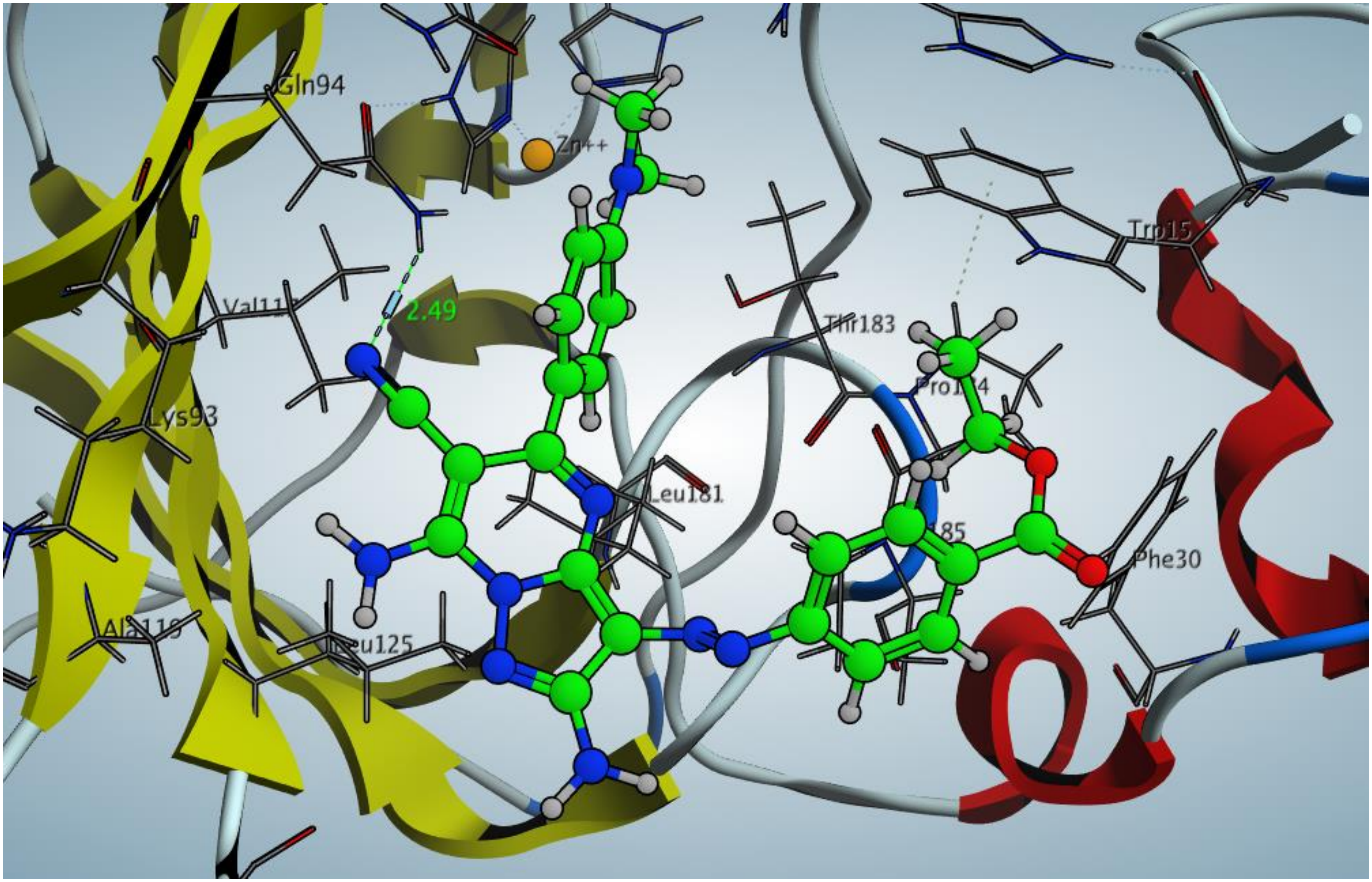

3D enzyme-ligand interaction of compounds **9a** inside the active site of alpha CA (PDB: 5HPJ)

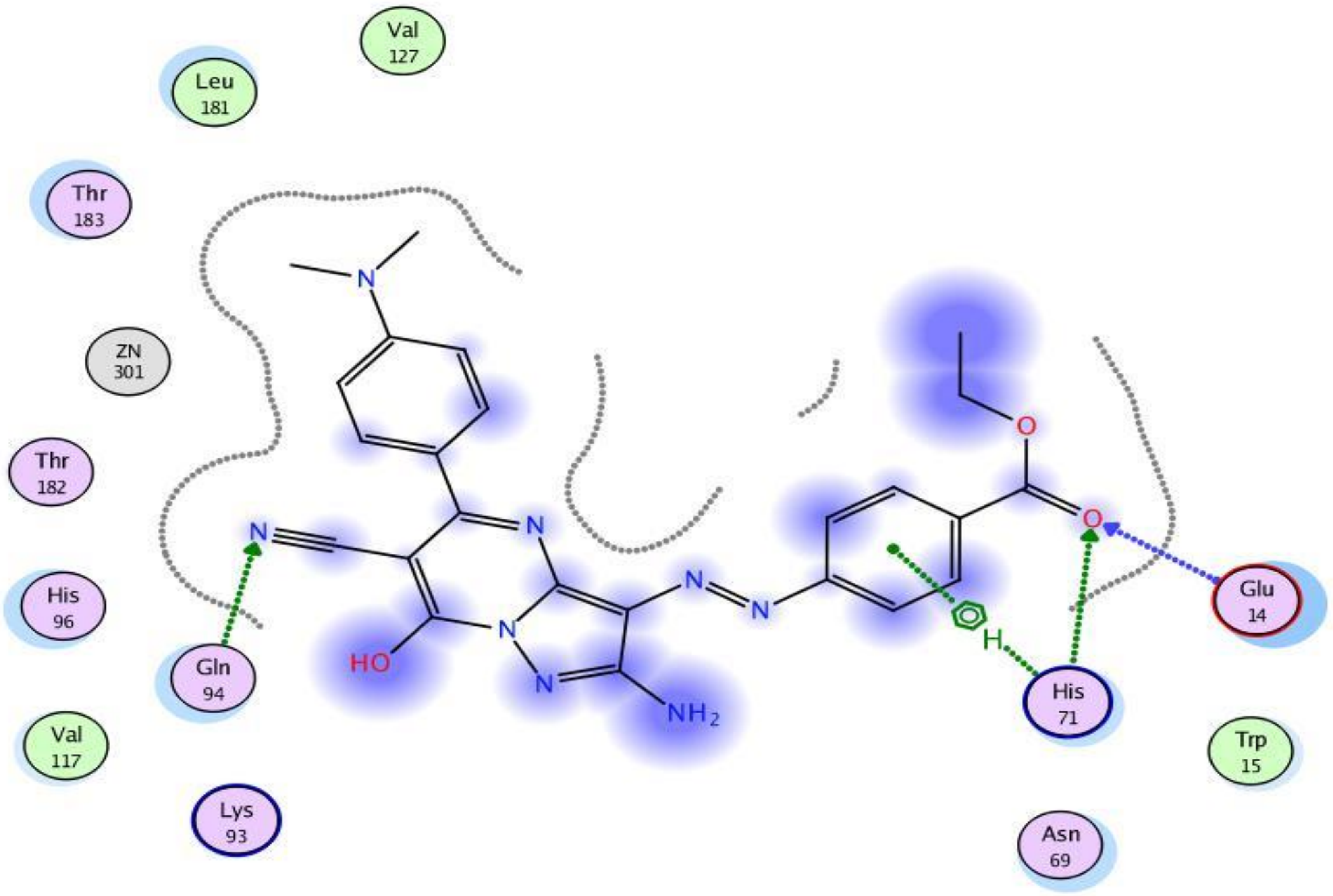

2D enzyme-ligand interaction of compounds **10a** inside the active site of alpha CA (PDB: 5HPJ)

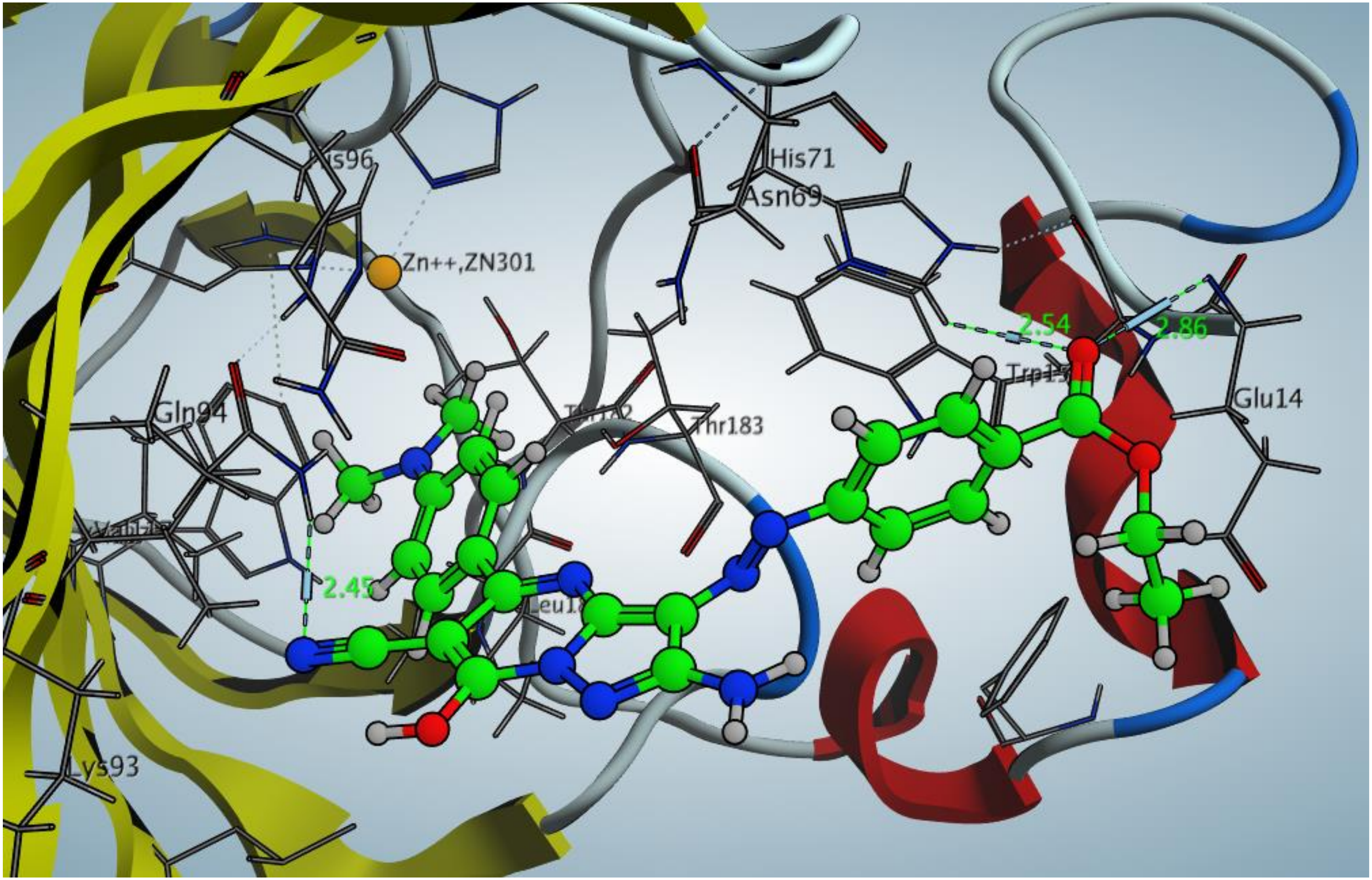

3D enzyme-ligand interaction of compounds **10a** inside the active site of alpha CA (PDB: 5HPJ)

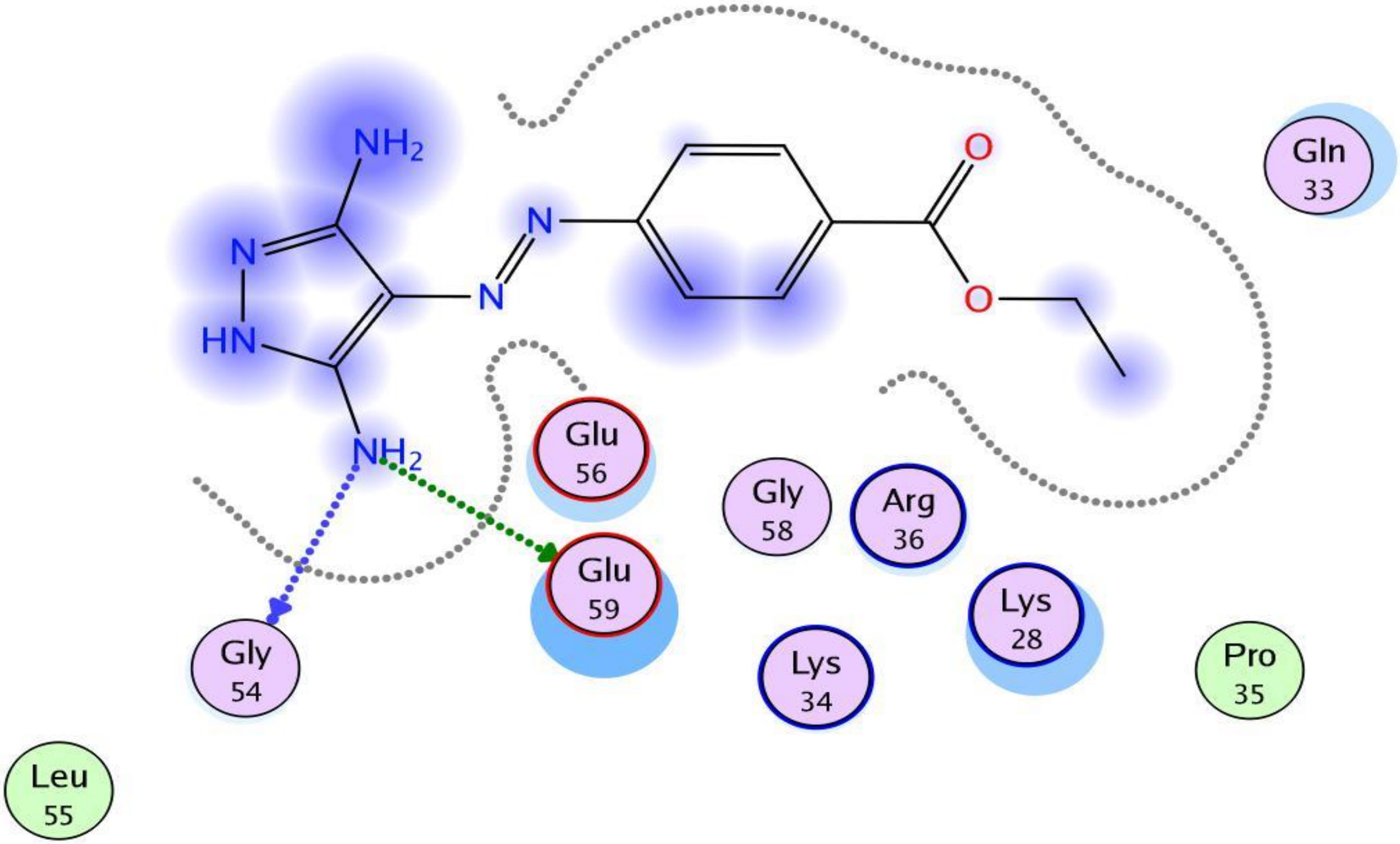

2D enzyme-ligand interaction of compounds 3a inside the active site of beta CA (PDB: 1I6P)

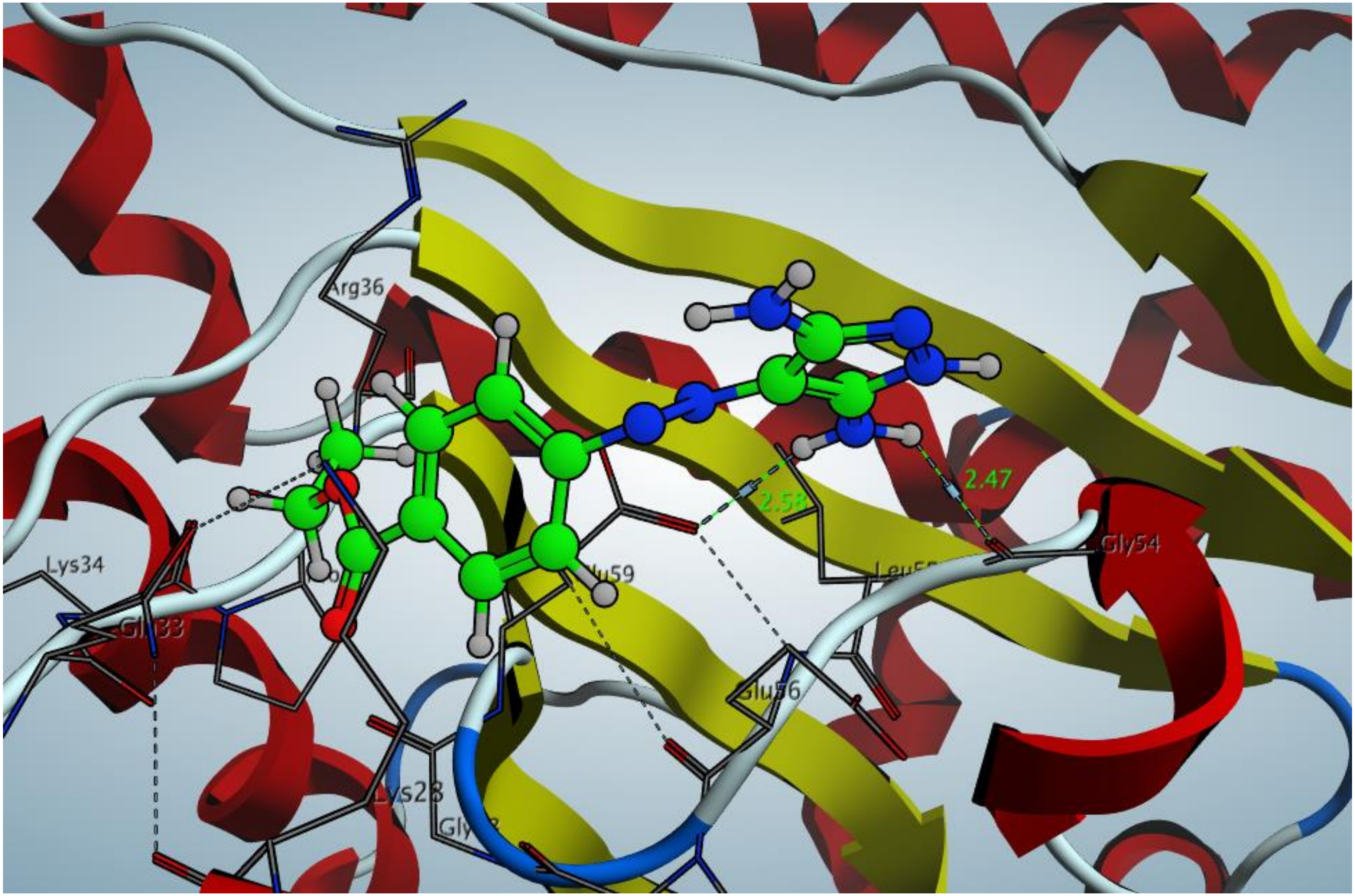

3D enzyme-ligand interaction of compounds 3a inside the active site of beta CA (PDB: 1I6P)

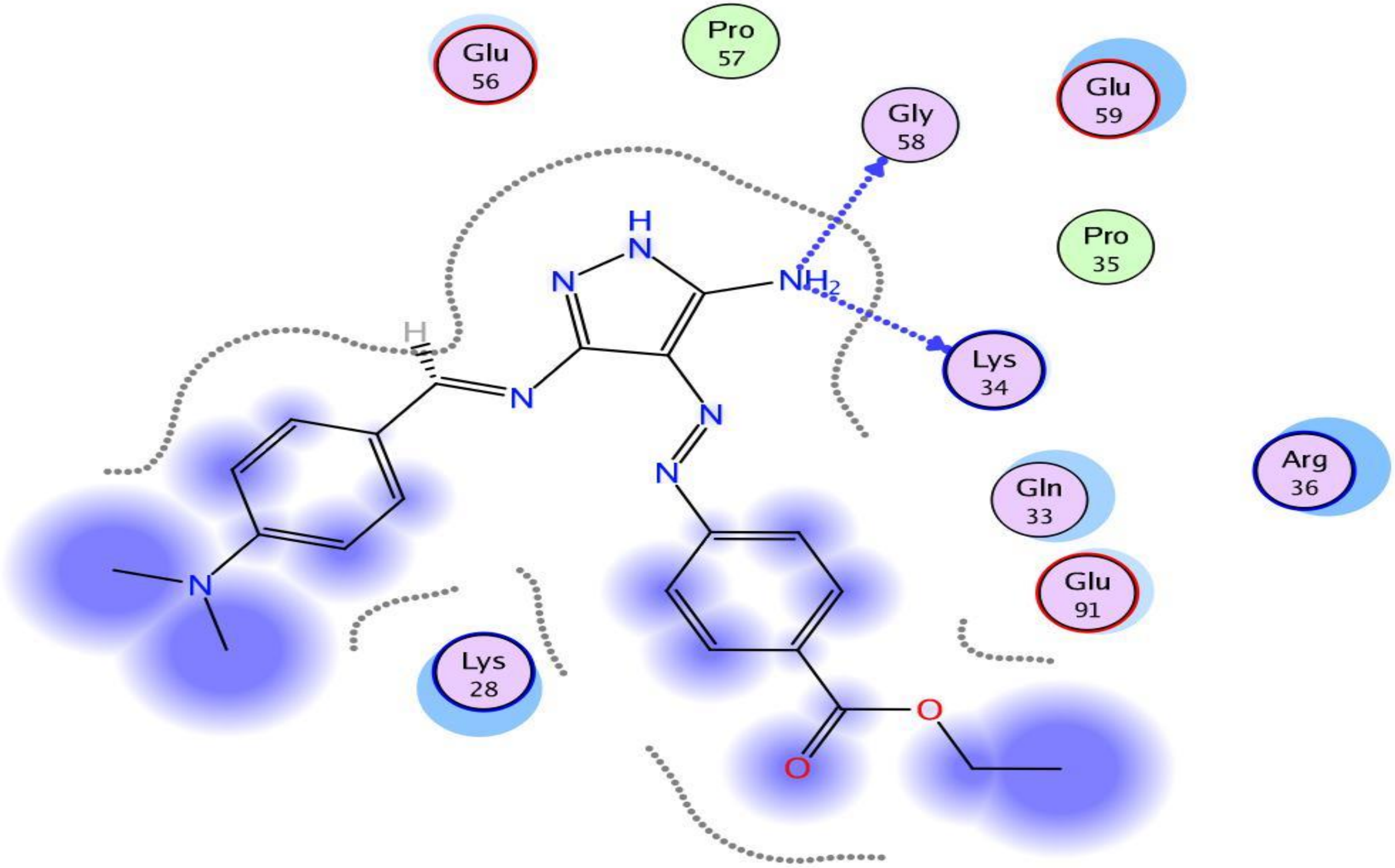

2D enzyme-ligand interaction of compounds 5a inside the active site of beta CA (PDB: 1I6P)

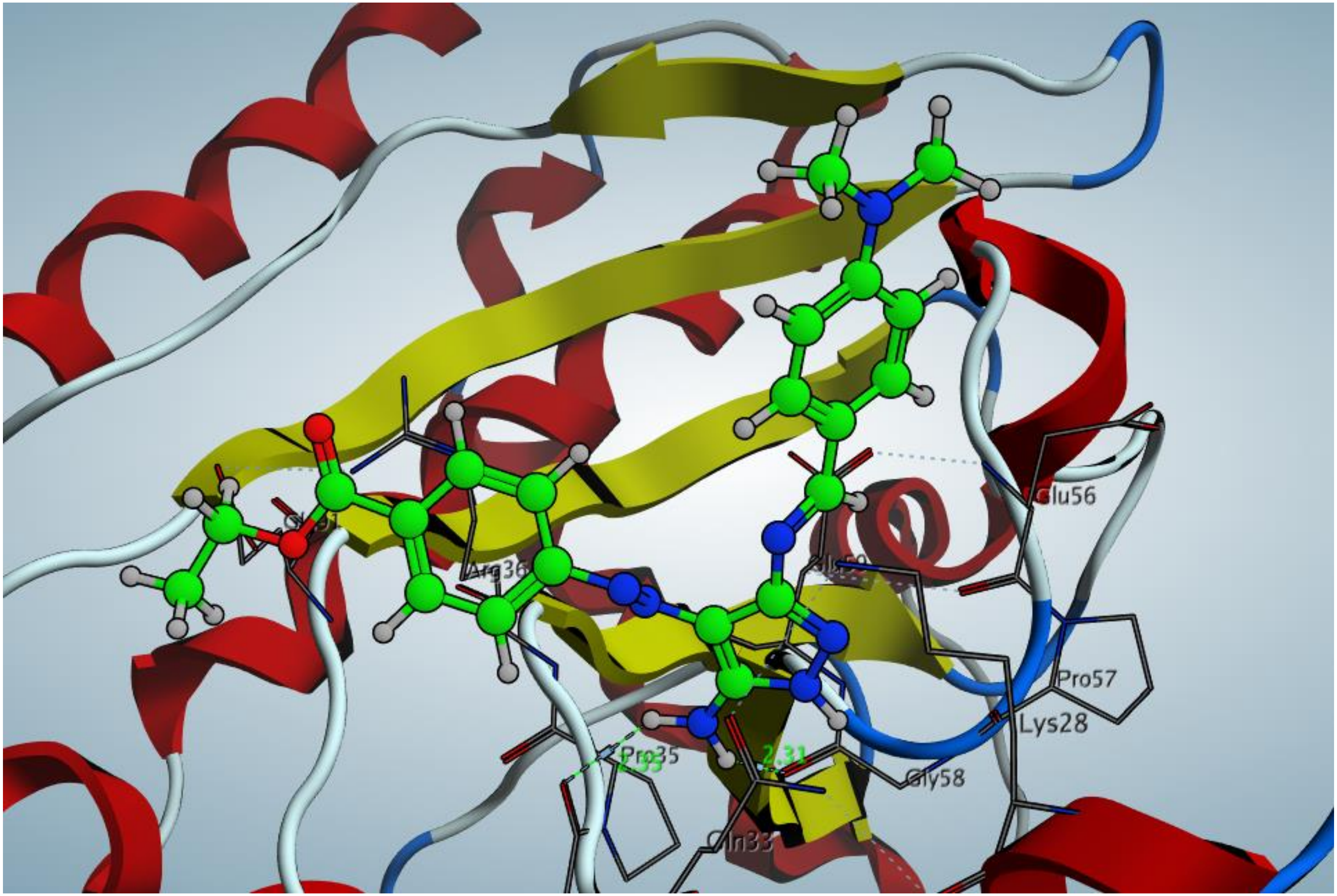

3D enzyme-ligand interaction of compounds 5a inside the active site of beta CA (PDB: 1I6P)

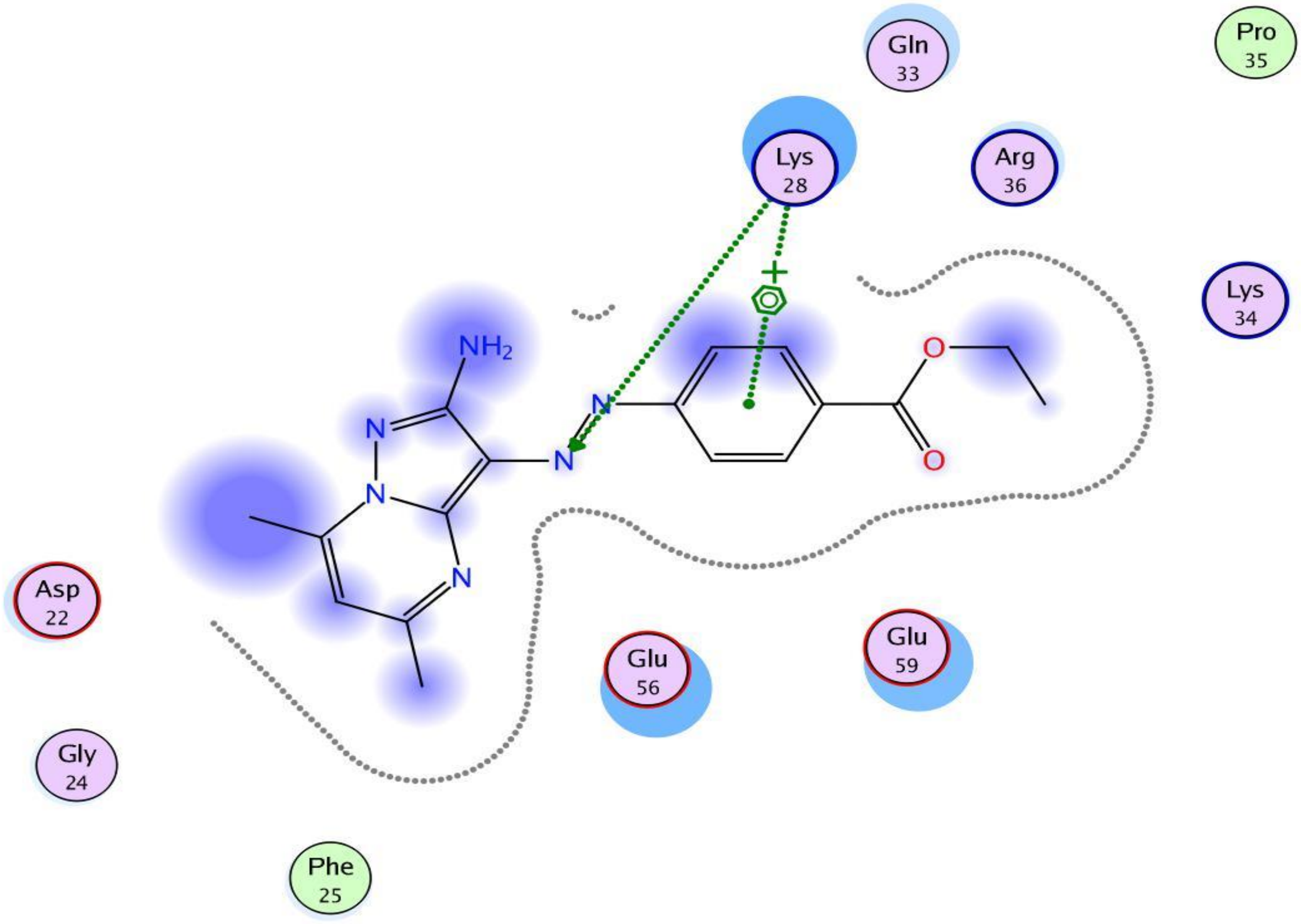

2D enzyme-ligand interaction of compounds 6 inside the active site of beta CA (PDB: 1I6P)

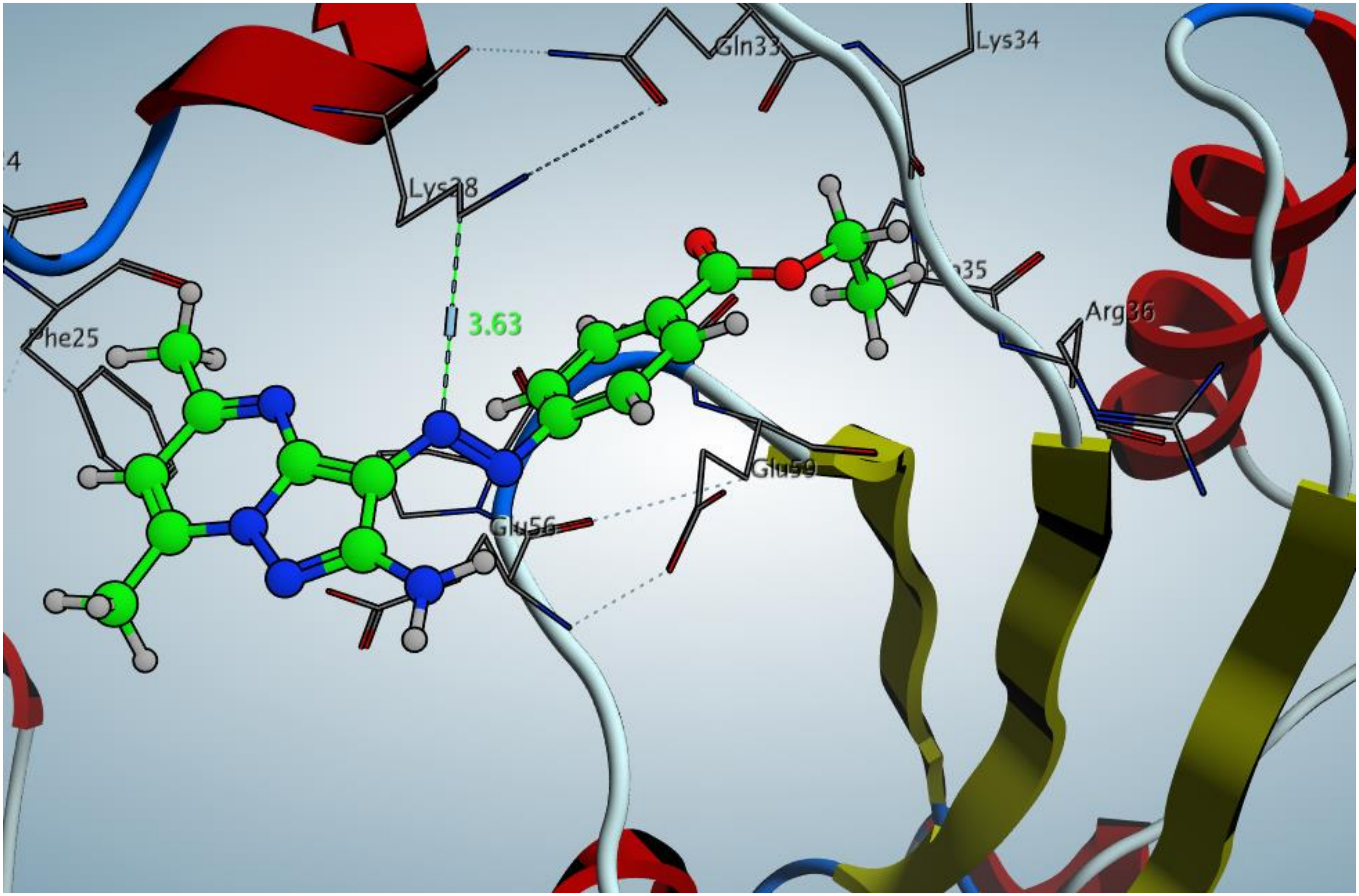

3D enzyme-ligand interaction of compounds 6 inside the active site of beta CA (PDB: 1I6P)

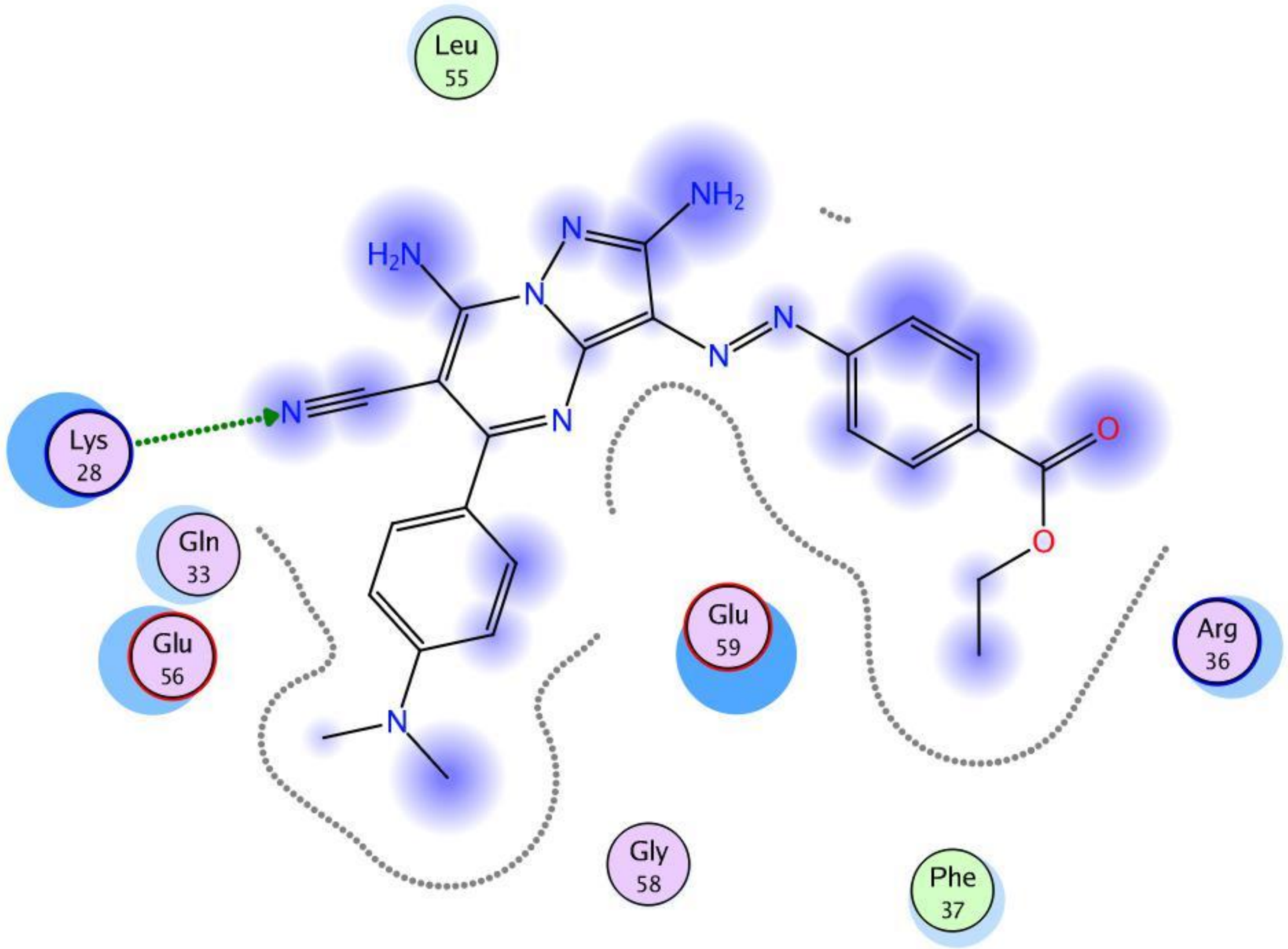

2D enzyme-ligand interaction of compounds 9a inside the active site of beta CA (PDB: 1I6P)

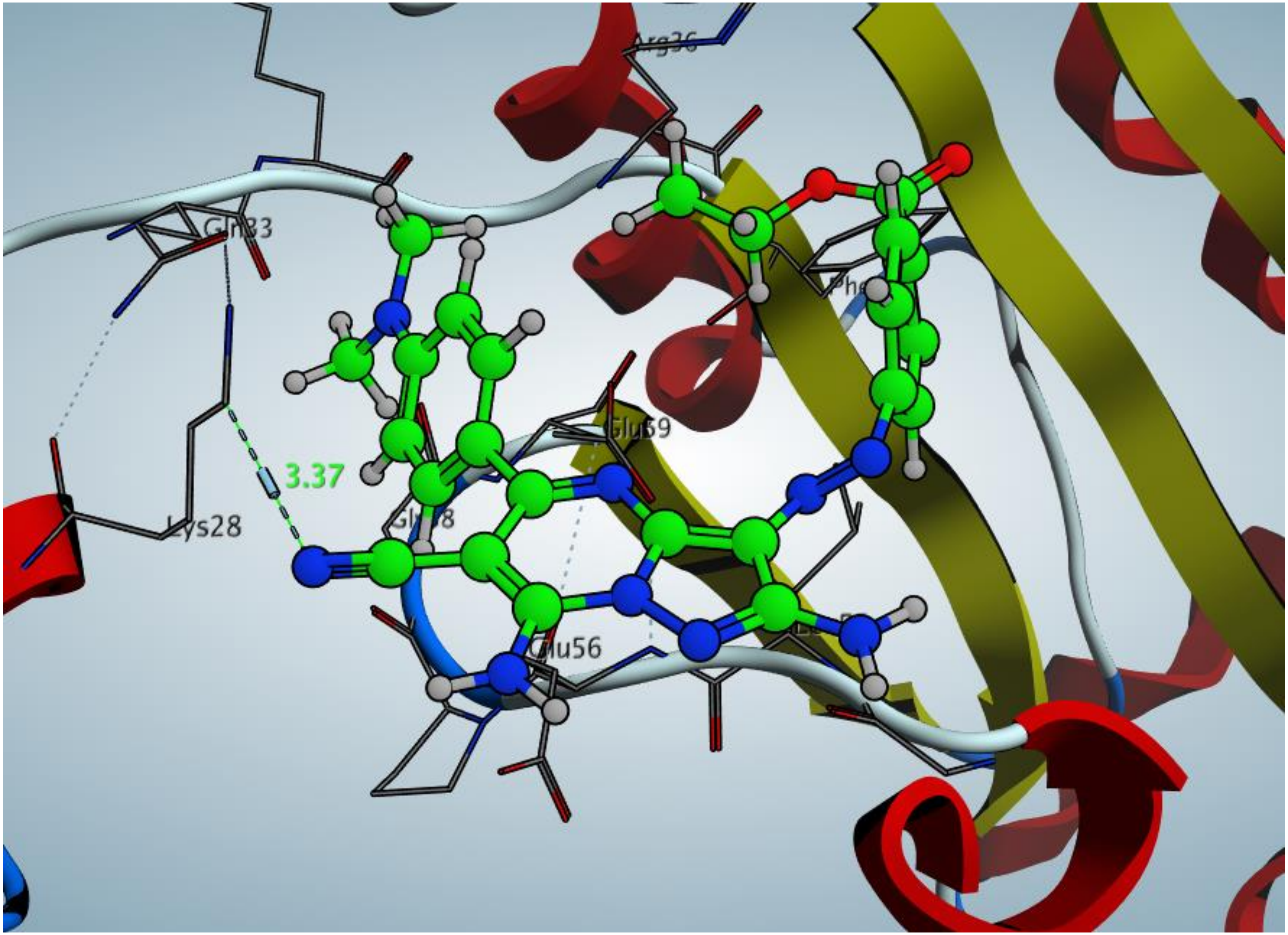

3D enzyme-ligand interaction of compounds 9a inside the active site of beta CA (PDB: 1I6P)

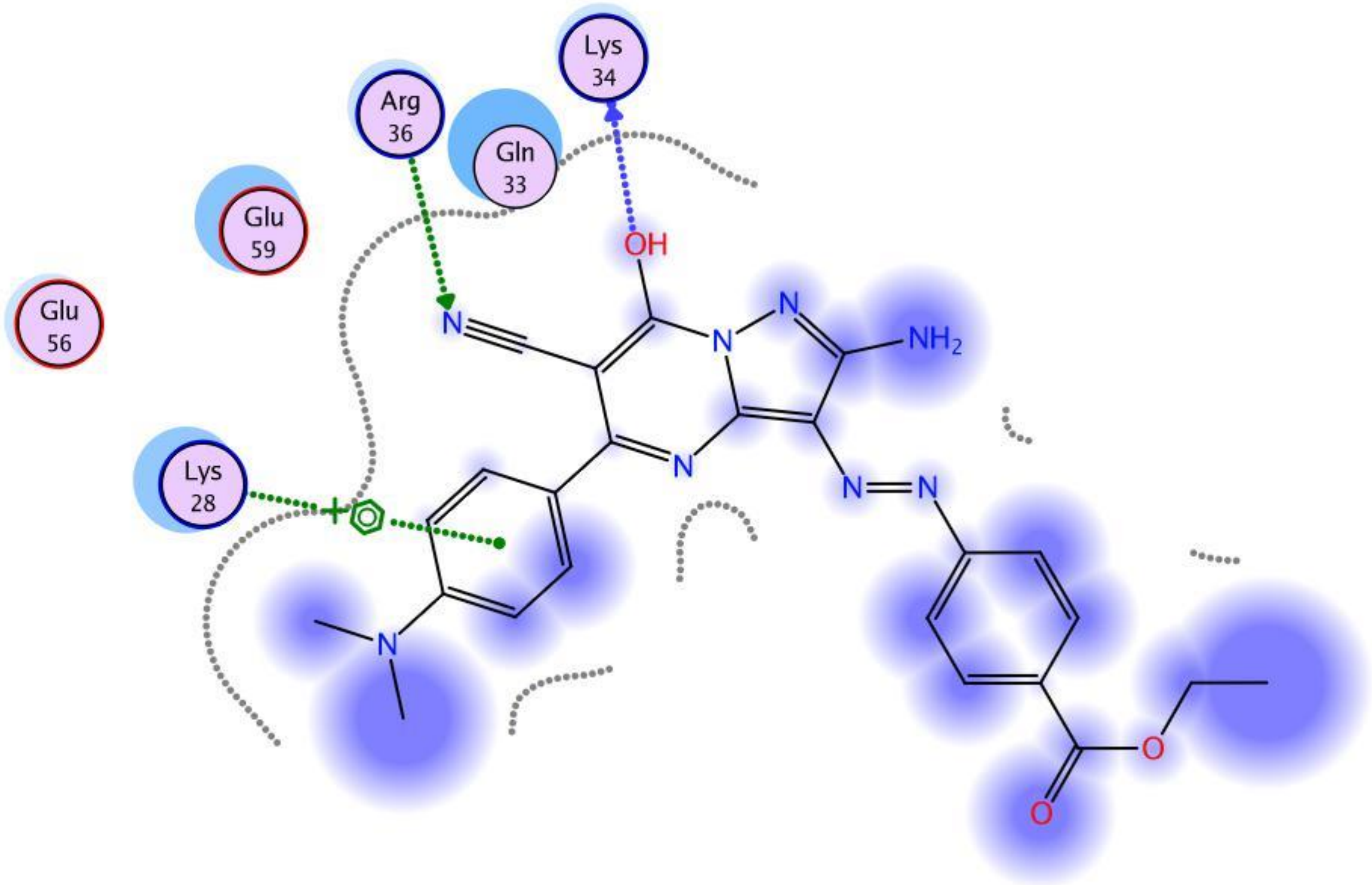

2D enzyme-ligand interaction of compounds 10a inside the active site of beta CA (PDB: 1I6P)

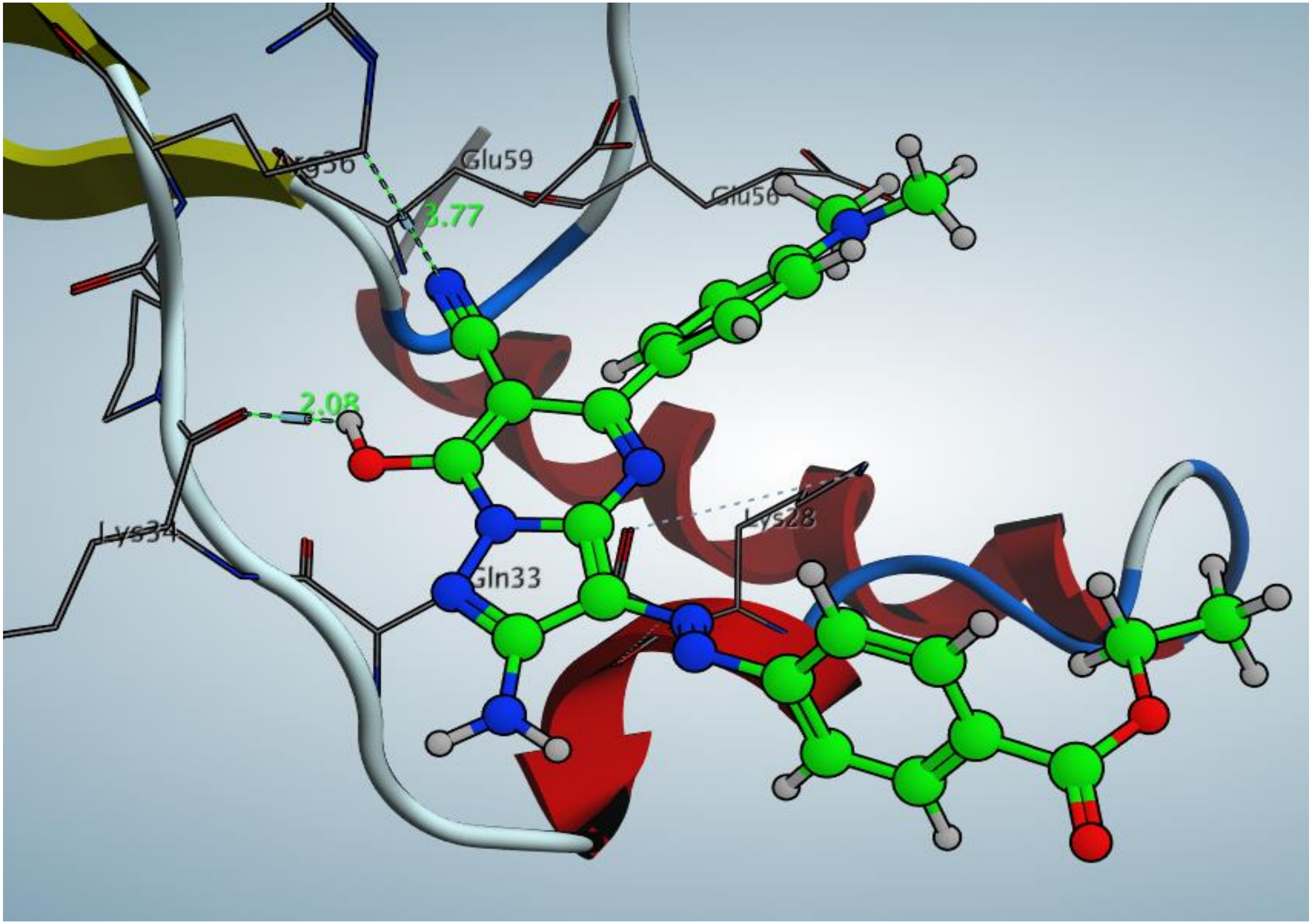

3D enzyme-ligand interaction of compounds ***10a*** inside the active site of beta CA (PDB: 1I6P)
